# Asymmetric synthesis of *N*-allylic indoles via regio- and enantioselective allylation of aryl hydrazines

**DOI:** 10.1038/ncomms8616

**Published:** 2015-07-03

**Authors:** Kun Xu, Thomas Gilles, Bernhard Breit

**Affiliations:** 1 Institut für Organische Chemie, Albert-Ludwigs-Universität Freiburg, Albertstrasse 21, Freiburg im Breisgau 79104, Germany

## Abstract

The asymmetric synthesis of *N*-allylic indoles is important for natural product synthesis and pharmaceutical research. The regio- and enantioselective *N*-allylation of indoles is a true challenge due to the favourable C3-allylation. We develop here a new strategy to the asymmetric synthesis of *N*-allylic indoles via rhodium-catalysed *N*-selective coupling of aryl hydrazines with allenes followed by Fischer indolization. The exclusive *N*-selectivities and good to excellent enantioselectivities are achieved applying a rhodium(I)/DTBM-Segphos or rhodium(I)/DTBM-Binap catalyst. This method permits the practical synthesis of valuable chiral *N*-allylated indoles, and avoids the *N*- or *C*-selectivity issue.

## Abstract (web summary)

*N*-allylic indoles are important in medicinal and synthetic chemistry, but selective *N*-allylation of indoles can be difficult to achieve. Here, the authors report a catalytic asymmetric synthesis of these compounds through an enantioselective addition of aryl hydrazines to allenes, followed by indole formation.

## Graphical abstract

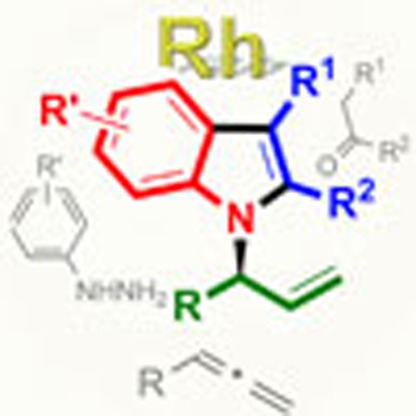

The asymmetric synthesis of indoles is of great interest because of their prevalence in bioactive molecules^1^,^2^,^3^,^4^,^5^,^6^. In particular, indoles bearing a α-chiral carbon centre on the *N* are important structural motifs in natural products and pharmaceutical drugs (Fig. 1a)^7^,^8^,^9^,^10^,^11^,^12^. For this reason, extensive efforts have been undertaken to explore the catalytic asymmetric allylation of indoles^13^,^14^,^15^,^16^,^17^,^18^,^19^,^20^,^21^,^22^,^23^,^24^. However, selective *N*-allylation of indoles is a true challenge due to the high nucleophilicity of C3 of the indole nucleus and the weak acidity of the N–H bond (Fig. 1b)^25^,^26^. As a consequence, efficient strategies for the synthesis of *N* α-chiral allylic indoles are still rare. Recent advances were achieved upon installation of an electron-withdrawing substituent at C2 or C3 positions, which tempers the nucleophilicity at C3 and increases the acidity of the N–H bond^22^. In addition, a two-step protocol by allylation/oxidation of indolines could avoid C3 selectivity issue (Fig. 1c)^24^.

Potentially, chiral *N*^1^-allylic aryl hydrazine could give access to various chiral *N*-allylic indoles by employing a well-established Fischer indole synthesis^27^,^28^,^29^,^30^. This method would allow flexible construction of complex chiral *N*-allylic indoles starting from commercially accessible materials (ketones and aldehydes). Challenge towards the synthesis of chiral *N*^1^-allylic aryl hydrazines arises from the selectivity control: (1) *N*^1^ and *N*^2^ selectivity of aryl hydrazines^31^,^32^,^33^; (2) branched and linear selectivity of the allylic moiety; (3) enantioselectivity of the branched regioisomer. To address these issues, we envisioned that a transition metal-catalysed asymmetric *N*^1^-selective coupling of aryl hydrazines with terminal allenes^34^,^35^,^36^,^37^,^38^,^39^,^40^ could lead to the synthesis of chiral *N*^1^-allylic aryl hydrazines. The *N*^1^ and *N*^2^ selectivities at the aryl hydrazine may differentiate in the oxidative addition step, in which the more acidic N–H bond at *N*^1^ proceeds faster than the less acidic N–H bond at *N*^2^. Furthermore, combination of a suitable transition metal catalyst and a chiral ligand may allow to control branched selectivity and enantioselectivity.

Herein we report a rhodium-catalysed regio- and enantioselective coupling of aryl hydrazines with terminal allenes, which lead to the asymmetric synthesis of *N*-allylic indoles by following a Fischer indolization (Fig. 1c).

## Results

### Reaction optimization of aryl hydrazine allylation

To evaluate our assumption, our studies began with the coupling reaction of phenyl hydrazine and cyclohexylallene in the presence of [Rh(COD)Cl]_2_ (1.25 mmol%) and DPEphos (5.0 mmol%) in 1,2-dichloroethane at 80 °C. Surprisingly, the desired *N*^1^-selective branched product was isolated with a promising 77% yield as a single regioisomer (Table 1, entry 1). Encouraged by the high *N*^1^ and branched regioselectivities, we then tested a range of chiral bidentate phosphine ligands (Table 1, entries 2–10). The ligands Josiphos L and (*R*,*R*)-Diop led to low yield or poor enantioselectivity (Table 1, entries 2 and 3). After extensive screening (see Supplementary Table 1), we were pleased to observe that biaryl-type bisphosphine ligands led to high yield and promising enantiomeric excess (*ee*) (Table 1, entries 4–6). Increasing the steric effect of the Segphos-type ligand could significantly increase the enantioselectivity. The best *ee* was obtained with a bulky (*S*)-DTBM-Segphos ligand (Table 1, entries 6–8). Similarly, (*R*)-DTBM-Binap gave a comparable result (Table 1, entry 9). The enantiomeric purity could be enriched by a single recrystallization of the toluene sulfonic acid salt. Control experiments indicated that both rhodium catalyst and ligand are necessary for the coupling reaction of aryl hydrazine with allene to proceed (Table 1, entries 10 and 11).

### Substrates scope of aryl hydrazine allylation

**Figure 2:**
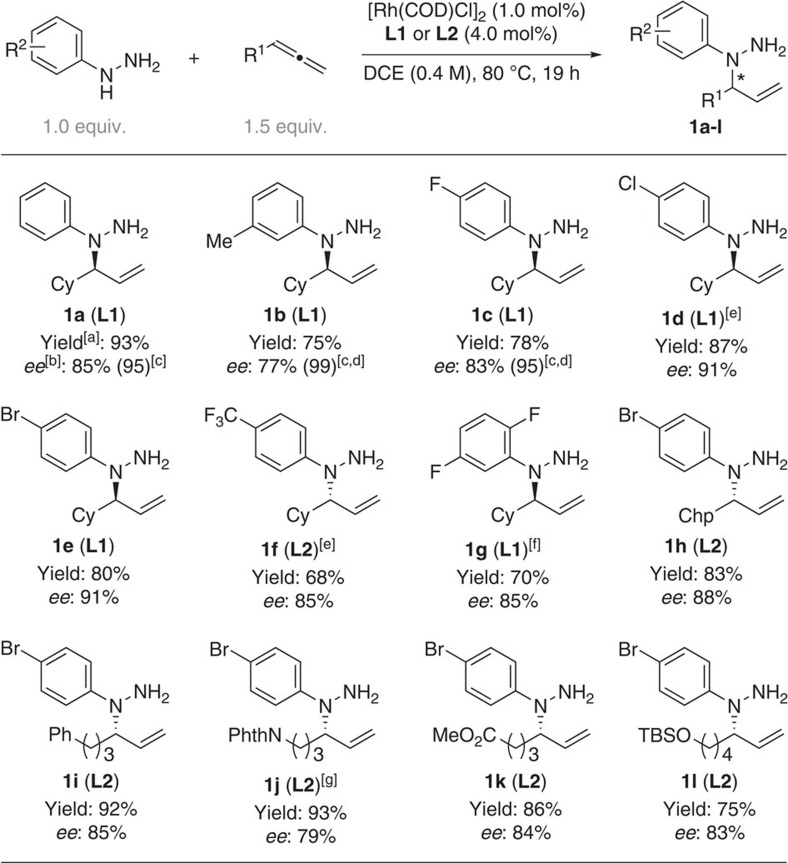
Scope of Rh-catalysed coupling of aryl hydrazines with allene. ^[a]^Isolated yield. ^[b]^Determined by chiral HPLC. ^[c]^*ee* after recrystallization from tosylic acid salt. ^[d]^Reaction in 1.0 mmol scale. ^[e]^[Rh(cod)Cl]_2_ (2 mol%), **L1** or **L2** (8.0 mol%). ^[f]^Reaction at 100 °C. ^[g]^The NMR spectrum of **1j** is not fully pure due to contamination of acetone (formation of hydrazone) during the purification. This problem can be avoided in the process of one-pot asymmetric synthesis of *N*-allylic indoles. Chp, cycloheptyl; HPLC, high-performance liquid chromatography; Phth, phaloyl; TBS, *tert*-butylsilyl.

With the optimized conditions in hand, we then examined the scope of the addition of different aryl hydrazines with terminal allenes (Fig. 2). Various aryl hydrazines were coupled with cyclohexylallene in up to 93% isolated yield (**1a**) and up to 91% *ee* (**1d**–**e**). Practically, the *N*-allylated aryl hydrazines can be recrystallized from the corresponding tosylic acid salts to enrich the enantiomeric excess (**1a**–**c**). Several mono-substituted allenes were also tested (**1h**–**l**). Allenes bearing a phthaloyl-protected amine, an ester function and a silylether were suitable (**1g**–**l**).

### One-pot asymmetric synthesis of *N*-allylic indoles

**Figure 3:**
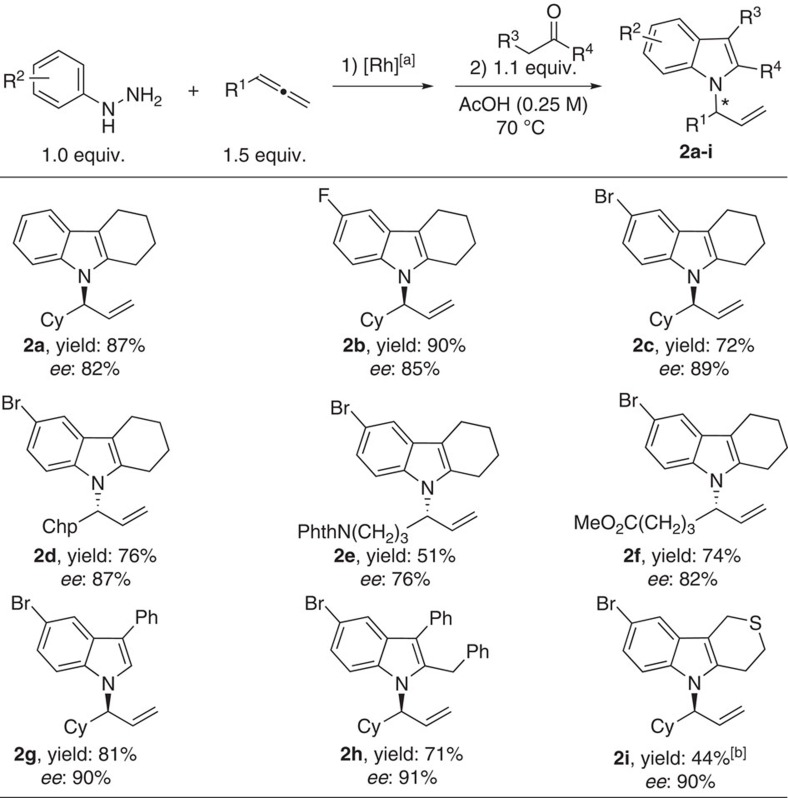
Scope of one-pot asymmetric synthesis of ***N***-allylic indoles. ^[a]^Scope conditions of Fig. 2. ^[b]^Reaction carried out at 100 °C.

To investigate the compatibility of our strategy in the synthesis of *N*-allylic indoles via a one-pot process, the crude reaction mixture of the coupling step (**1a**) was subjected directly for the Fischer indolization with cyclohexanone in acetic acid at 70 °C. The desired *N*-allylic indole **2a** was obtained in 87% isolated yield over two steps with retention of the enantiomeric purity. Variation with other aryl hydrazines and allenes using this one-pot process led to the synthesis of the corresponding *N*-allylic indoles in up to 90% yield and up to 91% *ee* (**2b**–**f**). Furthermore, aldehydes, phenyl-substituted ketones as well as a dihydro-2*H*-thiopyran-4(3*H*)-one were well tolerated under standard conditions (Fig. 3).

**Figure 4:**
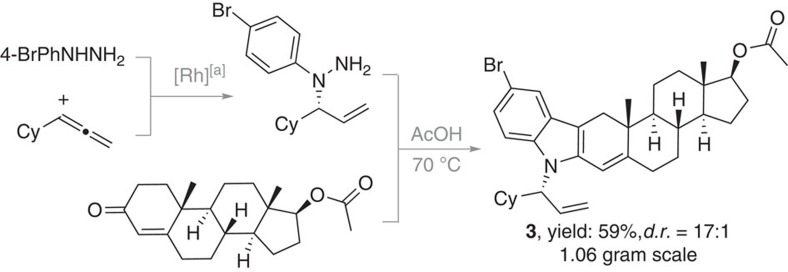
One-pot late-stage indolization of (+)-testosterone acetate. ^[a]^[Rh(cod)Cl]_2_ (1.0 mol%), **L2** (4.0 mol%), 1,2-dichloroethane (0.4 M), 80 °C, 19 h.

To test the scalability and application for the synthesis of bioactive molecules, we applied the one-pot process for the late-stage indolization of (+)-testosterone acetate. To our delight, the desired indole product **3** was obtained in 59% yield and 17:1 diastereoselectivity in 1.06 gram scale, which indicates the practicality and usefulness of the method (Fig. 4).

### Mechanistic investigations

**Figure 5:**
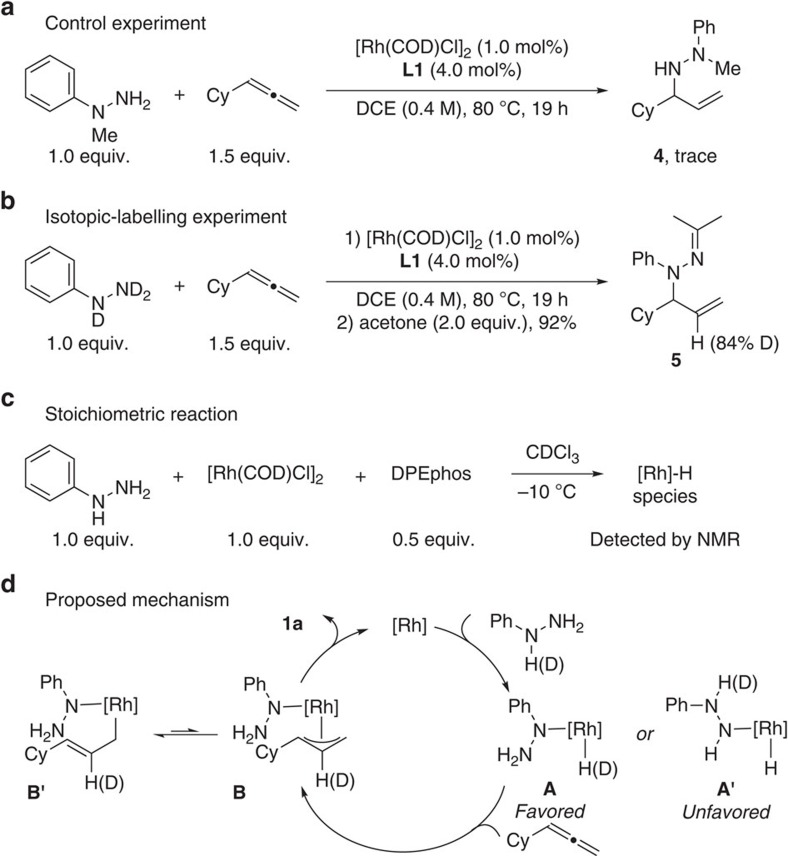
Mechanistic investigations. (**a**) Control experiment with 1-methyl-1-phenylhydrazine. (**b**) Isotopic-labelling experiment with [D_3_]phenylhydrazine. (**c**) Stoichiometric reaction of phenylhydrazine with catalysts. (**d**) Proposed mechanism.

To probe the possible reaction mechanism, a control experiment of 1-methyl-1-phenylhydrazine with cyclohexylallene was conducted under optimized conditions (Fig. 5a). The reaction was sluggish and gave only traces of the *N*^2^-allylated product **4**, which is in accord with the lower reactivity of *N*^2^ of the aryl hydrazine. Deuterium-labelling experiments with [D_3_]phenylhydrazine under optimized conditions displayed that deuterium was only incorporated in the internal position of the double bond (Fig. 5b). Stoichiometric reaction of phenylhydrazine with [{Rh(COD)Cl}_2_] and DPEphos in CDCl_3_ was monitored by NMR spectroscopy. After 5 min at room temperature, the ^1^H NMR spectrum (263 K) showed a major rhodium hydride species at *δ*=−15.4 p.p.m. (^1^*J*_Rh-H_=14 Hz), which is indicative of the oxidative addition of the N–H bond to rhodium (Fig. 5c).

On the basis of these observations, the following mechanism can be proposed (Fig. 5d). Oxidative addition of the phenyl hydrazine to Rh(I) generates Rh(III) complex (**A** or **A′**)^41^. The oxidative addition step favours the formation of intermediate **A** because of the higher acidity of N–H bond of *N*^1^ than *N*^2^. Hydrometalation of the less-substituted double bond could generate π-allyl-Rh (or δ-allyl-Rh) complex **B** (or **B′**)^42^,^43^,^44^,^45^, which could generate the desired branched *N*-allylic aryl hydrazine **1a** via reductive elimination. The *N*-selectivity was determined within the oxidative addition step^46^.

## Discussion

We have developed the enantioselective *N*-selective coupling of aryl hydrazines with allenes via a rhodium(I)/DTBM-Segphos or rhodium(I)/DTBM-Binap catalyst system, which allowed the asymmetric synthesis of various valuable *N*-allylic indoles by following a one-pot Fischer indolization. *N*-selective allylation of aryl hydrazines using alkynes, target-oriented synthesis, and mechanistic investigations are currently underway in our laboratory and will be reported in due course.

## Methods

### Allylation of aryl hydrazines

To a screw-cap Schlenk tube was added [Rh(cod)Cl]_2_ (0.005 mmol, 1 mol%), **L1** or **L2** (0.02 mmol, 4 mol%), aryl hydrazine (0.5 mmol, 1.0 equiv.), 1,2-dichloroethane (0.4 M) and allene (0.75 mmol, 1.5 equiv.). The Schlenk tube was sealed and the mixture was stirred for 19 h at 80 °C (or 100 °C). After cooling to room temperature, the solvent was removed by rotary evaporation. The crude product was purified by flash column chromatography to obtain the corresponding allylic hydrazine.

### One-pot asymmetric synthesis of *N*-allylic indoles

To the reaction mixture of allylation of hydrazine was added ketone or aldehyde (0.55 mmol, 1.1 equiv.), and the mixture was stirred for half hour to form the corresponding hydrazine, then solvent was removed under reduced pressure. To the residue was added acetic acid (2.0 ml, 0.25 M), and the reaction mixture was stirred for 3–18 h at 70 °C (or 100 °C). The volatiles were removed by rotary evaporation and the crude reaction mixture was purified by flash column chromatography. The *ee* of each product was determined by HPLC analysis using chiral stationary phases. All new compounds were fully characterized. For NMR, high resolution mass spectrometry (HRMS) analysis and HPLC traces of the compounds in this article, see Supplementary Figs 1–53. General information, materials, synthesis and characterization of compounds in this article (**1a**–**l**, **2a**–**i**, **3**, **4** and **5**), and experimental part for mechanistic investigations see Supplementary Methods.

## Additional information

**How to cite this article:** Xu, K. *et al.* Asymmetric synthesis of *N*-allylic indoles via regio- and enantioselective allylation of aryl hydrazines. *Nat. Commun.* 6:7616 doi: 10.1038/ncomms8616 (2015).

## Supplementary Material

Supplementary Information Supplementary Figures 1-53, Supplementary Table 1, Supplementary Methods and Supplementary References

## Figures and Tables

**Figure 1:**
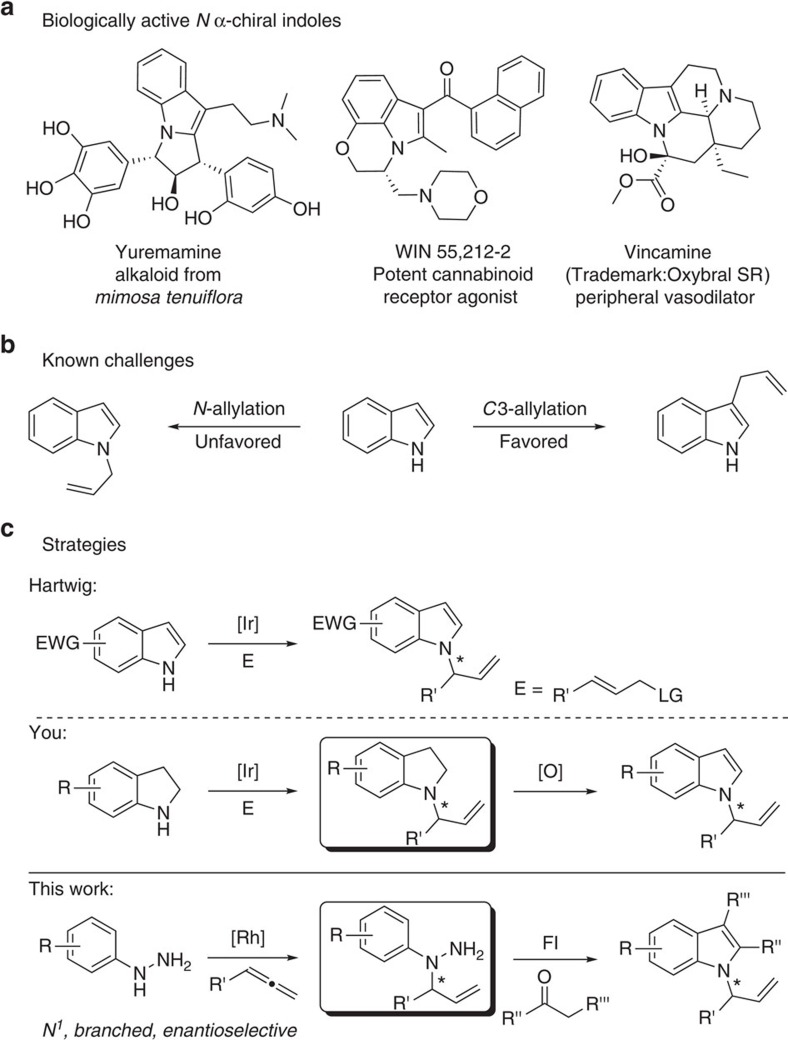
Challenges for asymmetric synthesis of *N*-allylic indoles. (**a**) Selected examples of biologically active *N* α-chiral indoles. (**b**) Selectivity issue for allylation of indoles. (**c**) Strategies for transition metal-catalysed asymmetric synthesis of *N*-allylic indoles. LG, leaving group; EWG, electron-withdrawing group; FI, Fischer indolization.

**Table 1:** Optimization of Rh-catalyzed coupling of phenyl hydrazine with cyclohexylallene.
