# Supplementary material for: Asymmetric synthesis of N-allylic indoles via regio- and enantioselective allylation of aryl hydrazines
Source: Nat Commun. 2015 Jul 3;6:7616. doi: 10.1038/ncomms8616 (PMC4506507; doi:10.1038/ncomms8616)
Supplement: Supplementary Information — Supplementary Figures 1-53, Supplementary Table 1, Supplementary Methods and Supplementary References [file ncomms8616-s1.pdf]

Supplementary Figures

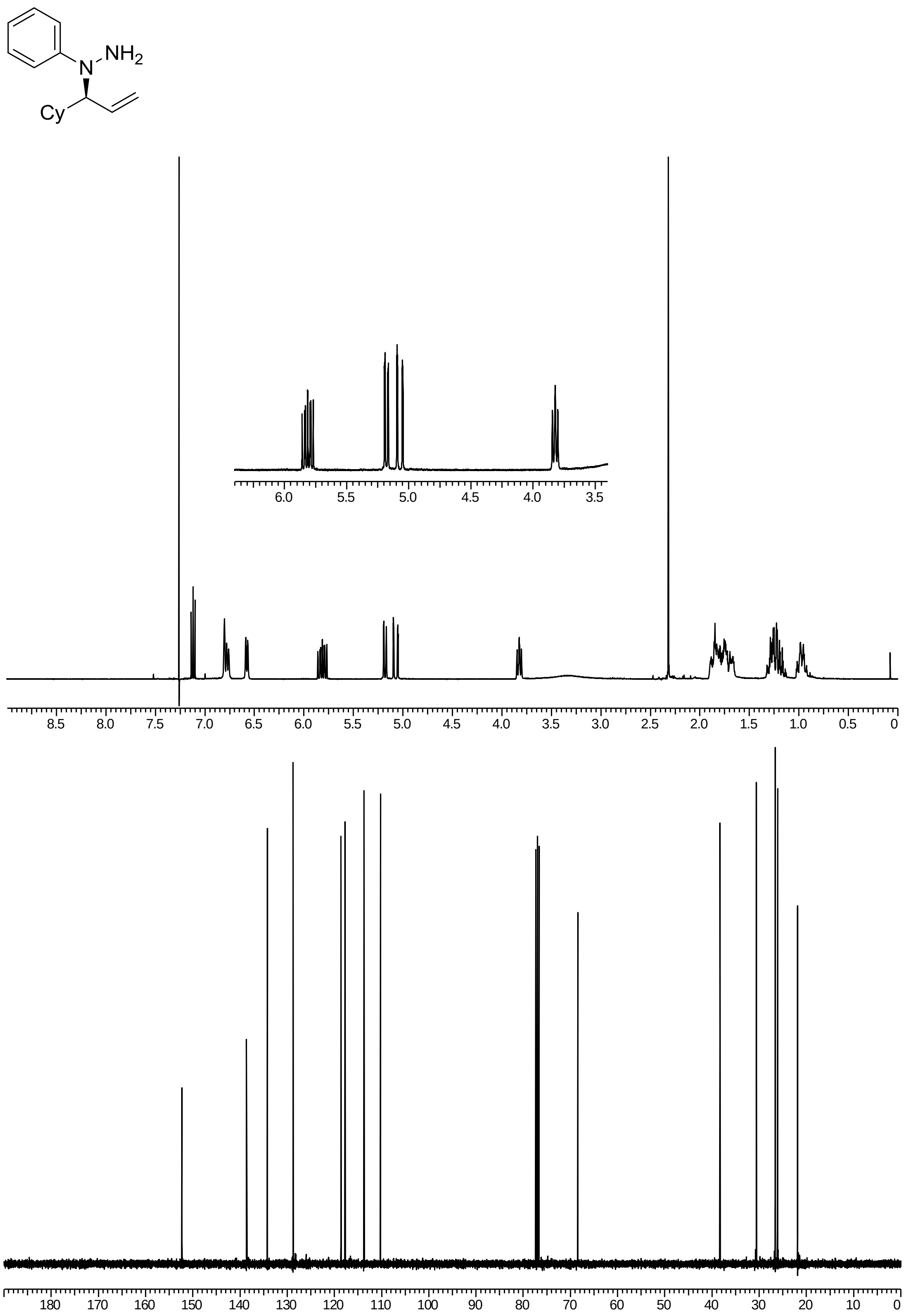

Supplementary figure 1. ^{1}H and ^{13}C NMR spectra for product **1a**

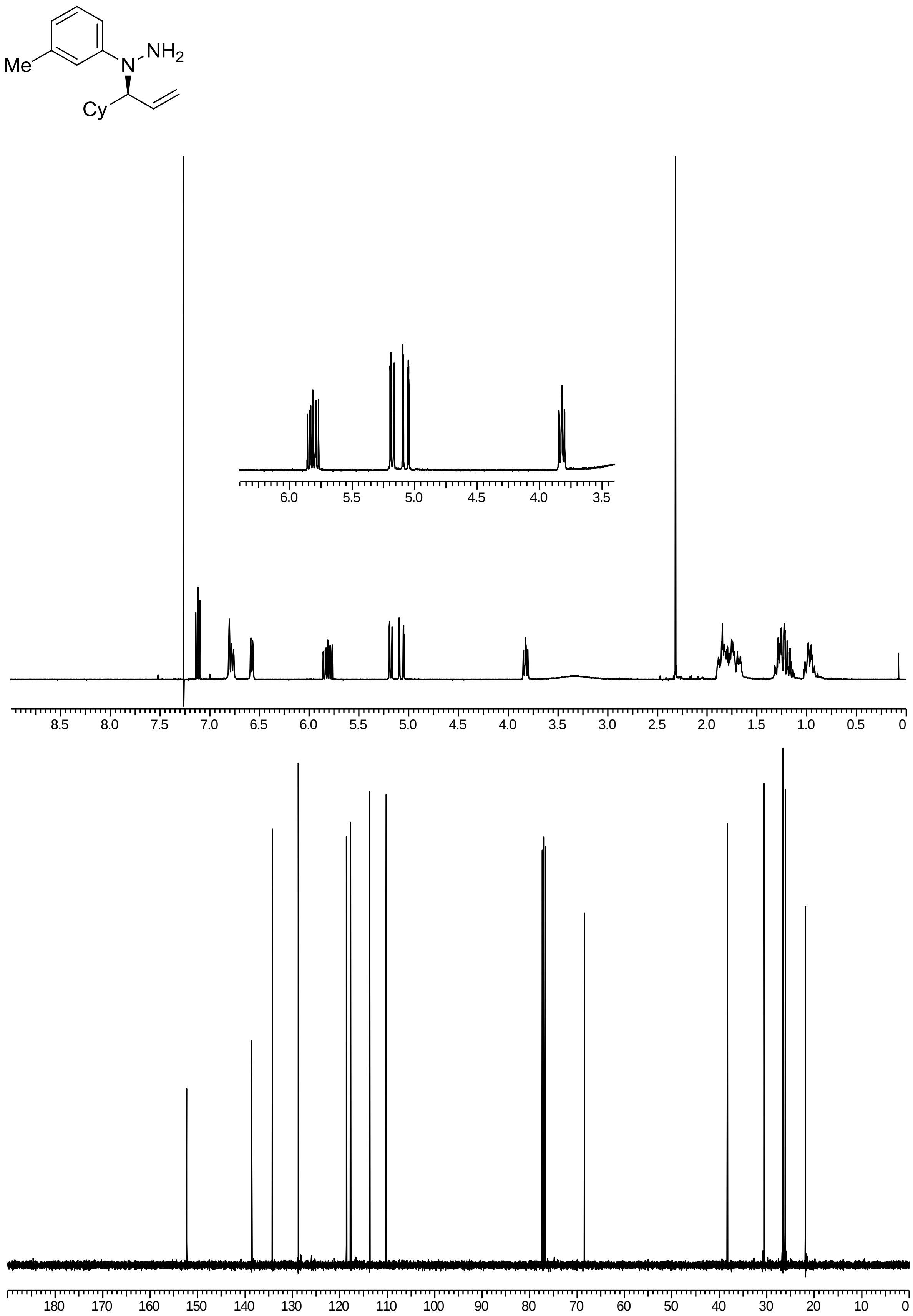

Supplementary figure 2. ^{1}H and ^{13}C NMR spectra for product **1b**

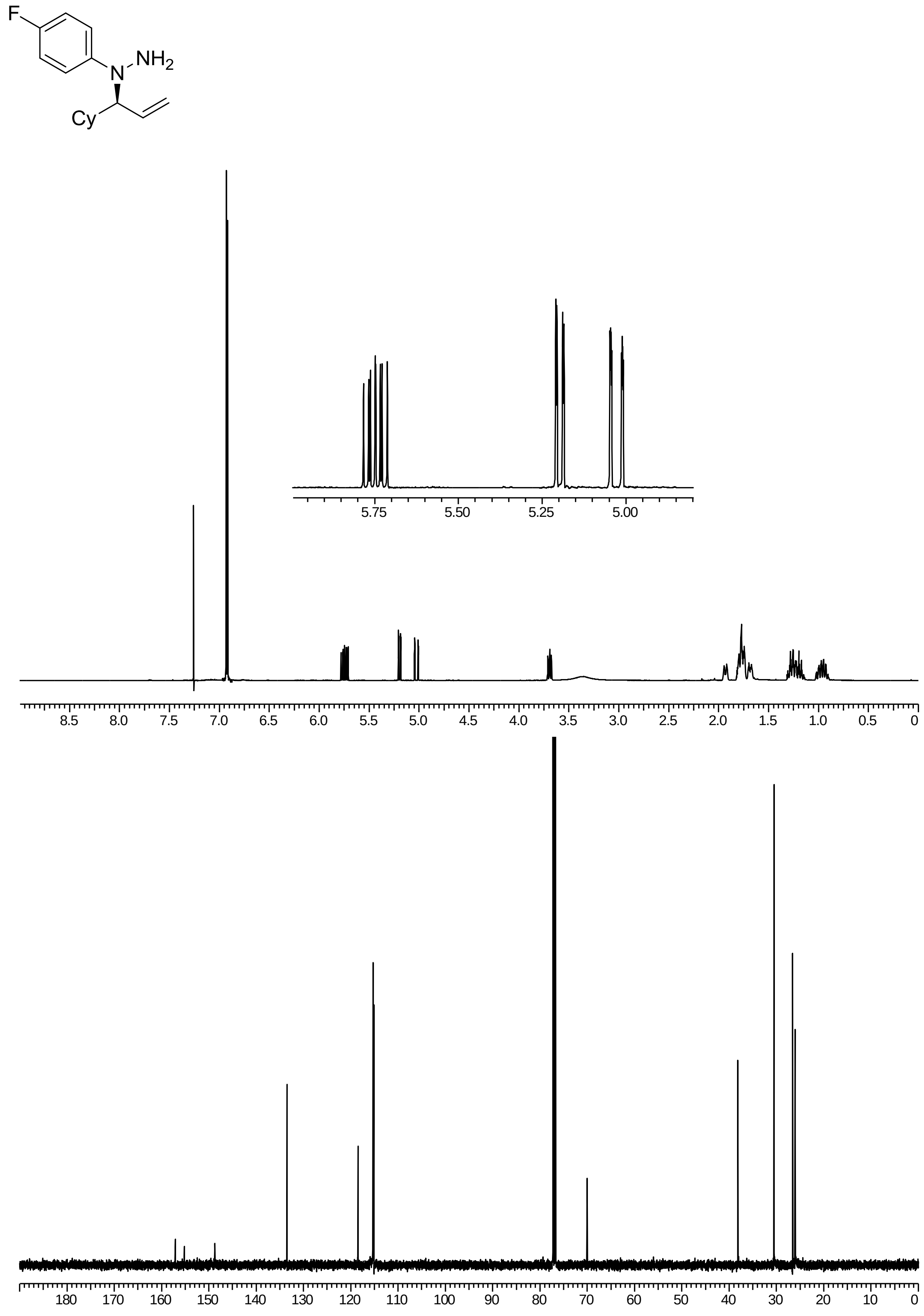

Supplementary figure 3. ^{1}H and ^{13}C NMR spectra for product **1c**

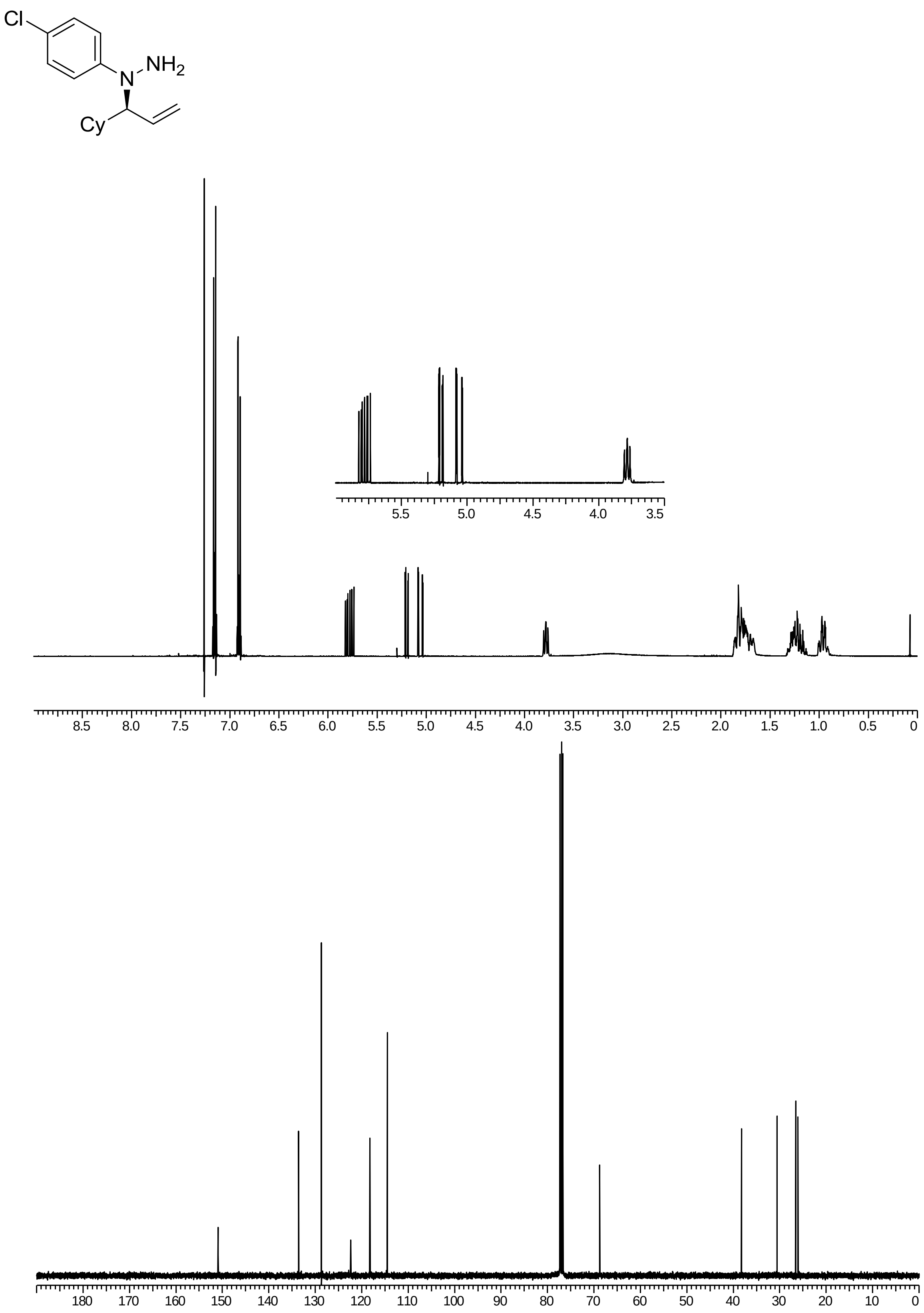

Supplementary figure 4. ^{1}H and ^{13}C NMR spectra for product **1d**

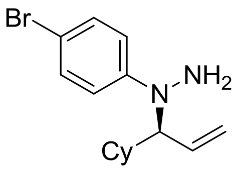

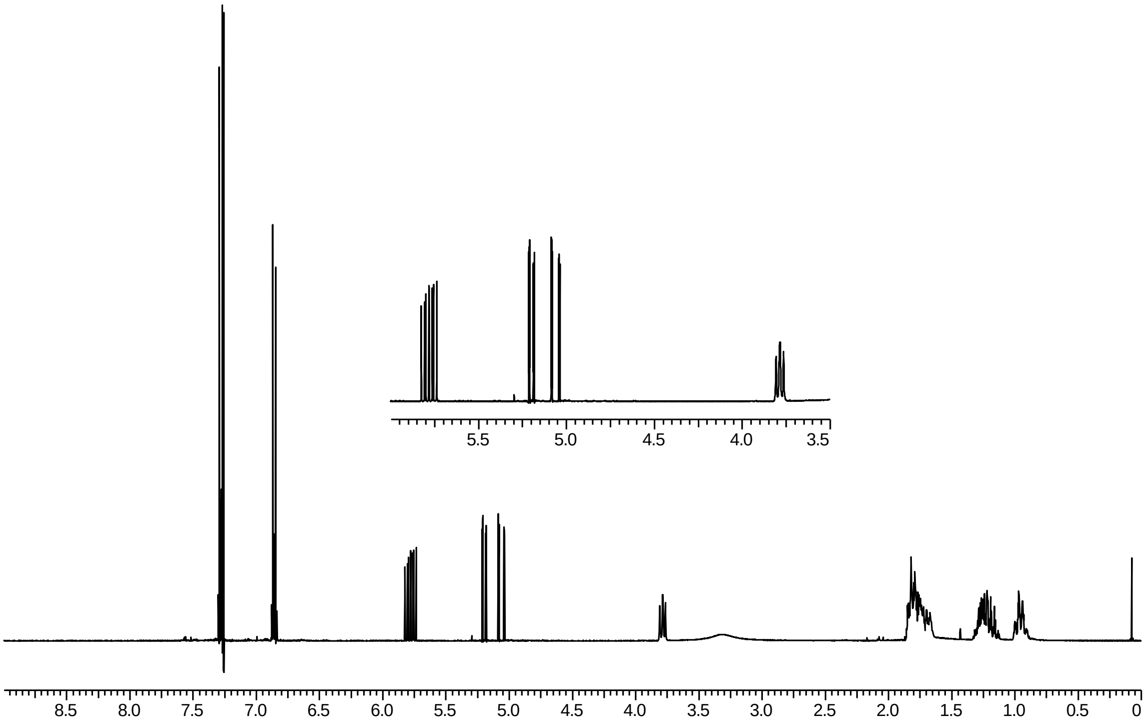

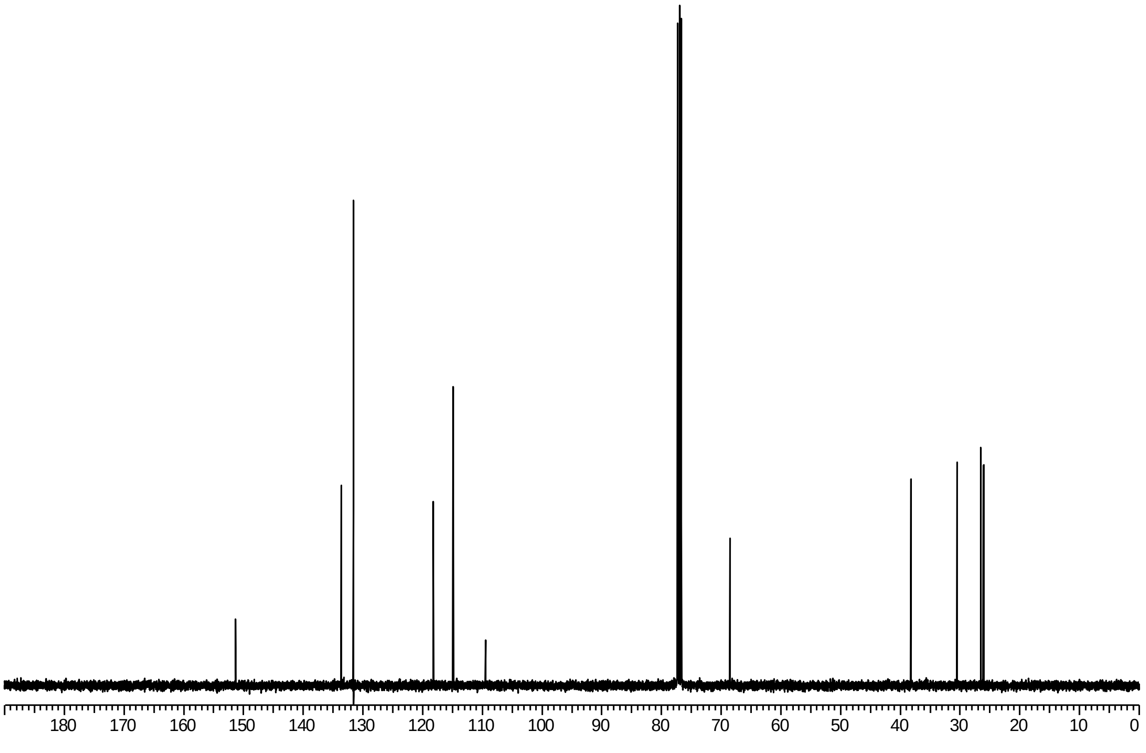

Supplementary figure 5. ^{1}H and ^{13}C NMR spectra for product **1e**

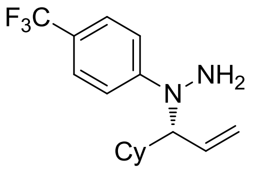

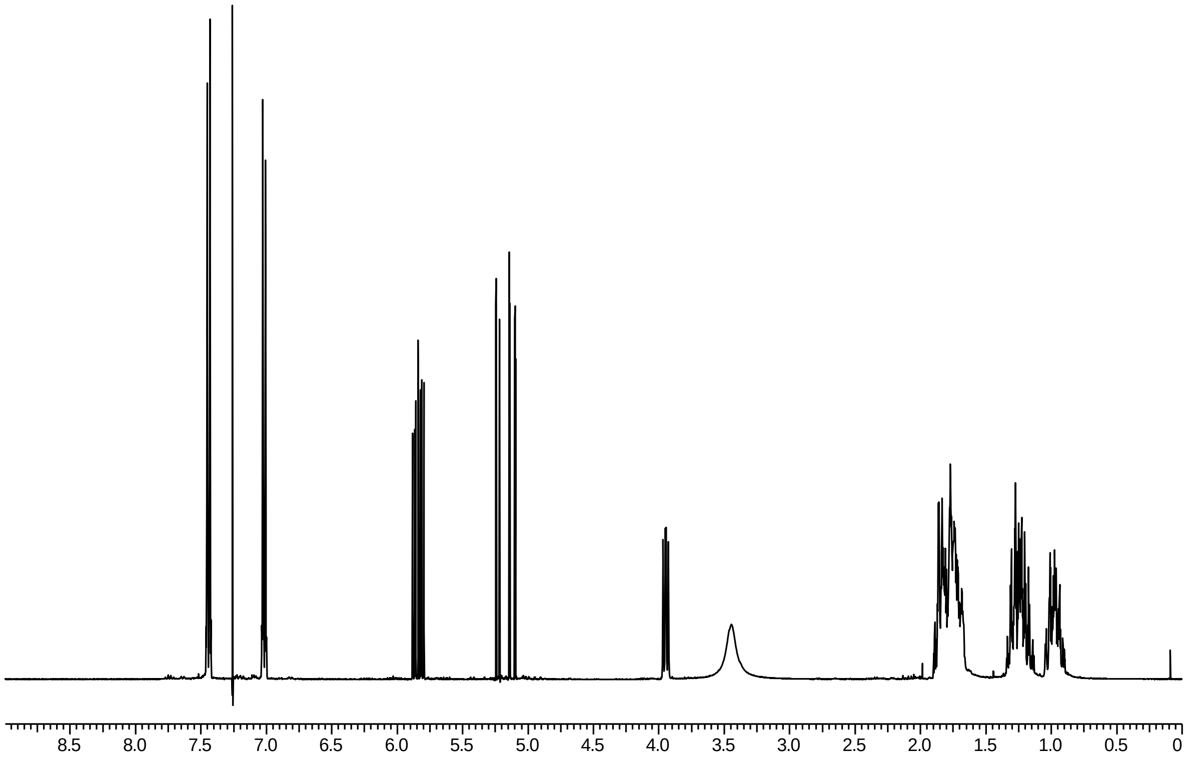

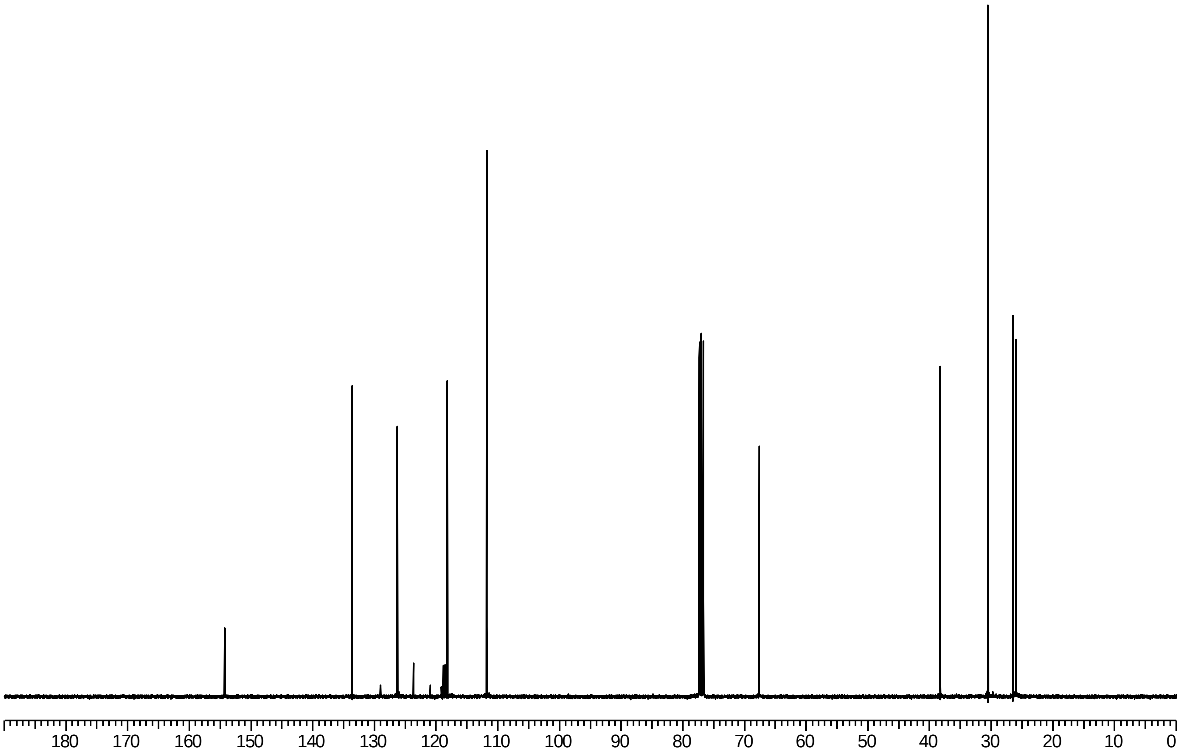

Supplementary figure 6. ^{1}H and ^{13}C NMR spectra for product **1f**

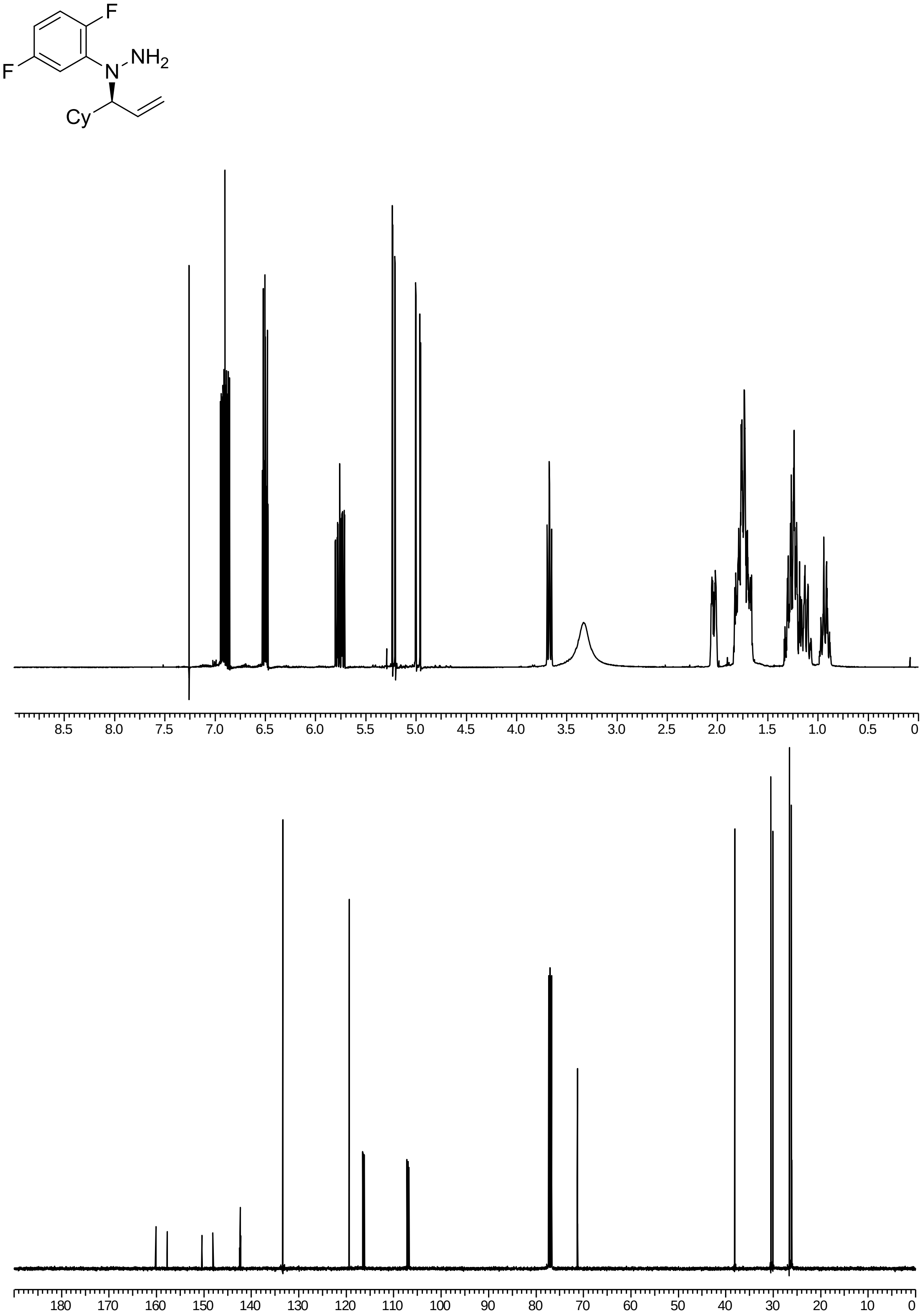

Supplementary figure 7. ^{1}H and ^{13}C NMR spectra for product **1g**

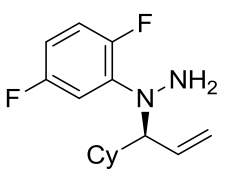

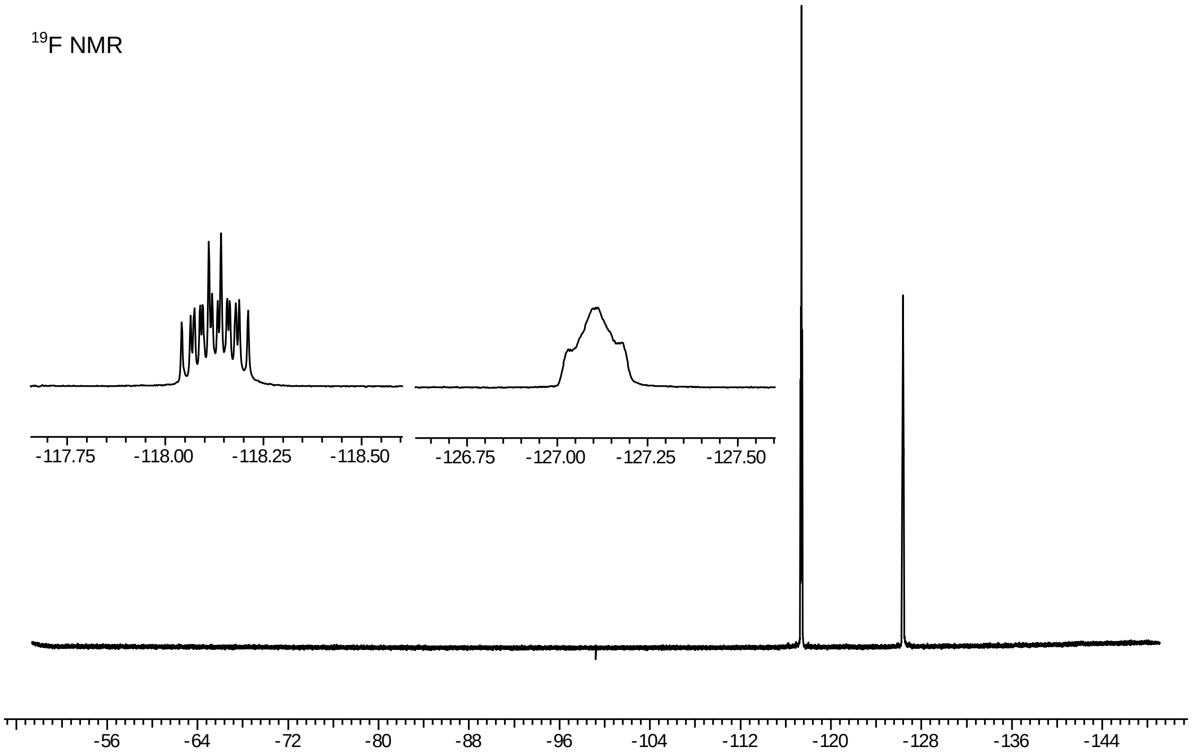

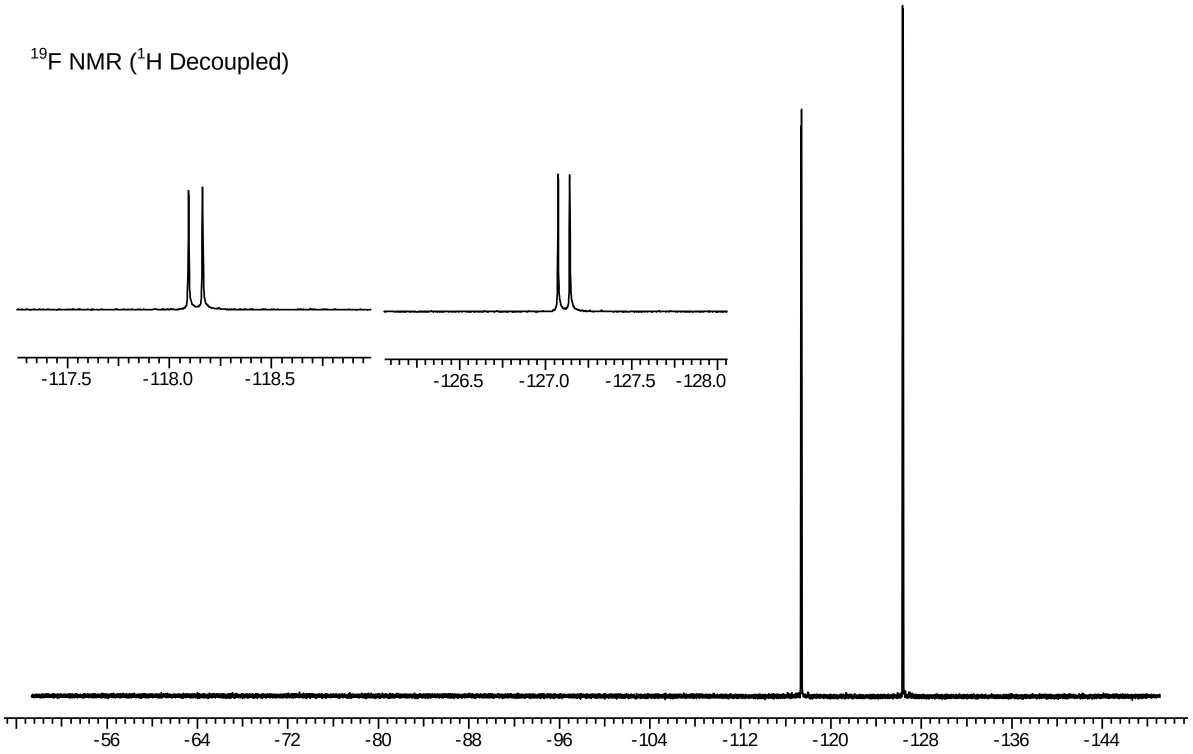

Supplementary figure 8. ^{19}F NMR spectra for product **1g**

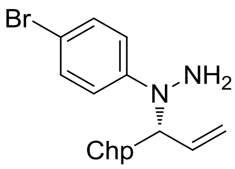

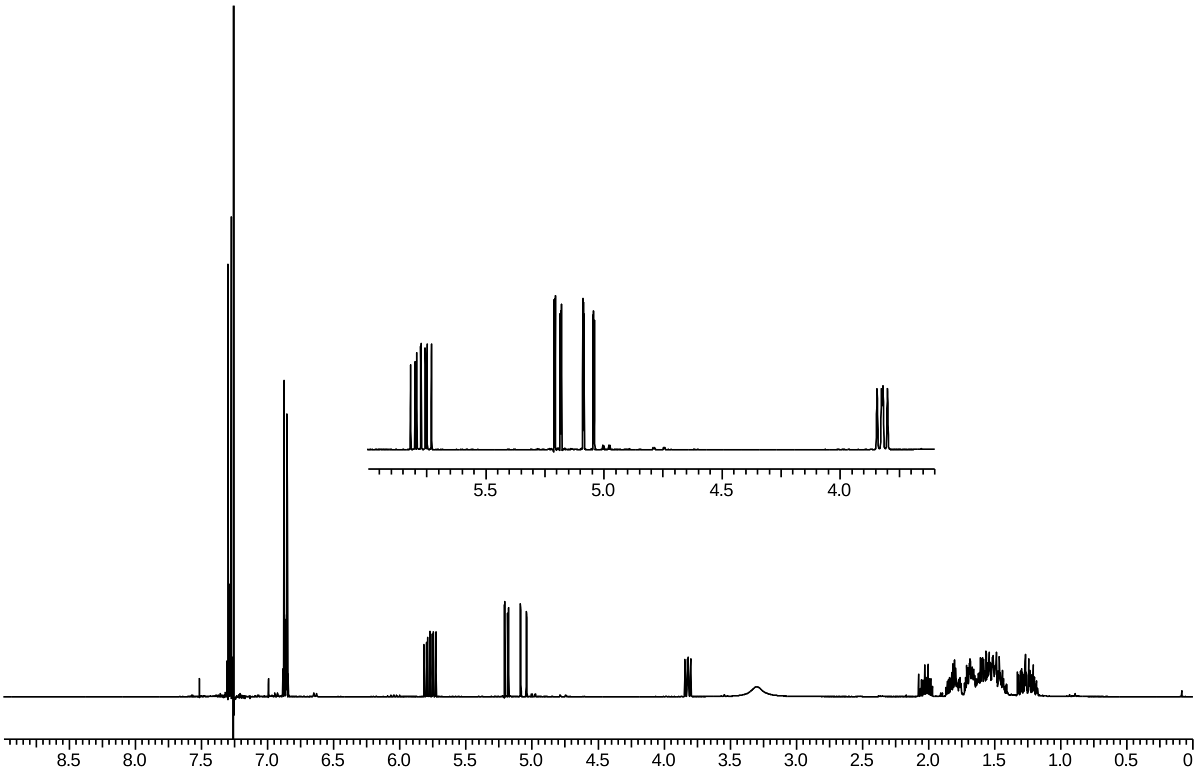

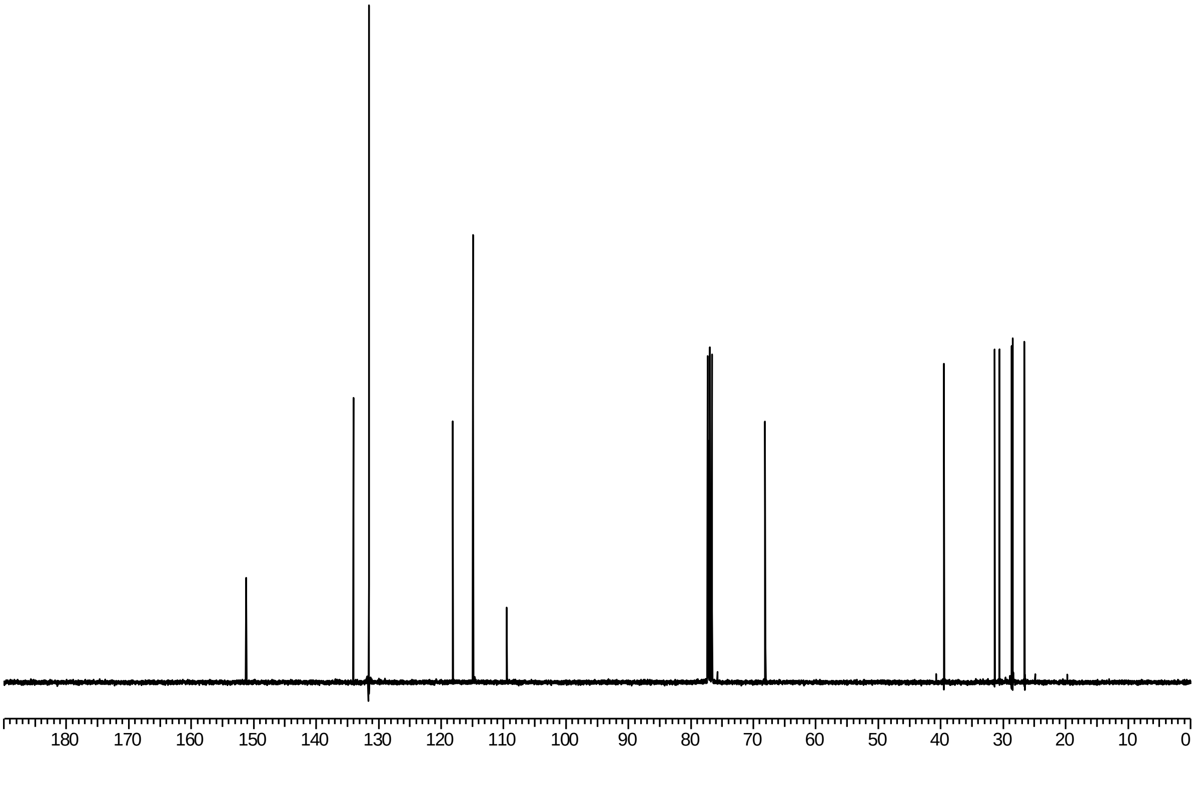

Supplementary figure 9. ^{1}H and ^{13}C NMR spectra for product **1h**

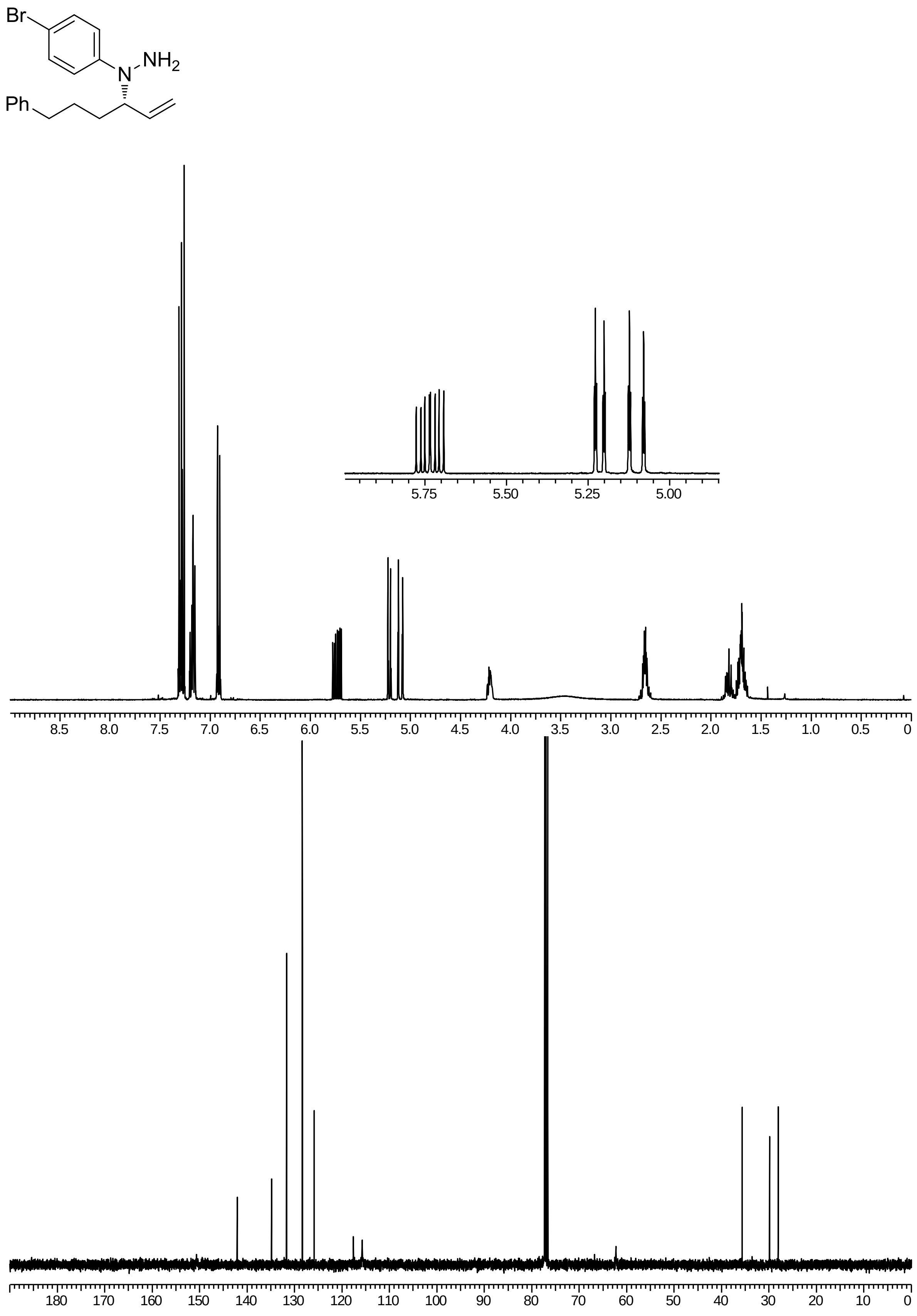

Supplementary figure 10. ^{1}H and ^{13}C NMR spectra for product **1i**

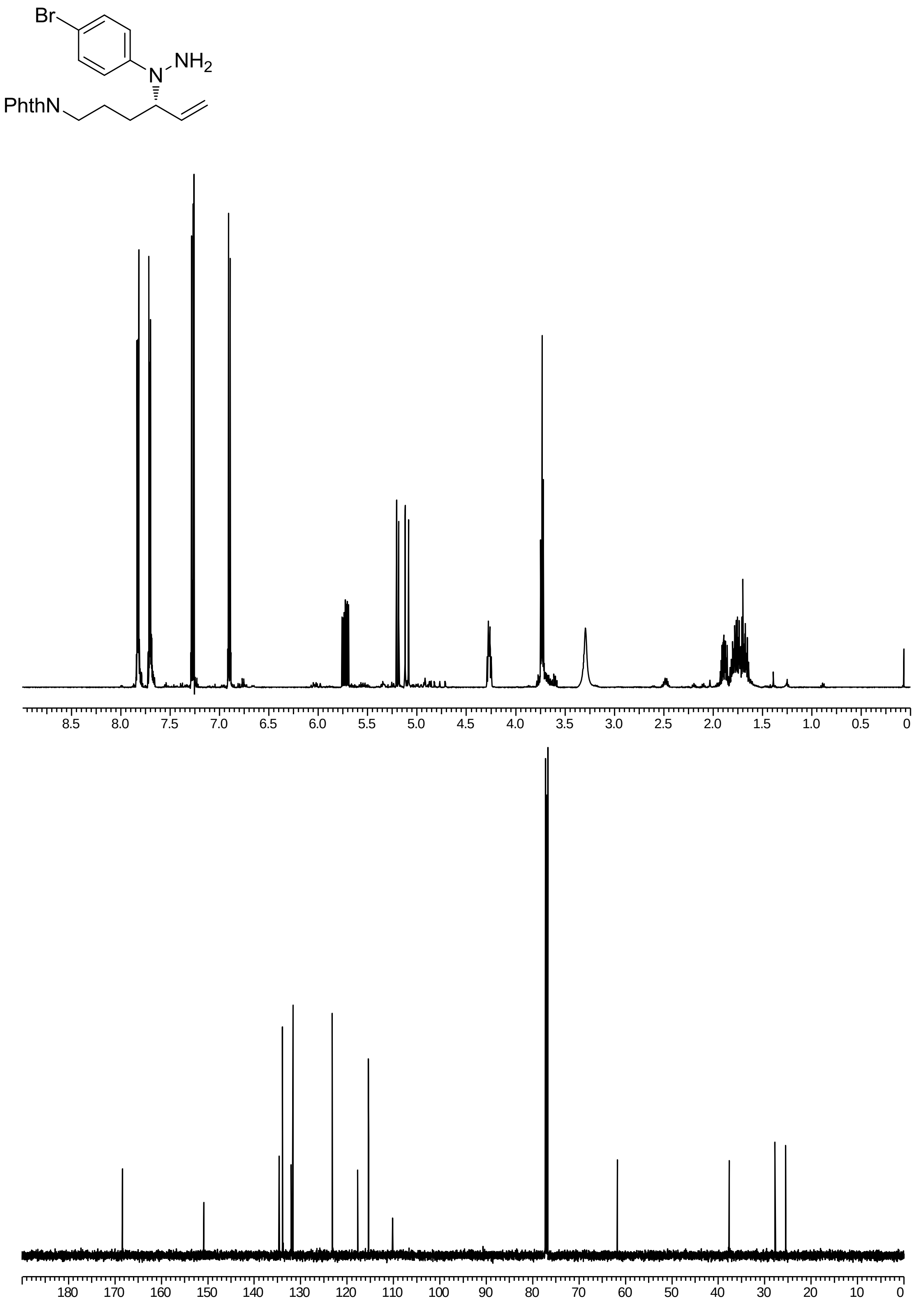

Supplementary figure 11. ^{1}H and ^{13}C NMR spectra for product **1j**

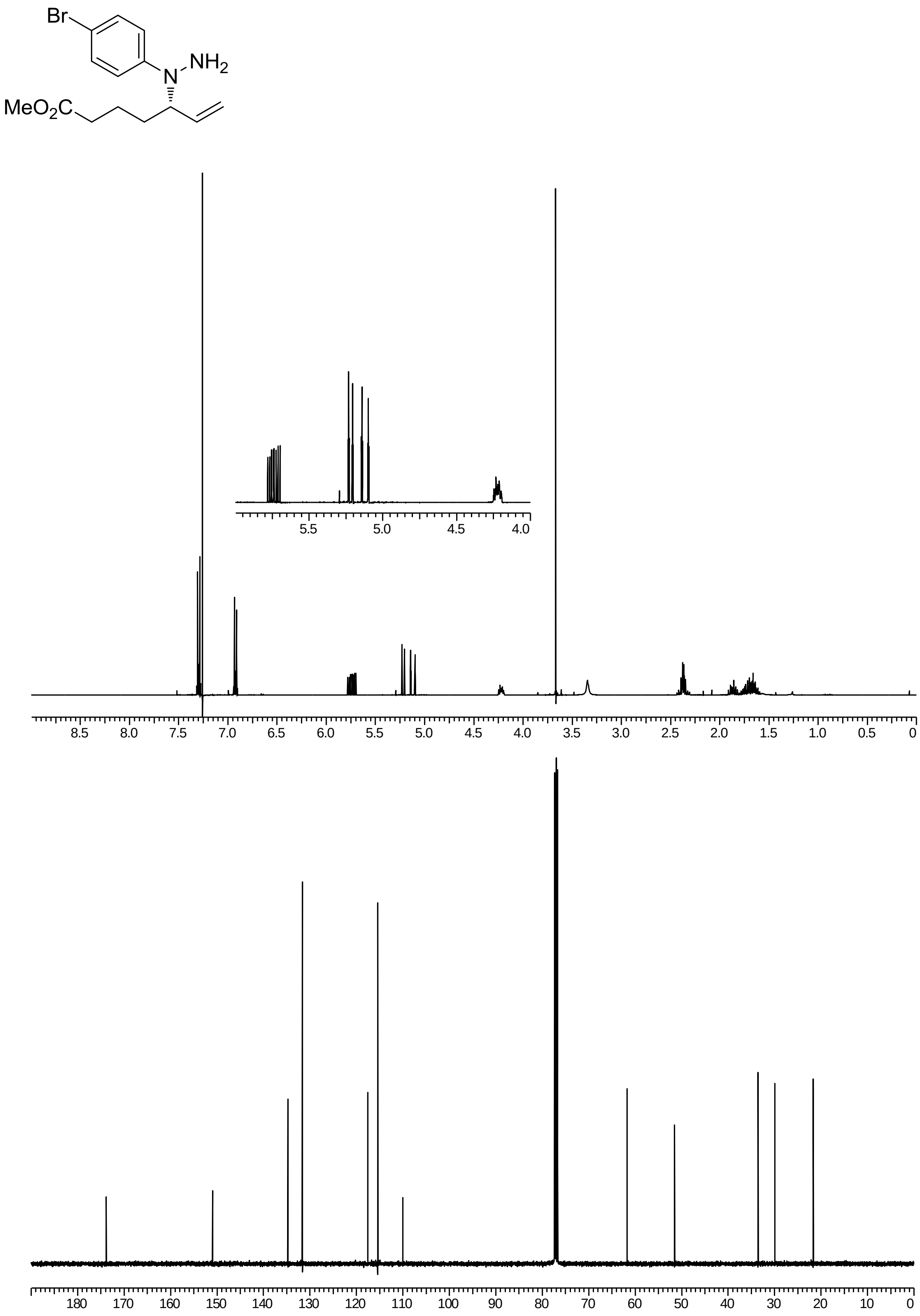

Supplementary figure 12. ^{1}H and ^{13}C NMR spectra for product **1k**

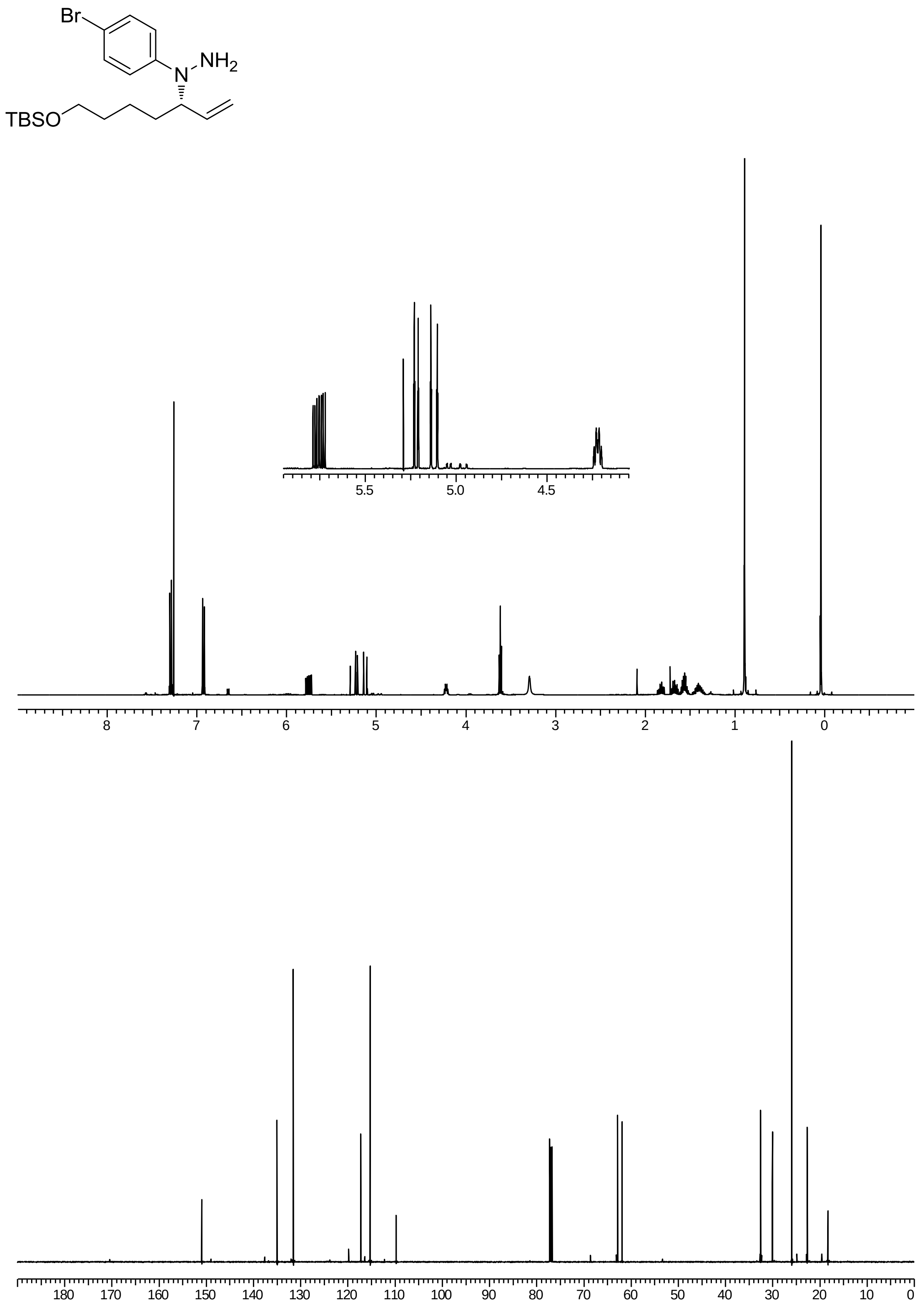

Supplementary figure 13. ^{1}H and ^{13}C NMR spectra for product **1l**

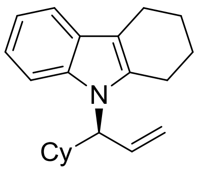

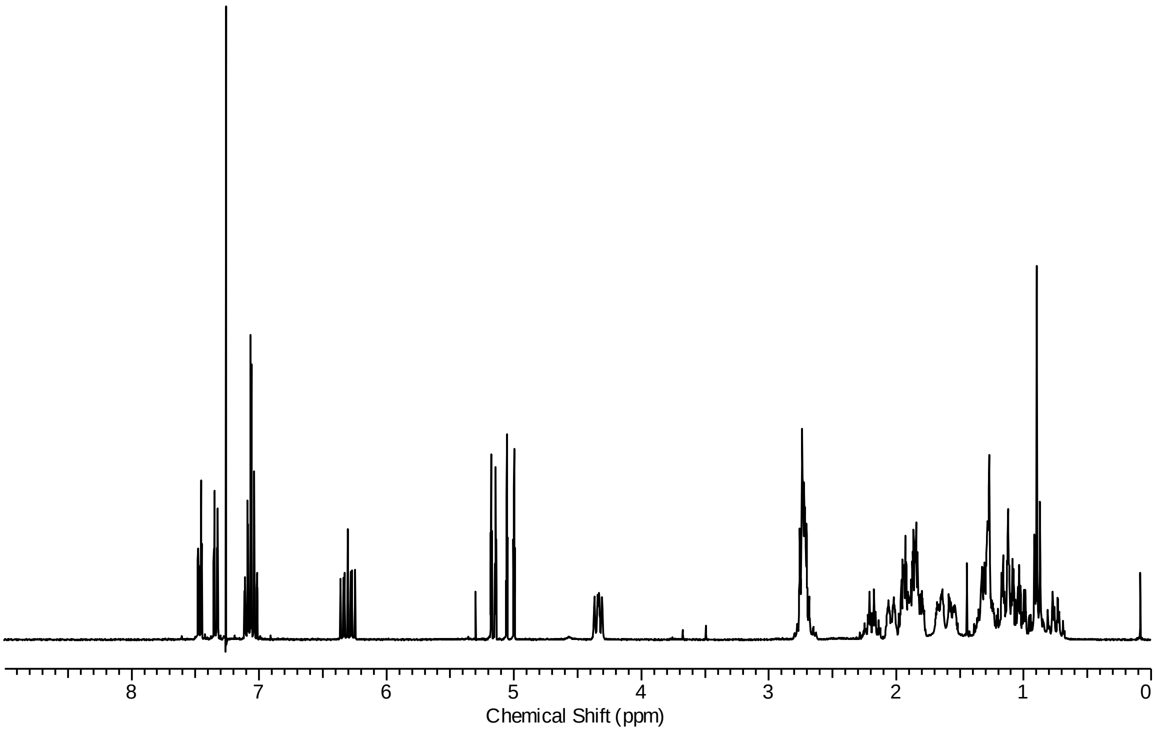

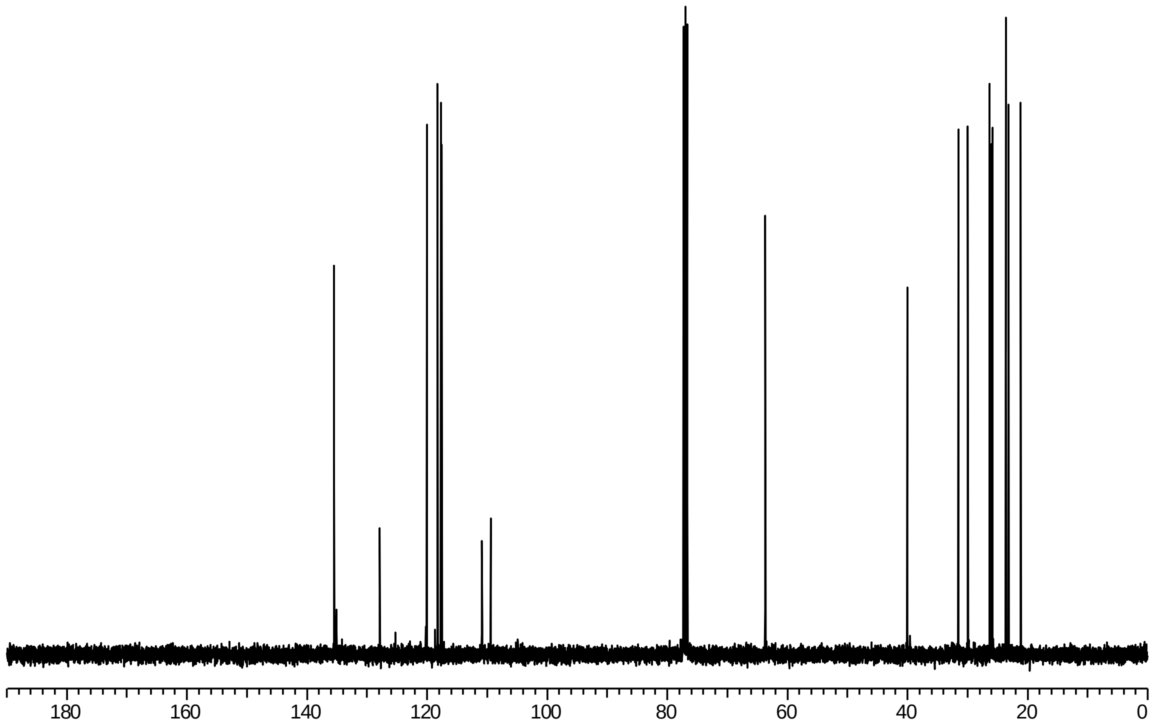

Supplementary figure 14. ^{1}H and ^{13}C NMR spectra for product **2a**

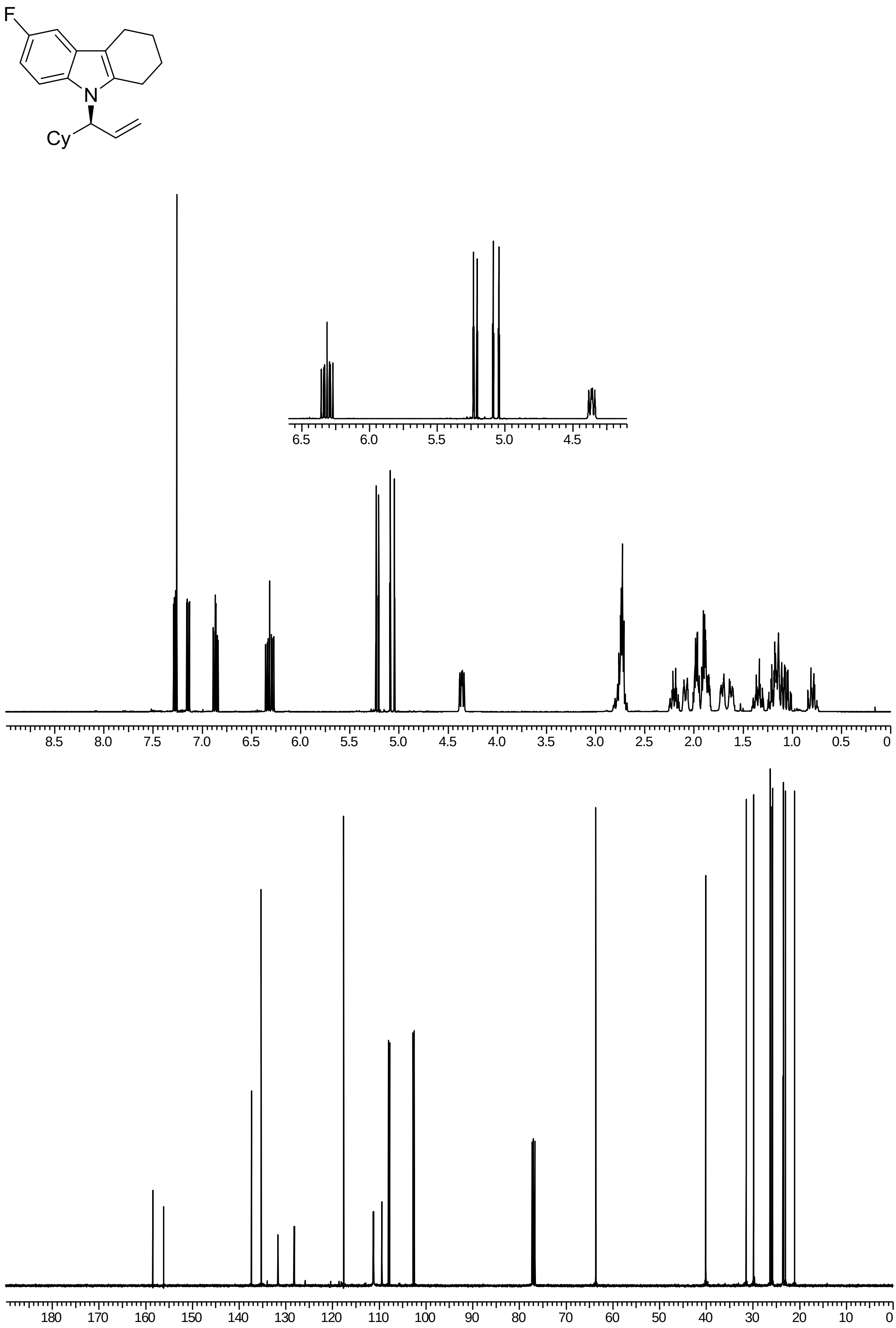

Supplementary figure 15. ^{1}H and ^{13}C NMR spectra for product **2b**

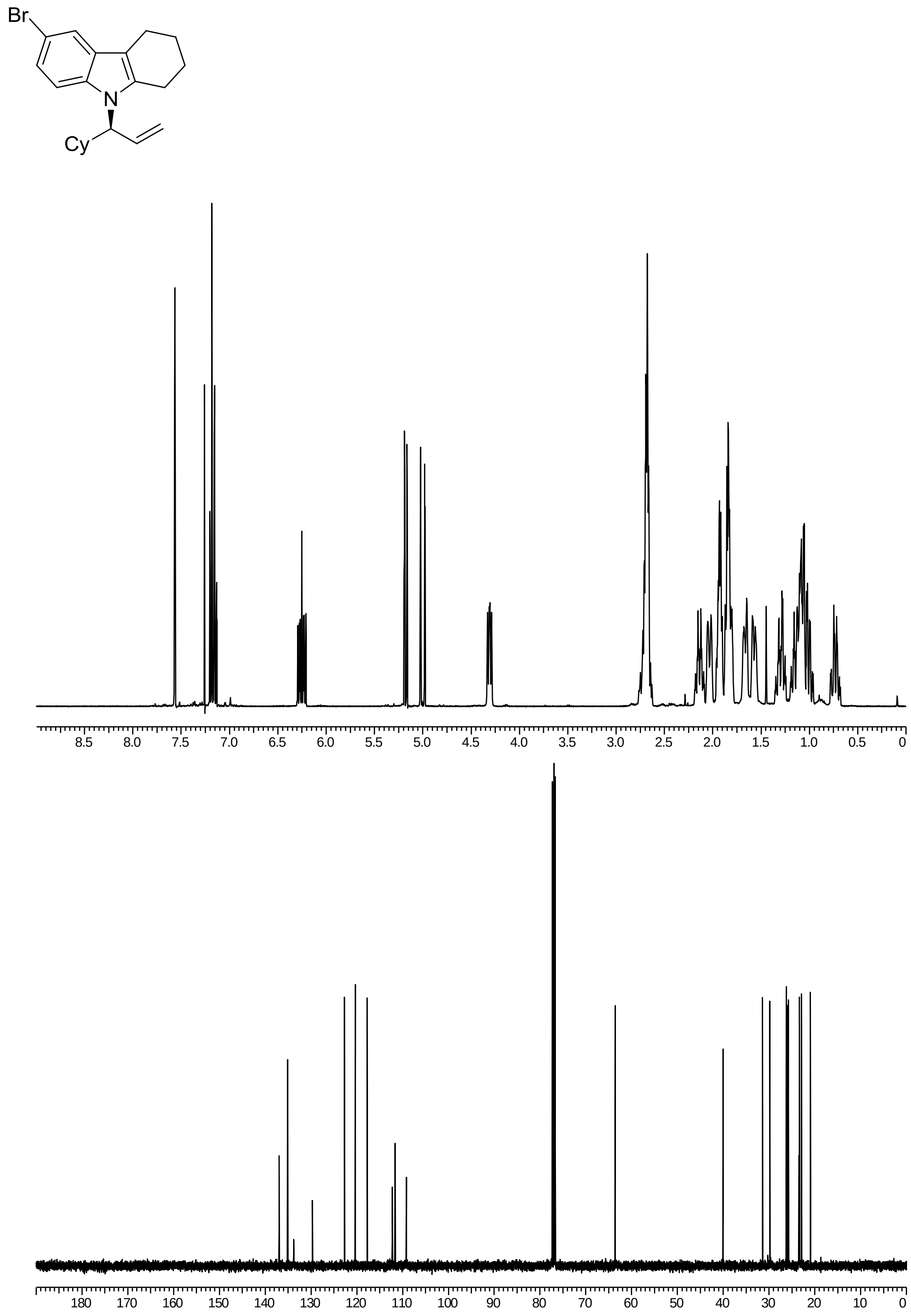

Supplementary figure 16. ^{1}H and ^{13}C NMR spectra for product **2c**

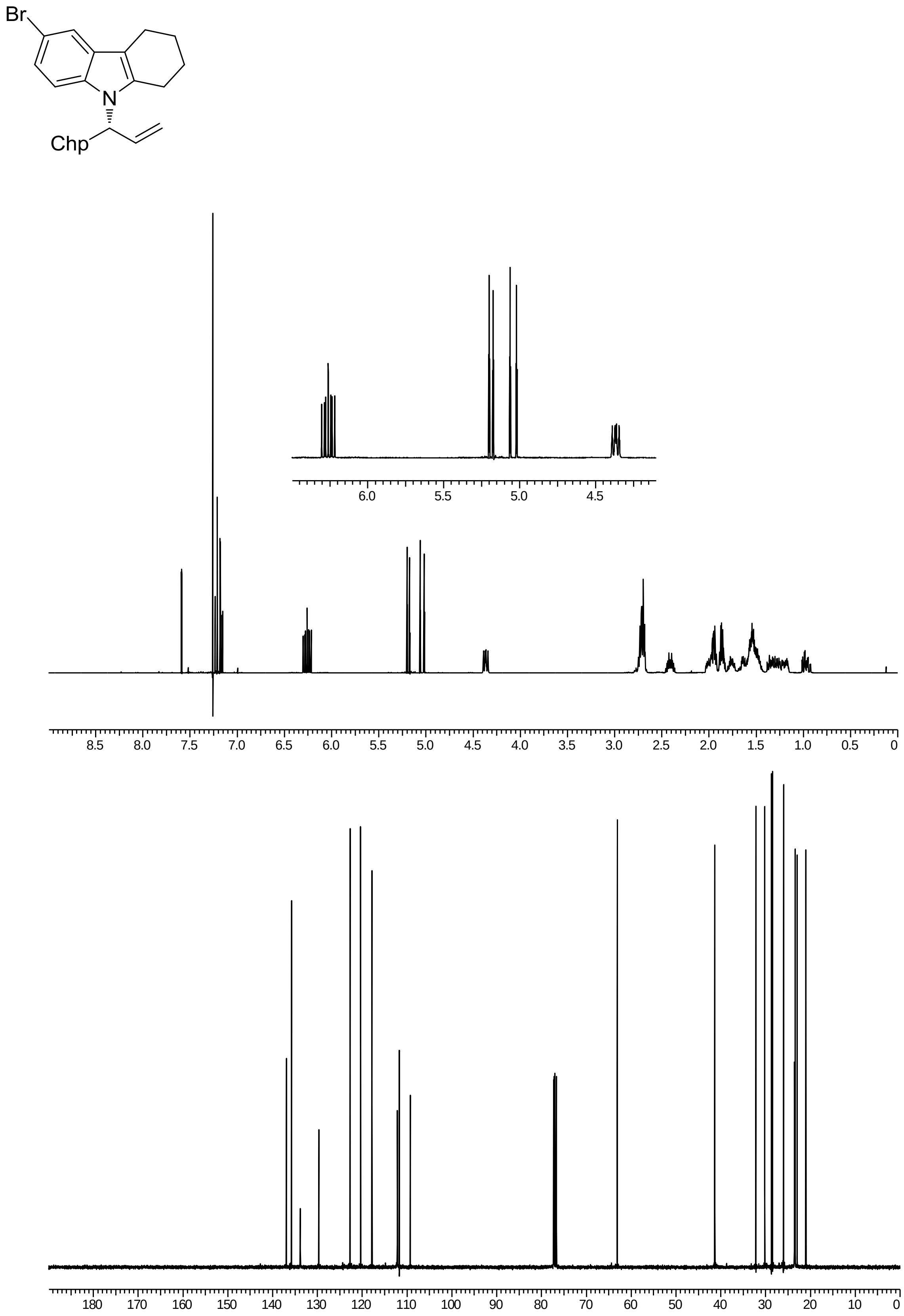

Supplementary figure 16. ^{1}H and ^{13}C NMR spectra for product **2d**

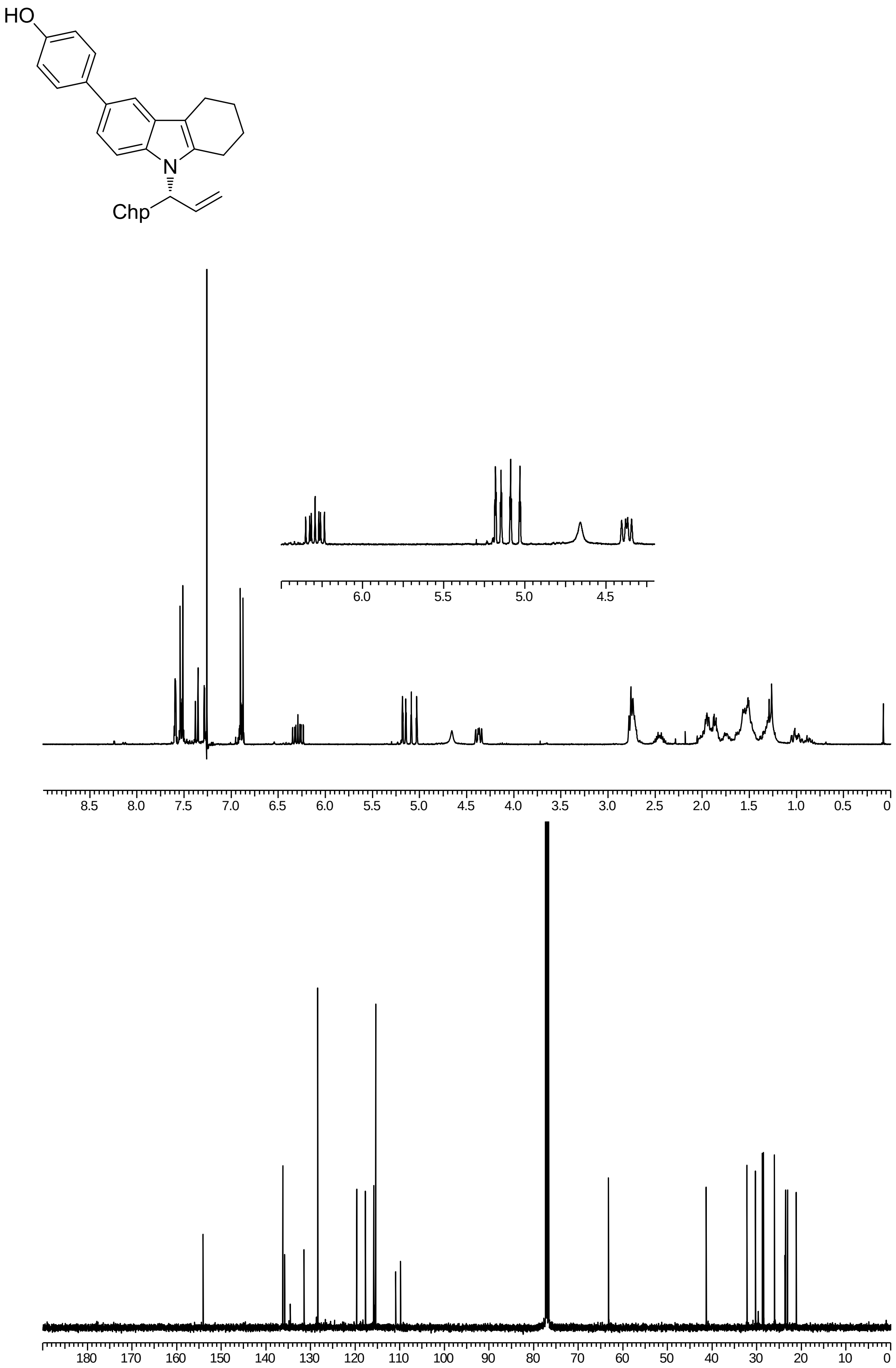

Supplementary figure 18. ^{1}H and ^{13}C NMR spectra for product **2dee**

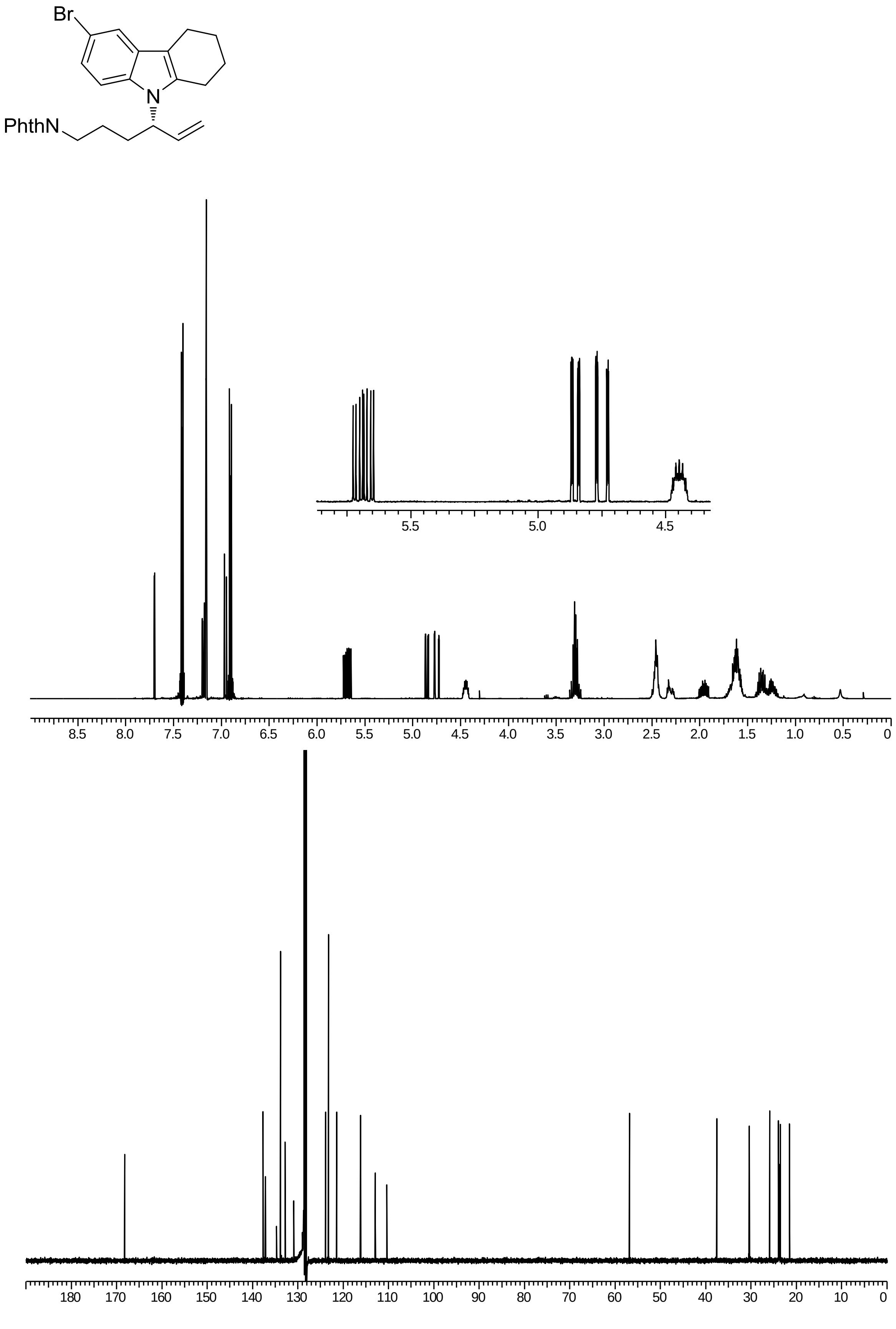

Supplementary figure 19. ^{1}H and ^{13}C NMR spectra for product **2e**

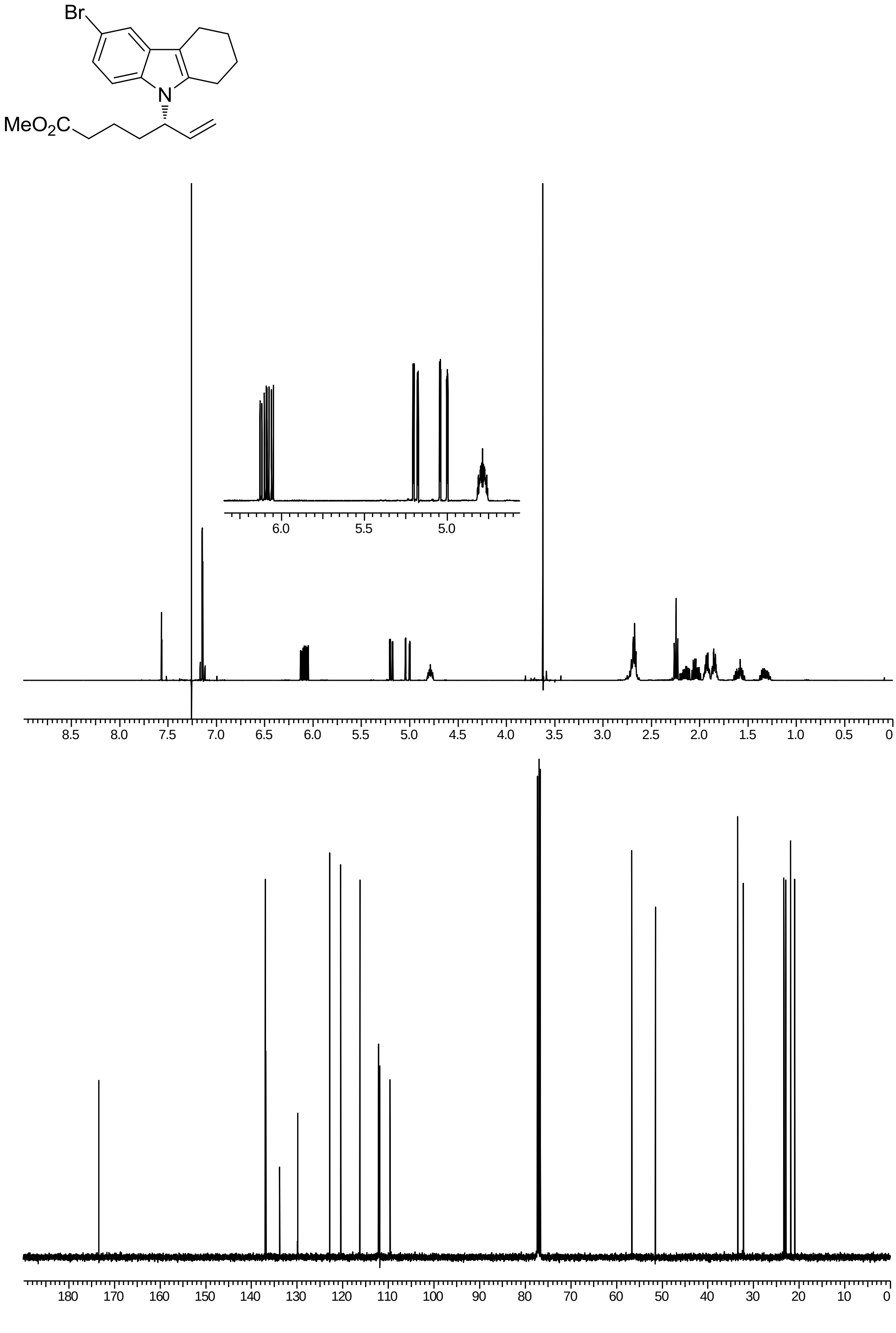

Supplementary figure 20. ^{1}H and ^{13}C NMR spectra for product **2f**

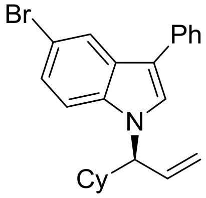

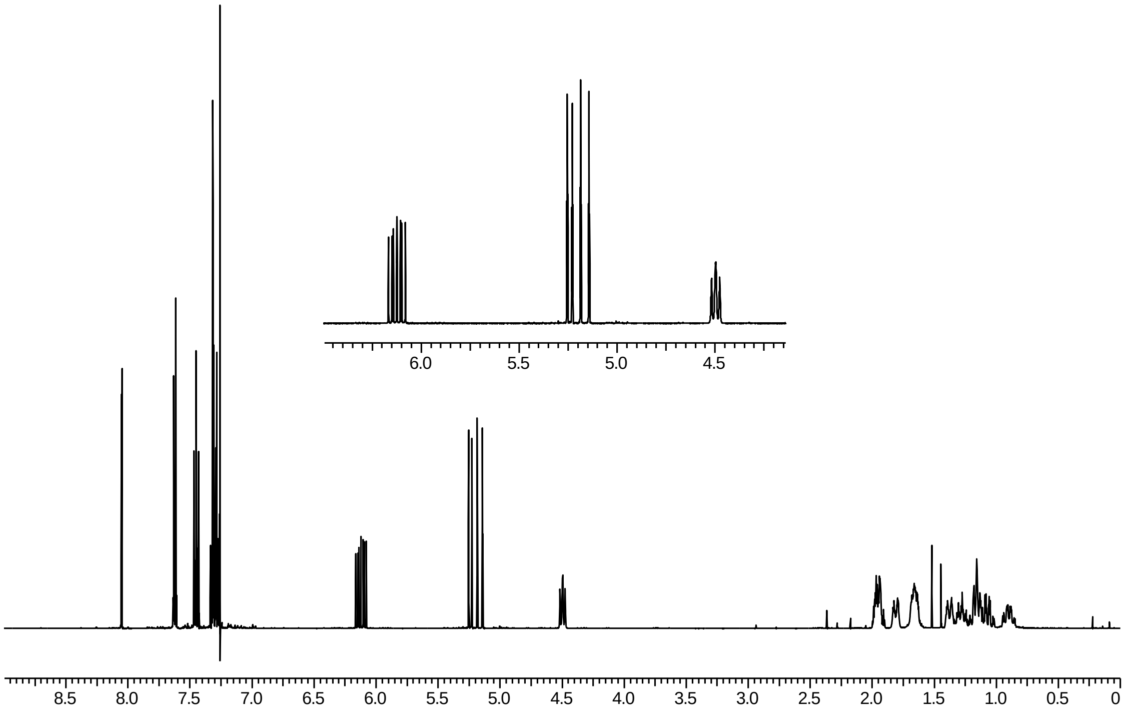

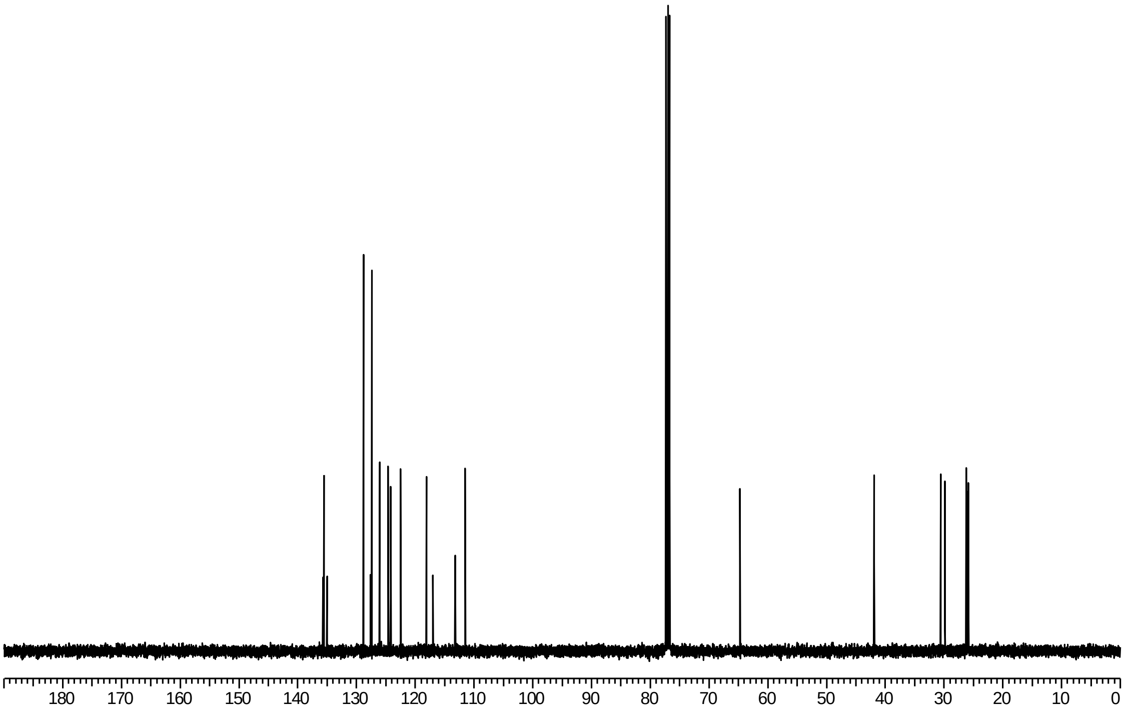

Supplementary figure 21. ^{1}H and ^{13}C NMR spectra for product **2g**

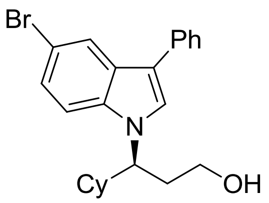

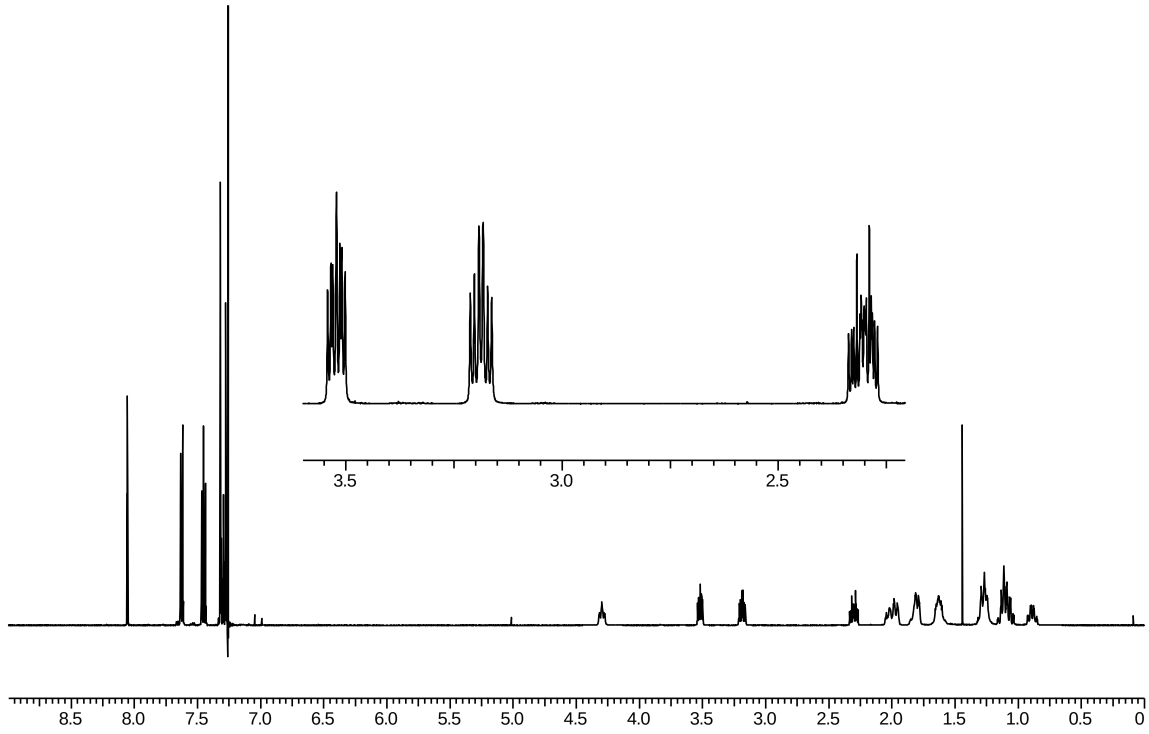

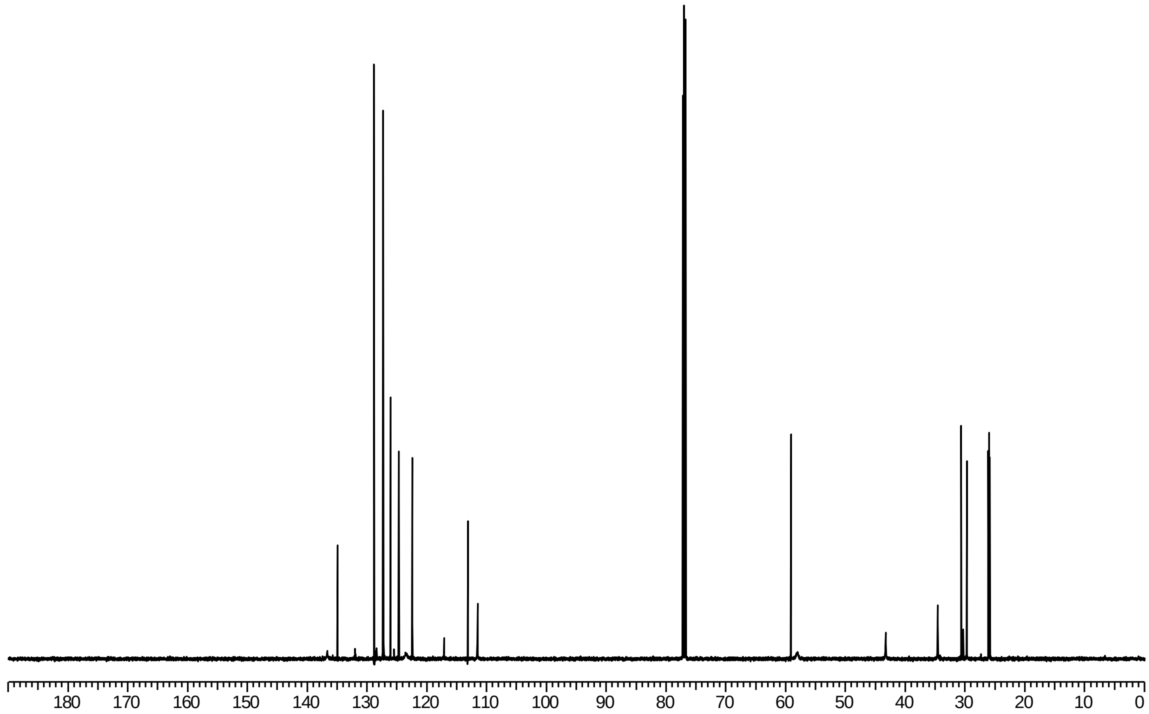

Supplementary figure 22. ^{1}H and ^{13}C NMR spectra for product **2gee**

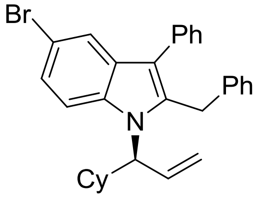

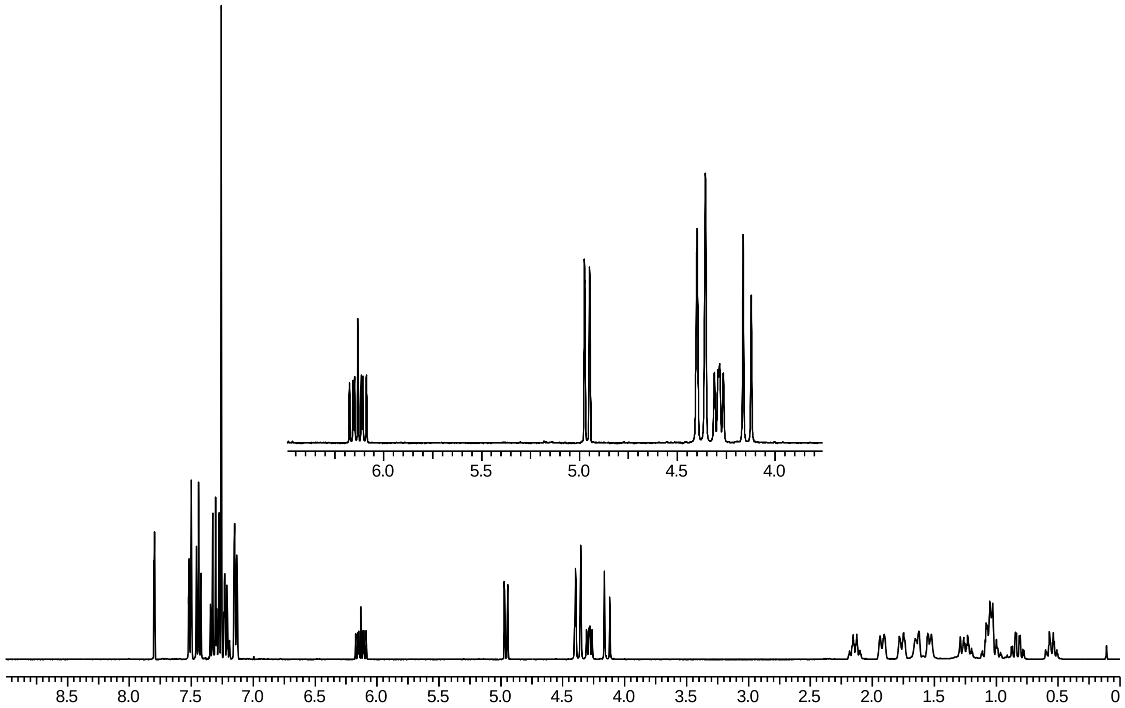

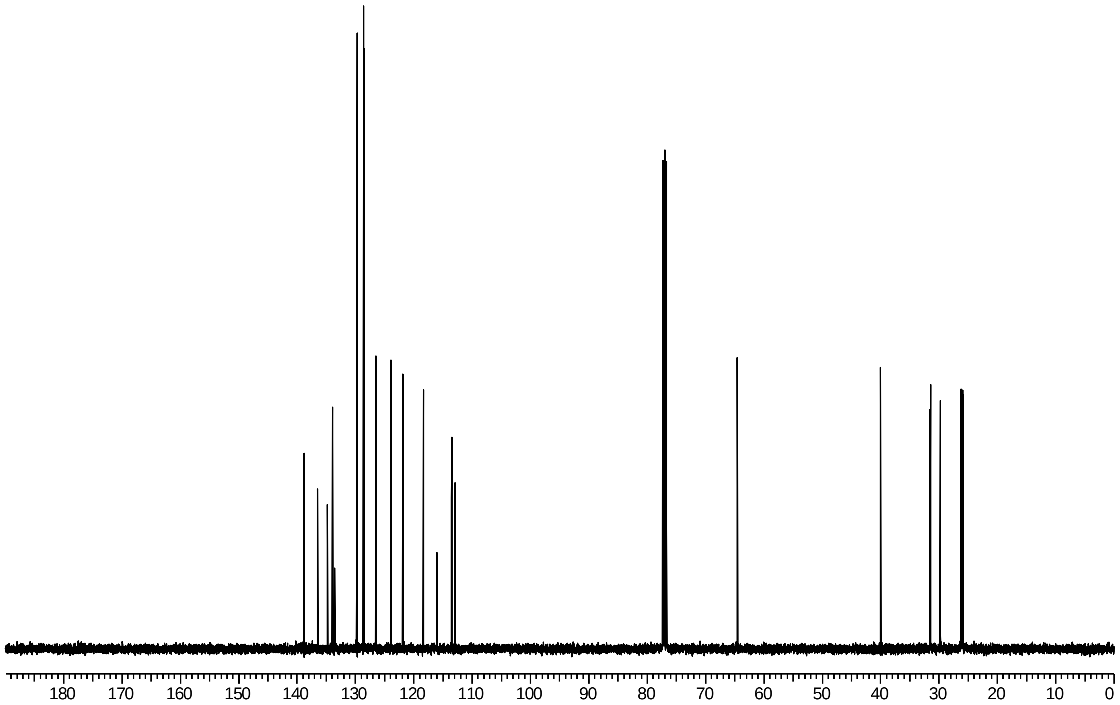

Supplementary figure 23. ^{1}H and ^{13}C NMR spectra for product **2h**

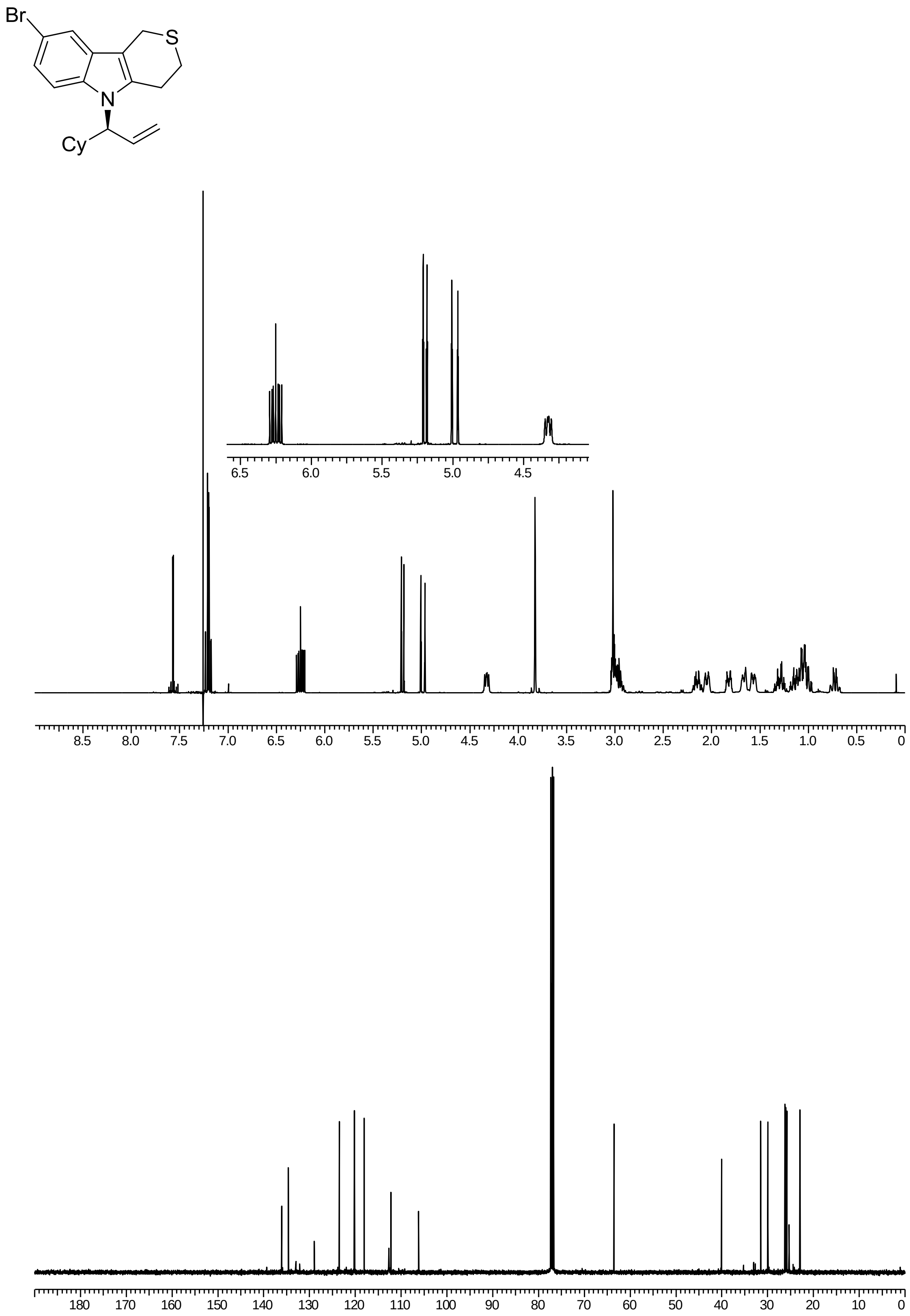

Supplementary figure 24. ^{1}H and ^{13}C NMR spectra for product **2i**

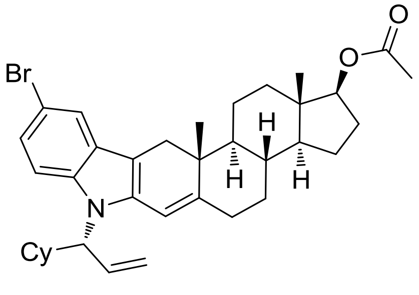

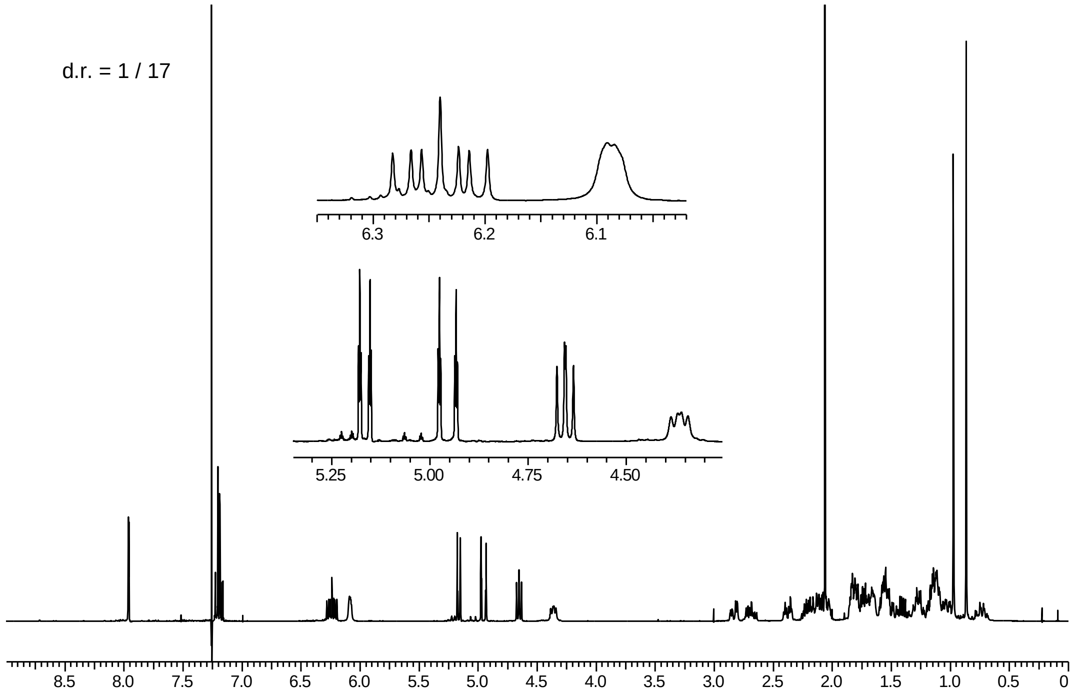

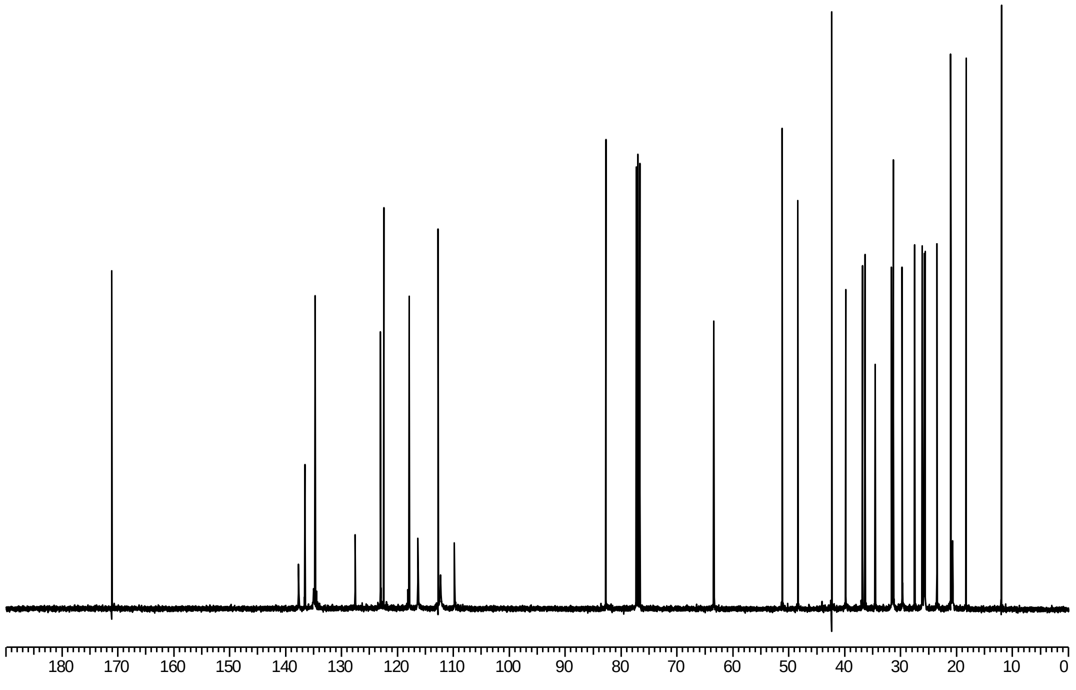

Supplementary figure 25. ^{1}H and ^{13}C NMR spectra for product **3**

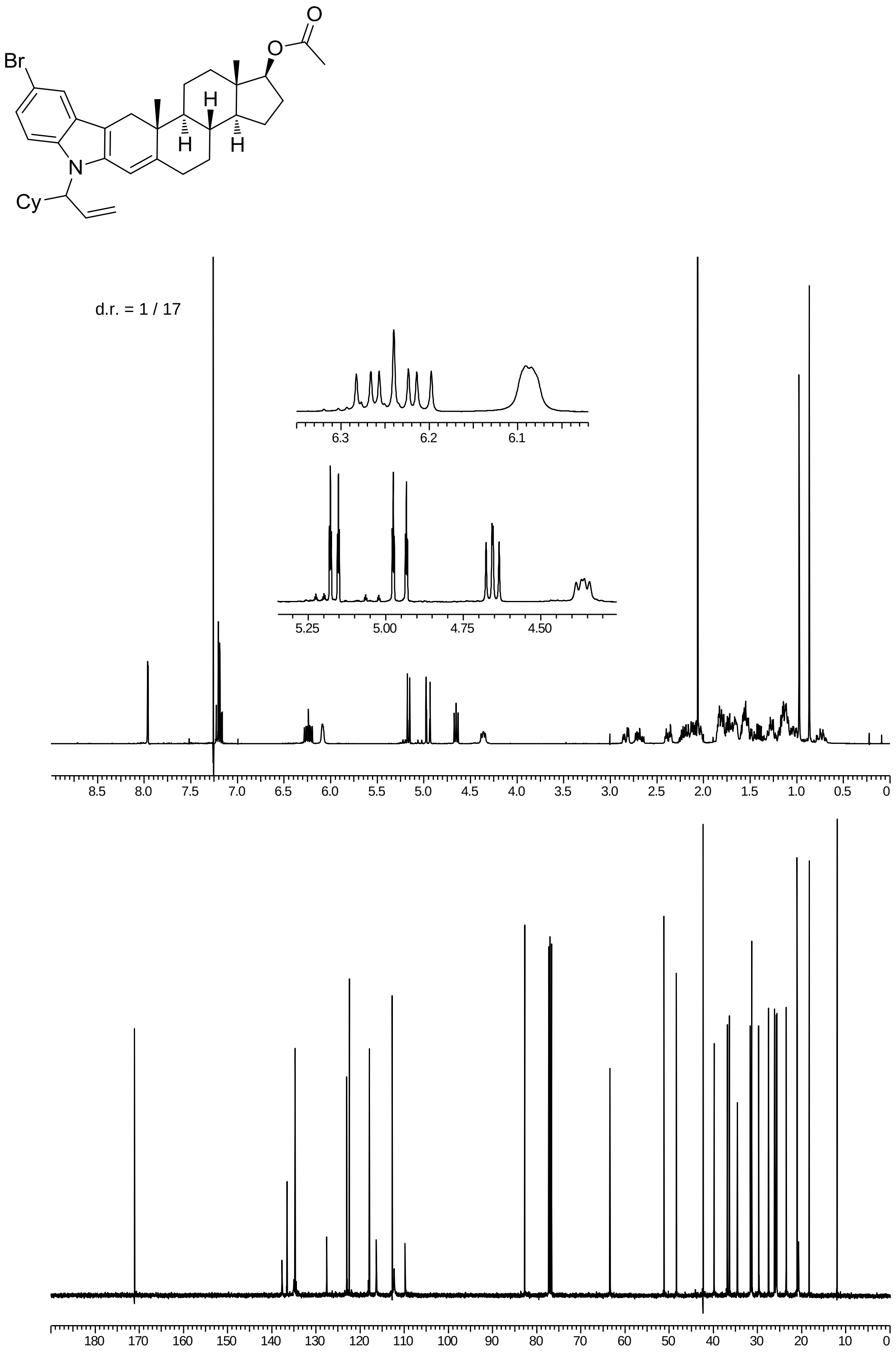

Supplementary figure 26. ^{1}H and ^{13}C NMR spectra for racemic product of **3**

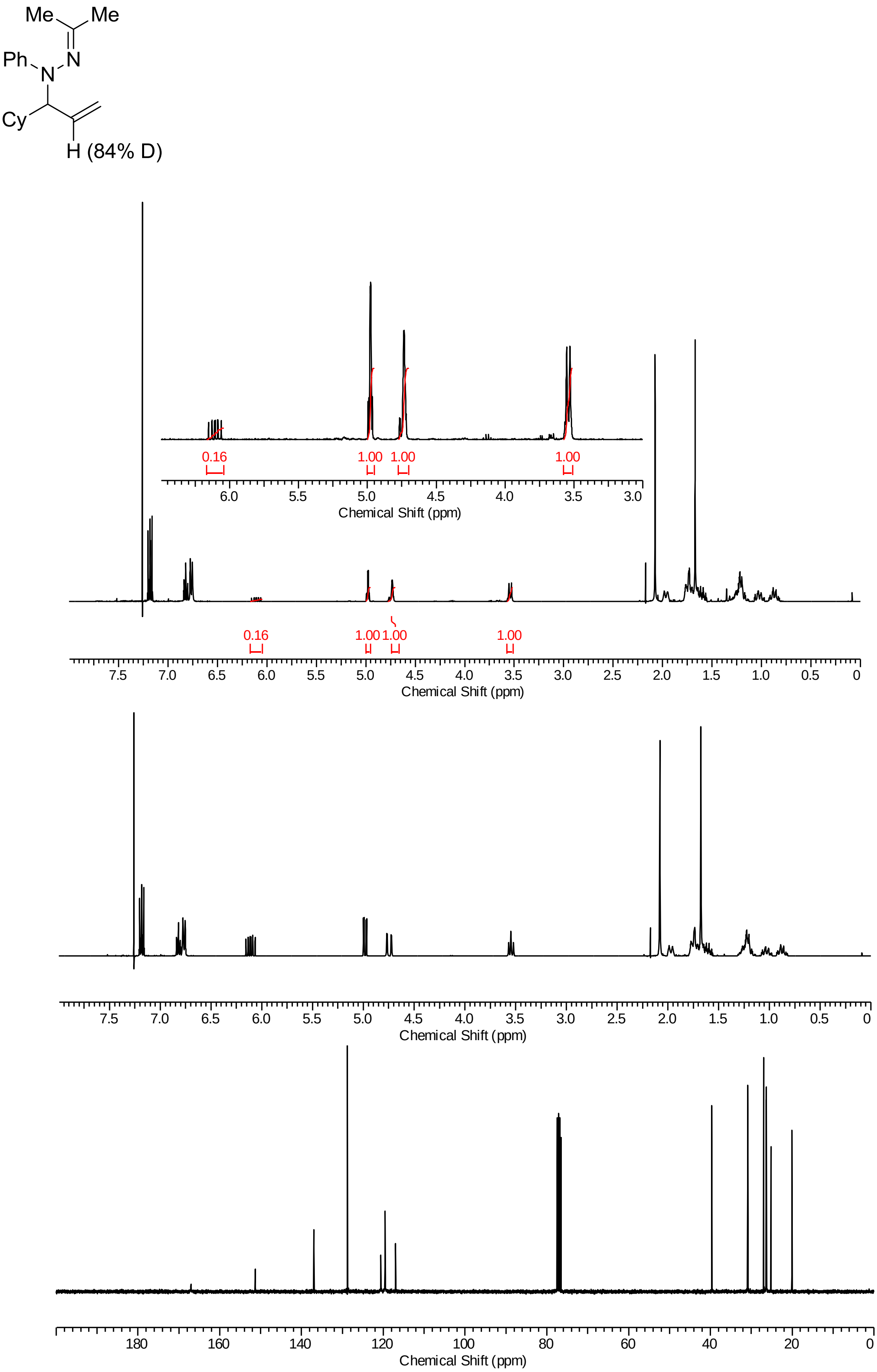

Supplementary figure 27. ^{1}H NMR of **5**, ^{1}H NMR and ^{13}C NMR of non-deuterated sample of **5**

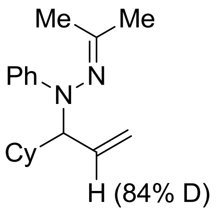

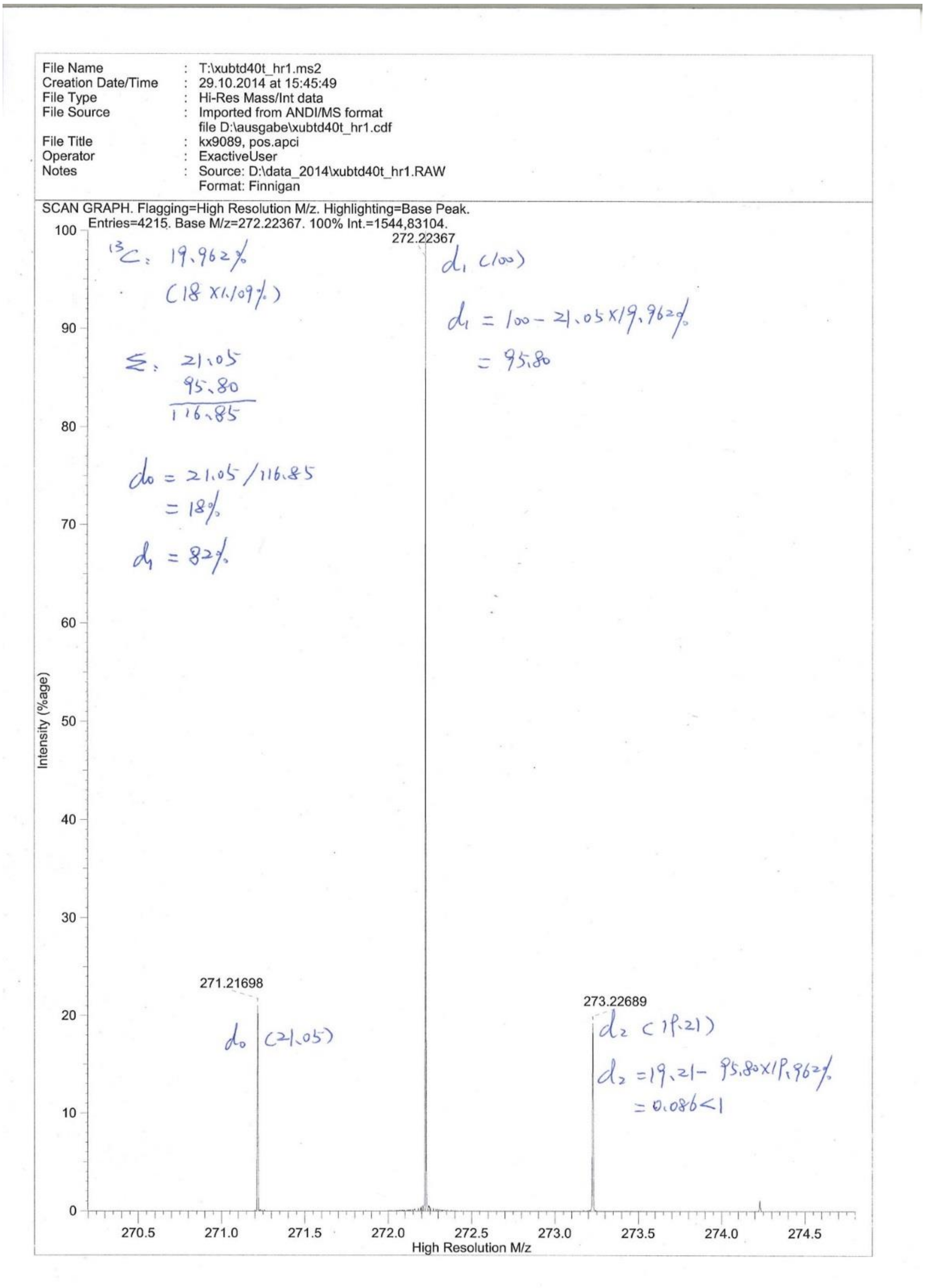

Supplementary figure 28. HRMS spectrum of **5**

^{1}H NMR (500 MHz, $CDCl_3$) at 263 K (Oxidative Addition: Rh-H formation)

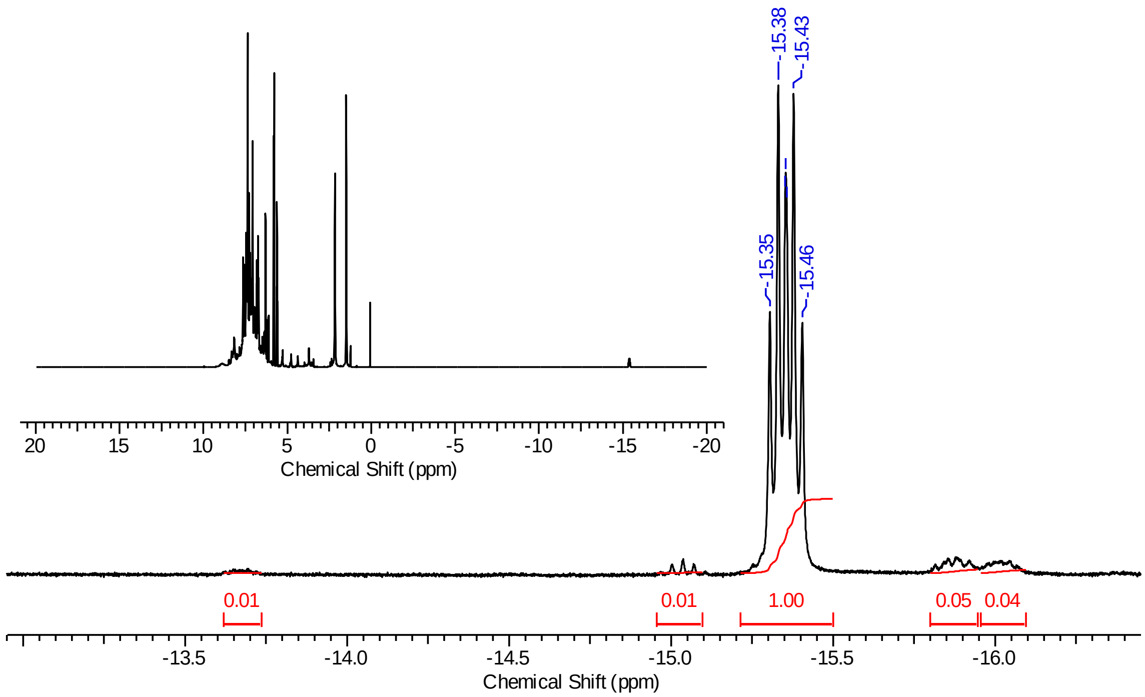

^{1}H NMR (500 MHz, $CDCl_3$) δ = -15.40 (d, J = 14.0 Hz, 1 H), 263 K, ^{31}P decoupled

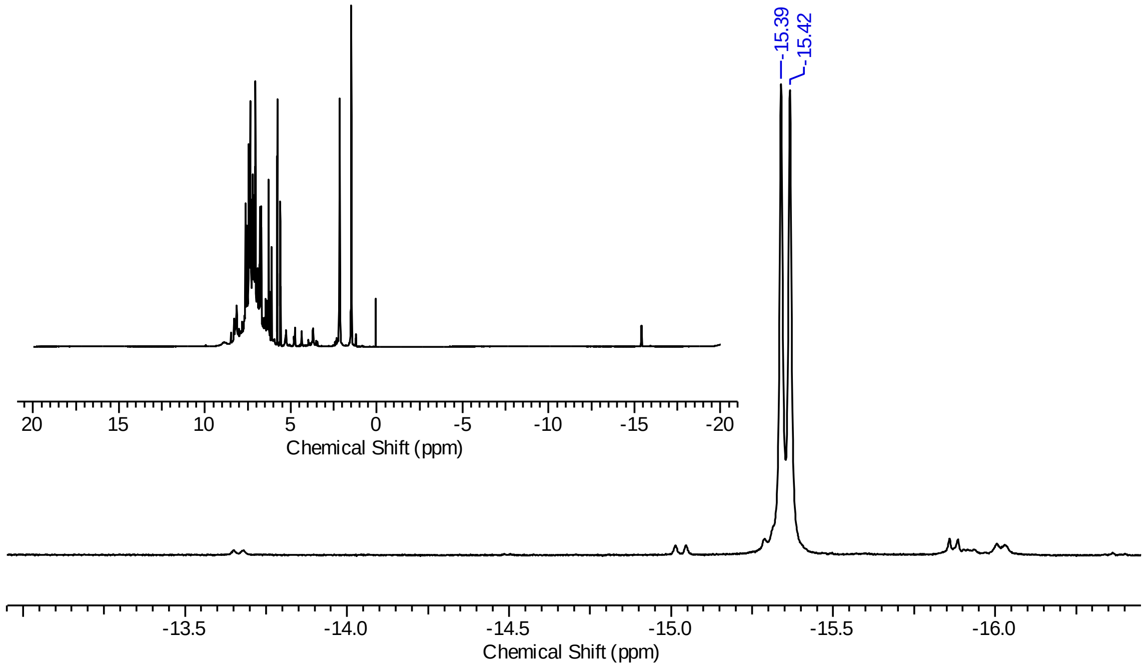

Supplementary figure 29. ^{1}H NMR for Stoichiometric Reaction of phenylhydrazine with catalysts

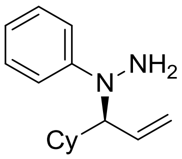

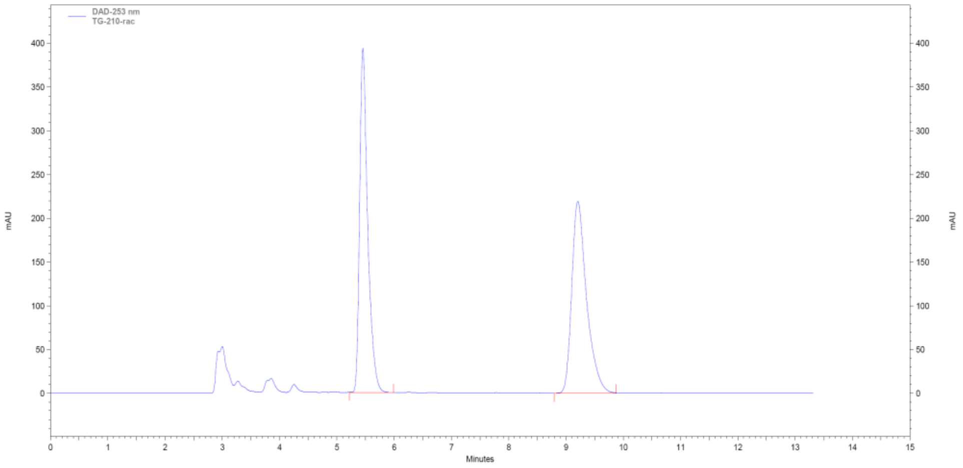

DAD-253 nm Results Pk #	Retention Time	Area Percent	Lambda Max
1	5,453	49,417	207
2	9,207	50,583	206

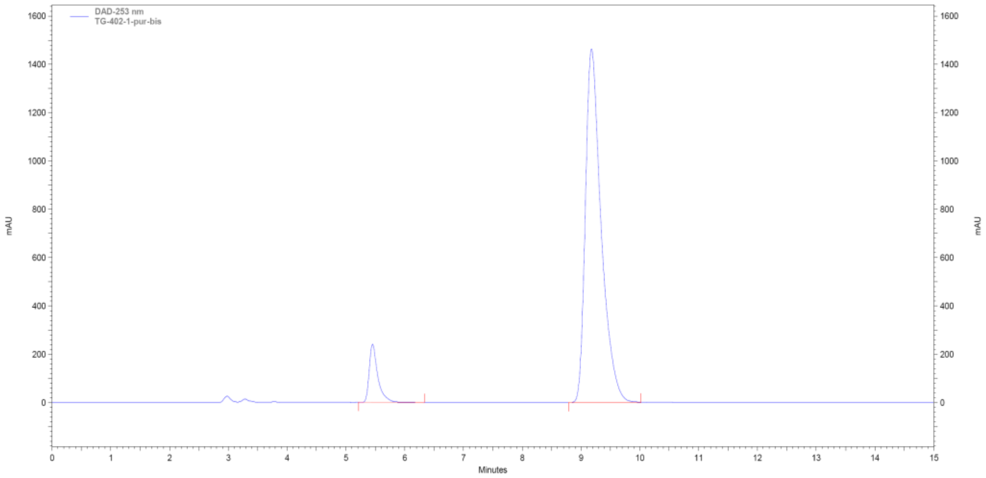

DAD-253 nm Results Pk #	Retention Time	Area Percent	Lambda Max
1	5,453	8,251	206
2	9,180	91,749	253

Supplementary figure 30. HPLC spectra for product **1a**

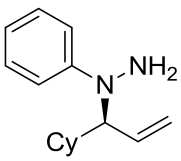

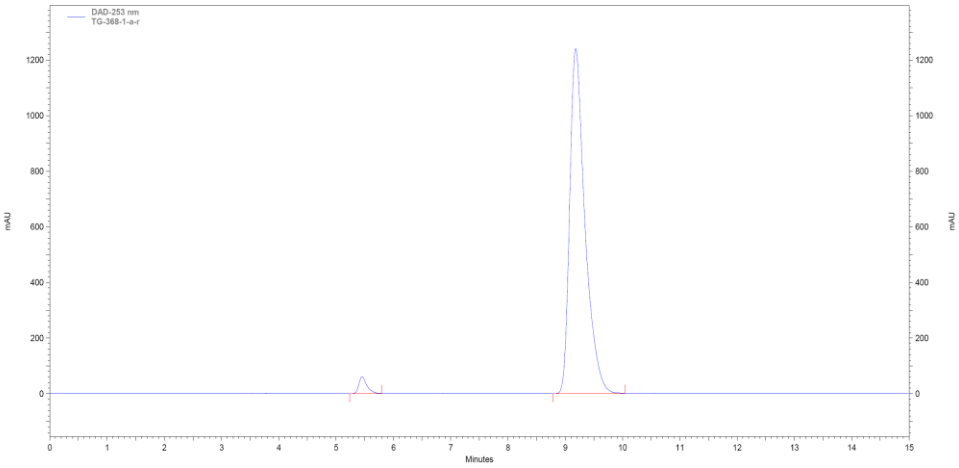

DAD-252 nm Results Pk #	Retention Time	Area Percent	Lambda Max
1	5,453	2,515	206
2	9,180	97,485	208

Supplementary figure 31. HPLC spectra for product **1a** (after recrystallization)

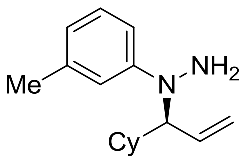

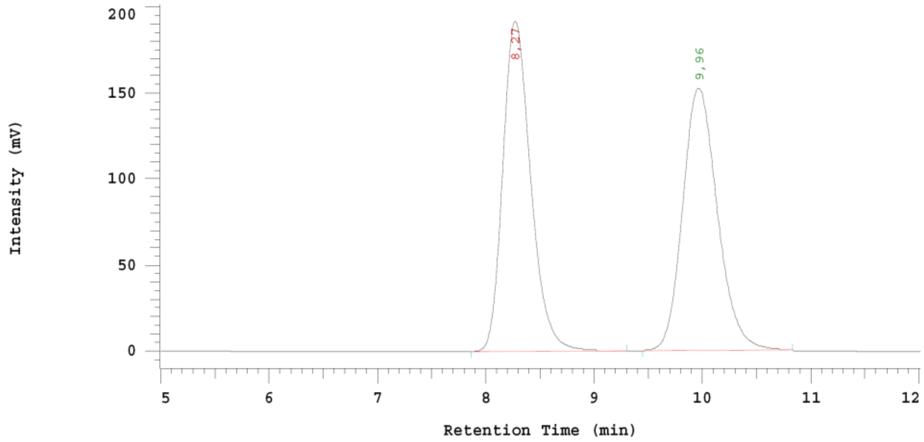

No.	RT	Area	Area %	BC
1	8,27	3376073	49,977	MC
2	9,96	3379226	50,023	BB
		6755299	100,000	

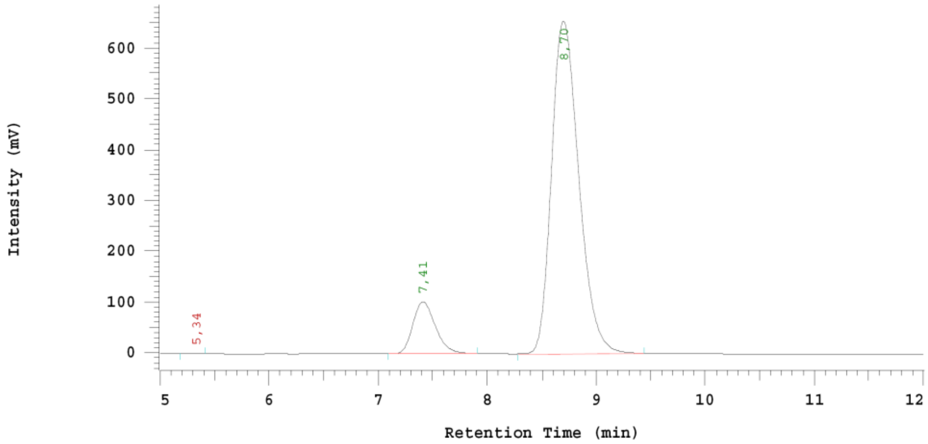

No.	RT	Area	Area %	BC
1	3,26	0	0,000	
2	3,63	0	0,000	
3	3,97	0	0,000	
4	5,34	0	0,000	
5	7,41	1439851	11,416	BB
6	8,70	11172604	88,584	BB
		12612455	100,000	

Supplementary figure 32. HPLC spectra for product **1b**

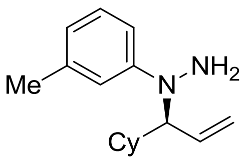

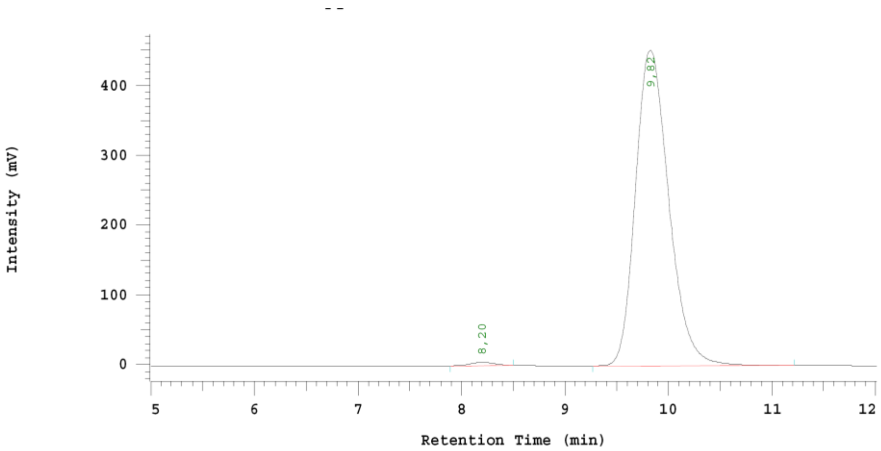

No.	RT	Area	Area %	BC
1	8,20	72110	0,716	BB
2	9,82	9998086	99,284	BB
3	14,60	0	0,000	
		10070196	100,000	

Supplementary figure 33. HPLC spectra for product **1b** (after recrystallization)

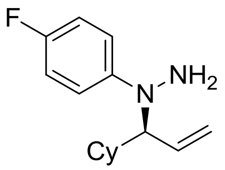

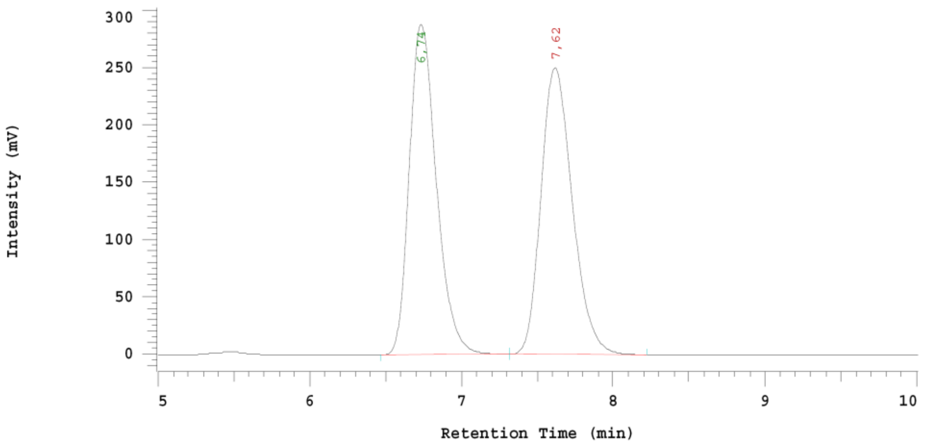

No.	RT	Area	Area %	BC
1	6,74	3517516	49,968	BB
2	7,62	3522080	50,032	MC
3	14,31	0	0,000	
		7039596	100,000	

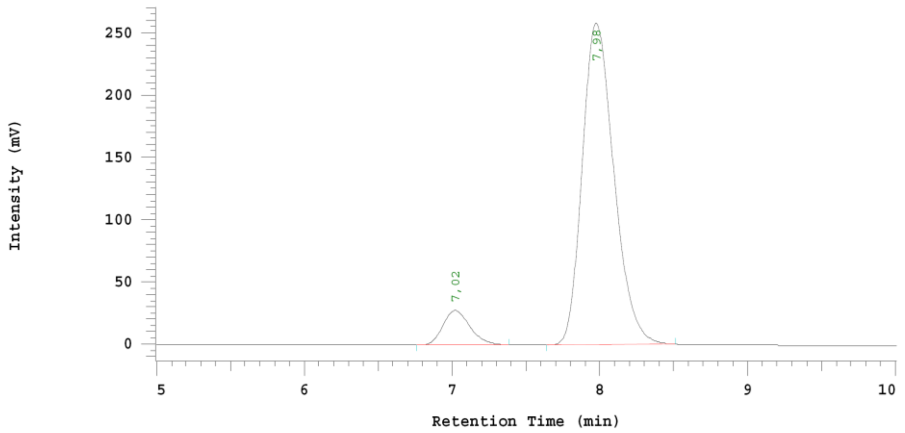

No.	RT	Area	Area %	BC
1	7,02	346681	8,343	BB
2	7,98	3808726	91,657	BB
		4155407	100,000	

Supplementary figure 34. HPLC spectra for product **1c**

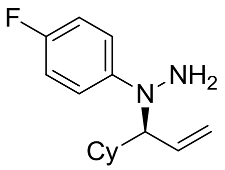

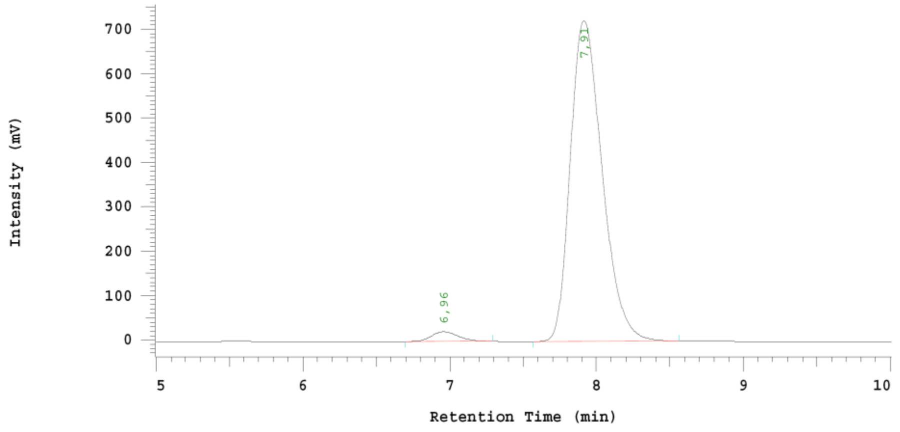

No.	RT	Area	Area %	BC
1	6,96	267823	2,435	BB
2	7,91	10729172	97,565	BB
		10996995	100,000	

Supplementary figure 35. HPLC spectra for product **1c** (after recrystallization)

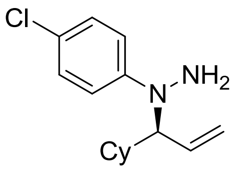

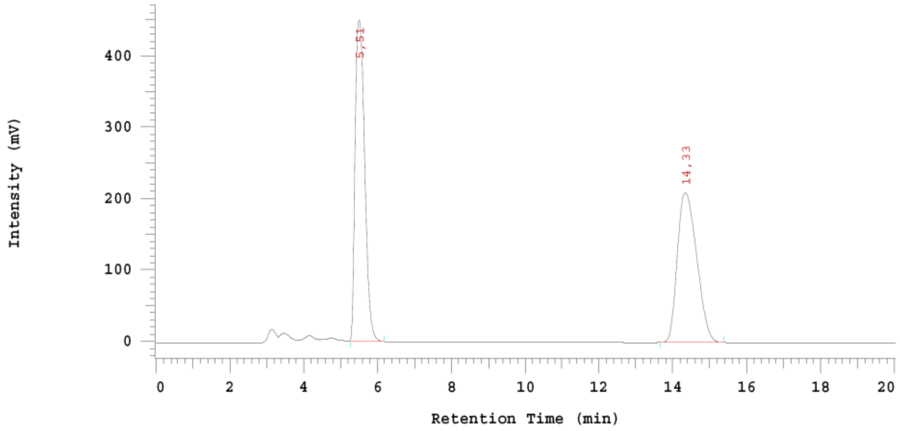

No.	RT	Area	Area %	BC
1	5,51	7409244	50,037	MC
2	14,33	7398314	49,963	MC
		14807558	100,000	

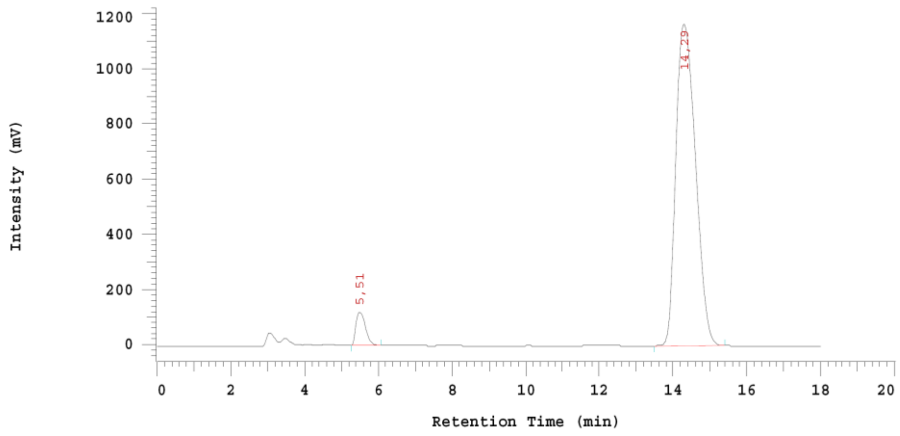

No.	RT	Area	Area %	BC
1	5,51	2013450	4,602	MC
2	14,29	41735130	95,398	MC
		43748580	100,000	

Supplementary figure 36. HPLC spectra for product **1d**

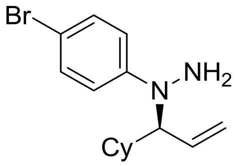

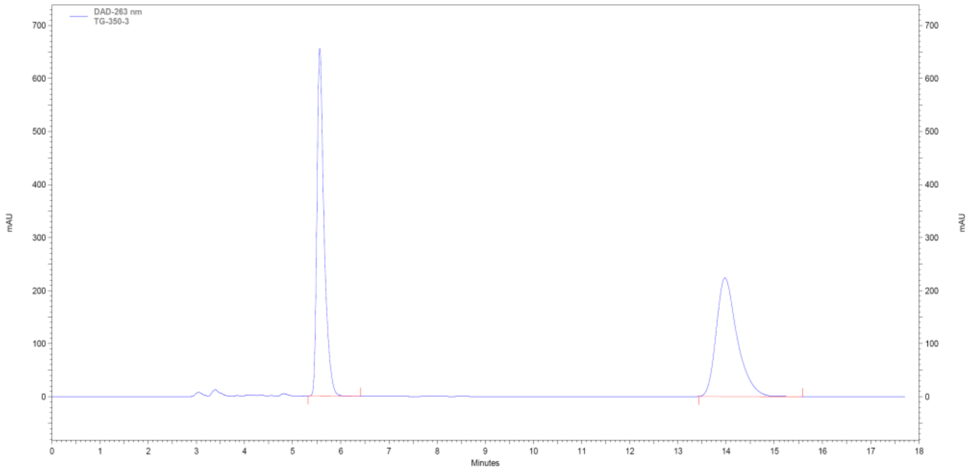

DAD-263 nm Results Pk #	Retention Time	Area Percent	Lambda Max
1	5,560	50,025	262
2	13,973	49,975	262

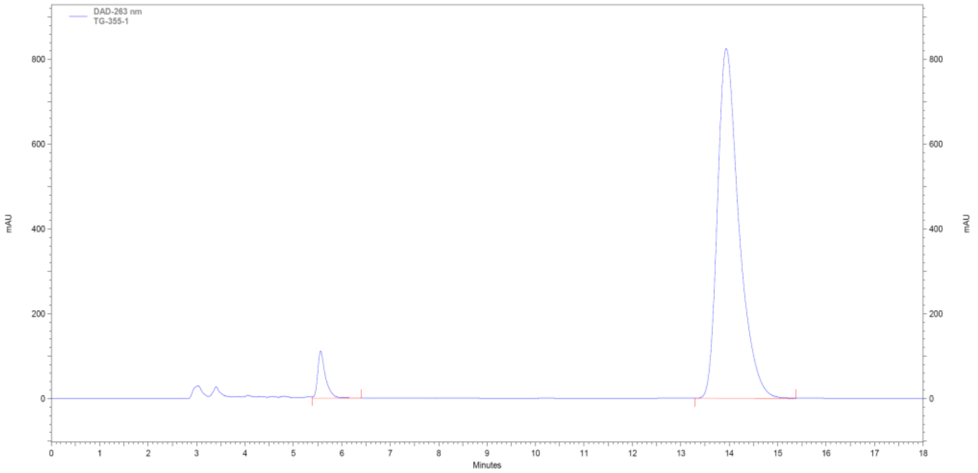

DAD-263 nm Results Pk #	Retention Time	Area Percent	Lambda Max
1	5,560	4,667	262
2	13,940	95,333	262

Supplementary figure 37. HPLC spectra for product **1e**

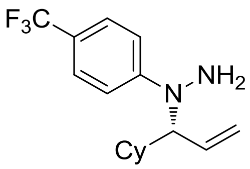

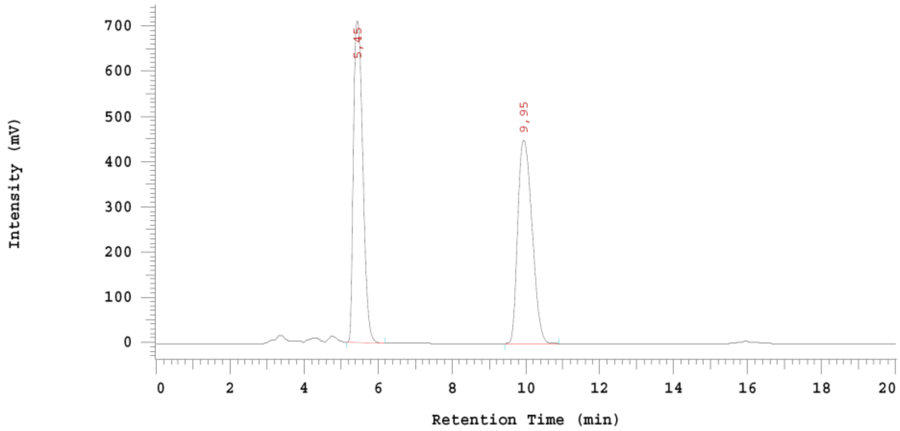

No.	RT	Area	Area %	BC
1	5,45	11459357	49,823	MC
2	9,95	11540588	50,177	MC
		22999945	100,000	

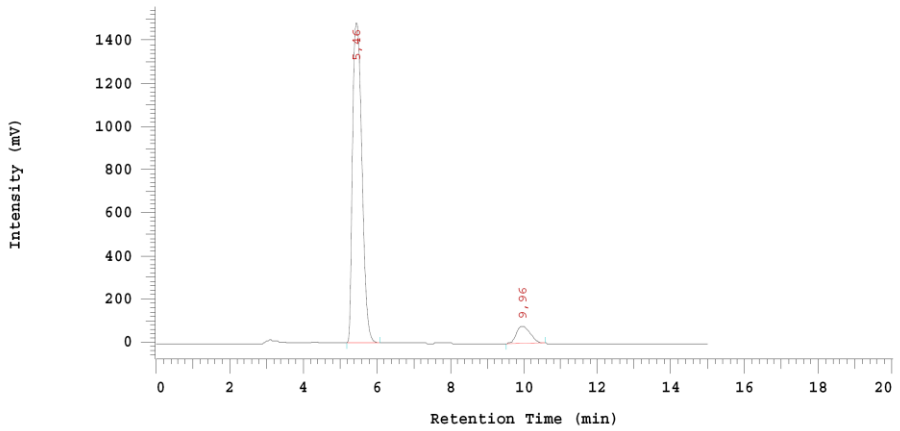

No.	RT	Area	Area %	BC
1	5,46	24229995	92,409	MC
2	9,96	1990272	7,591	MC
		26220267	100,000	

Supplementary figure 38. HPLC spectra for product **1f**

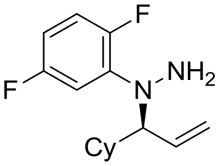

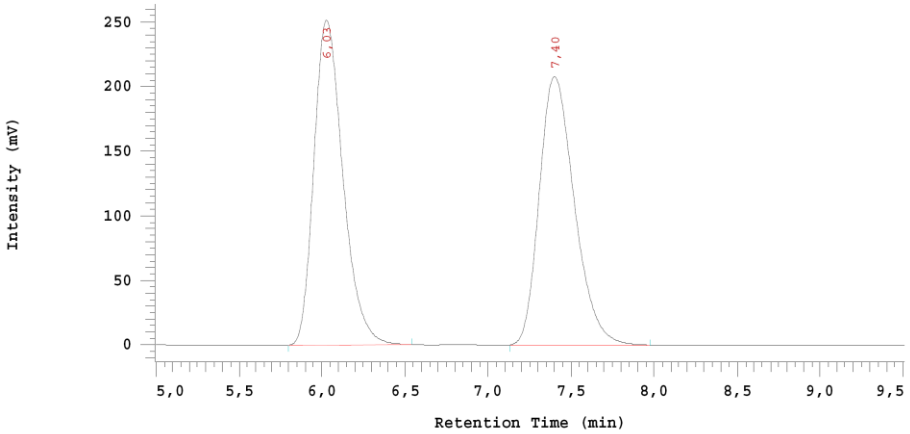

No.	RT	Area	Area %	BC
1	6,03	3026685	49,498	MC
2	7,40	3016955	49,339	MC
3	12,61	71120	1,163	BB
		6114760	100,000	

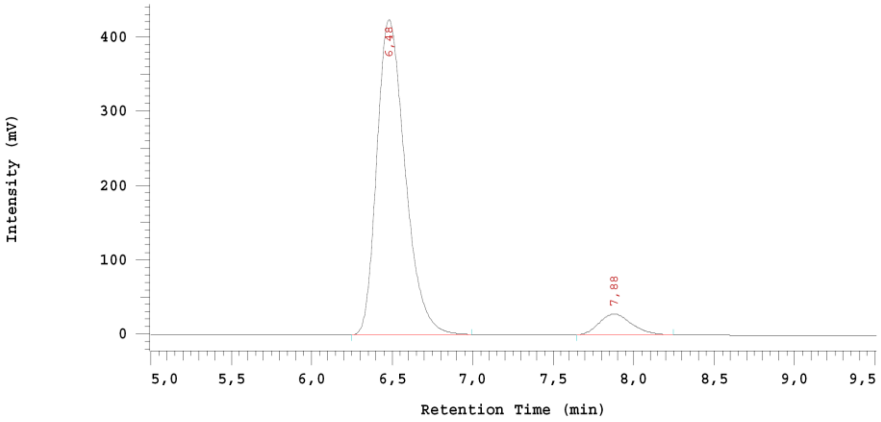

No.	RT	Area	Area %	BC
1	6,48	5099496	92,877	MC
2	7,88	391099	7,123	MC
		5490595	100,000	

Supplementary figure 39. HPLC spectra for product **1g**

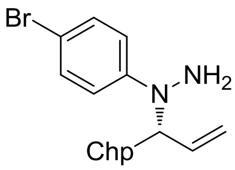

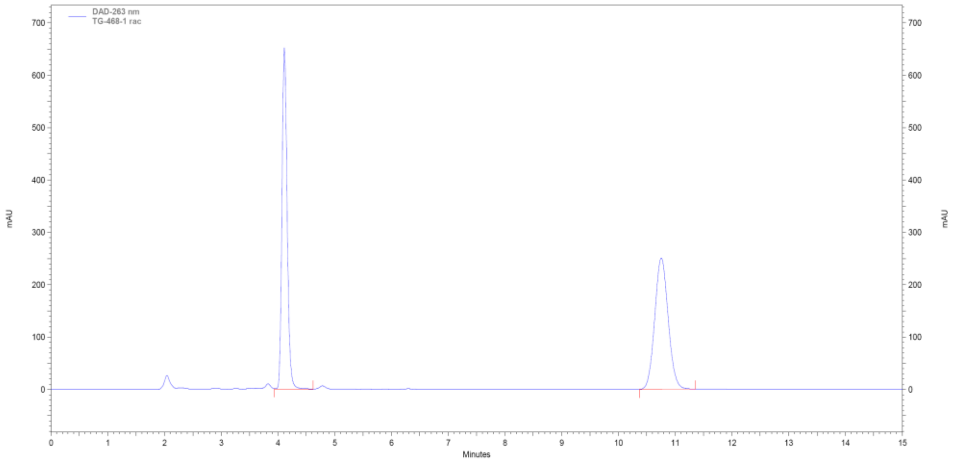

DAD-263 nm Results Pk #	Retention Time	Area Percent	Lambda Max
1	4,113	50,020	262
2	10,753	49,980	262

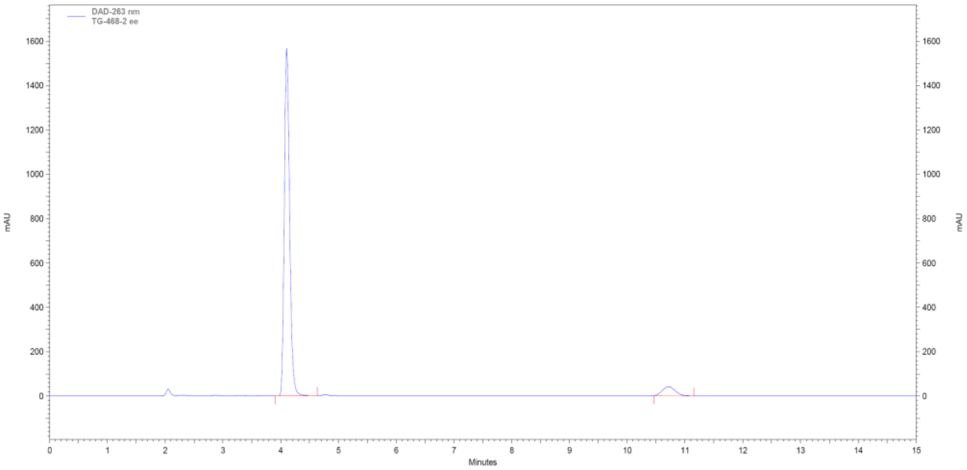

DAD-263 nm Results Pk #	Retention Time	Area Percent	Lambda Max
1	4,107	94,022	262
2	10,713	5,978	206

Supplementary figure 40. HPLC spectra for product **1h**

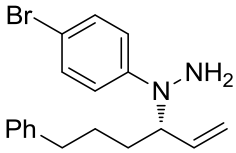

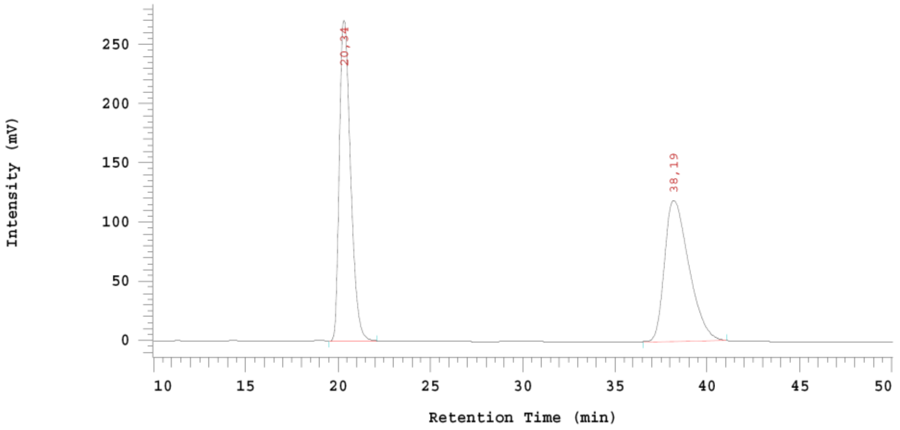

No.	RT	Area	Area %	BC
1	20,34	10696940	50,135	MC
2	38,19	10639236	49,865	MC
		21336176	100,000	

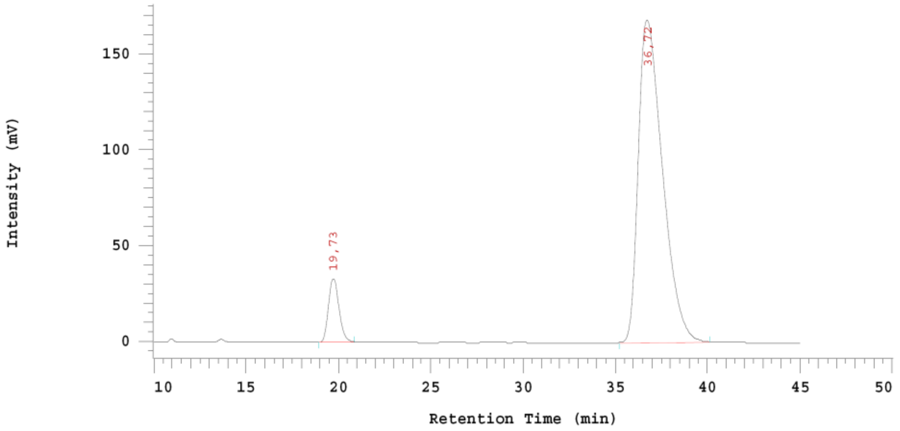

No.	RT	Area	Area %	BC
1	19,73	1256508	7,774	MC
2	36,72	14906753	92,226	MC
		16163261	100,000	

Supplementary figure 41. HPLC spectra for product **1i**

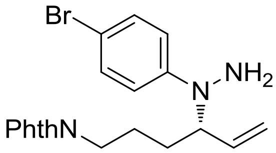

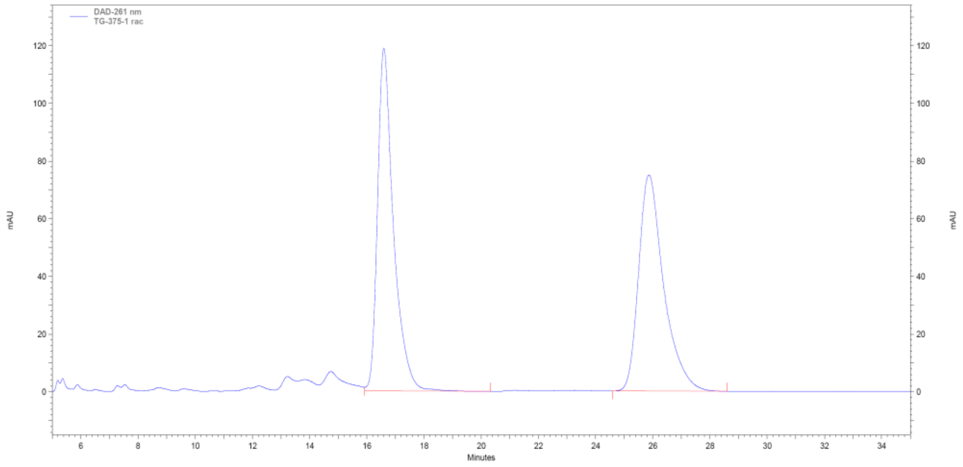

DAD-261 nm
Results

Pk #	Retention Time	Area Percent	Lambda Max
1	16,593	50,321	218
2	25,867	49,679	218

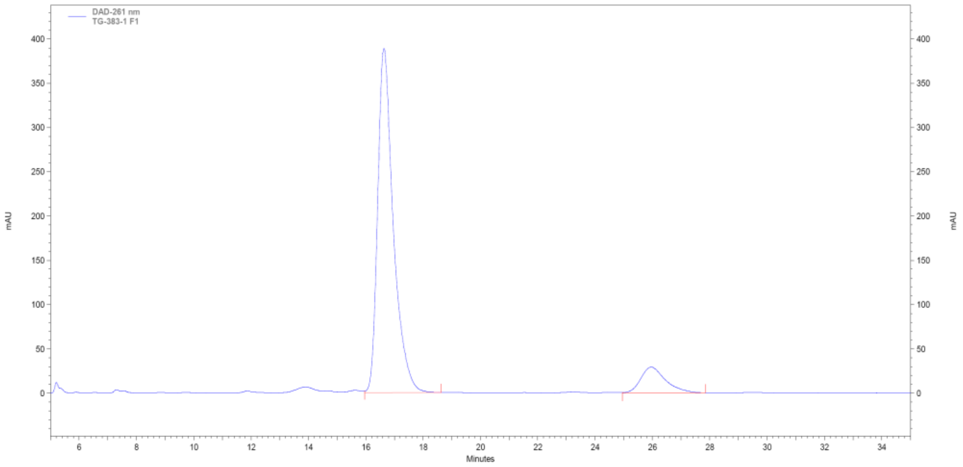

DAD-261 nm
Results

Pk #	Retention Time	Area Percent	Lambda Max
1	16,633	89,226	218
2	25,953	10,774	218

Supplementary figure 42. HPLC spectra for product **1j**

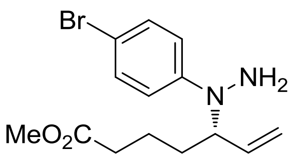

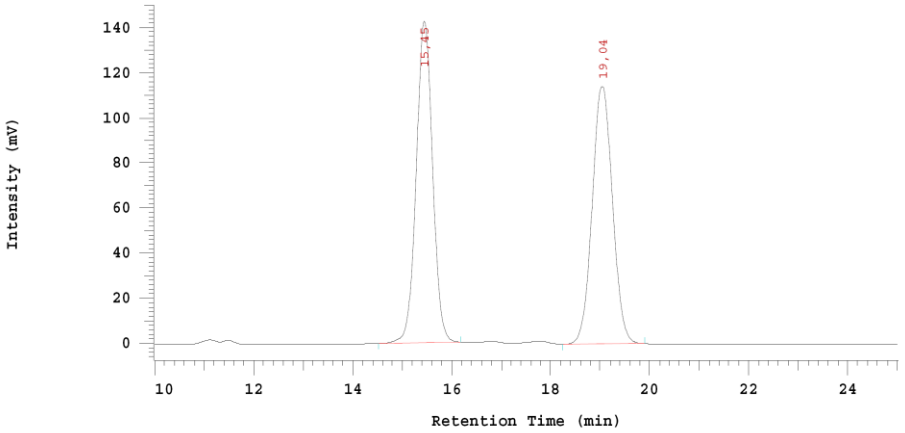

No.	RT	Area	Area %	BC
1	15,45	3193747	50,278	MC
2	19,04	3158431	49,722	MC
		6352178	100,000	

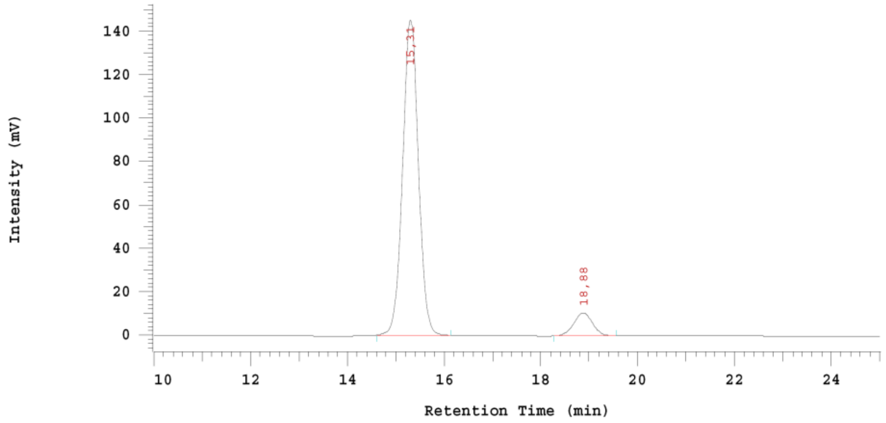

No.	RT	Area	Area %	BC
1	15,31	3211045	91,928	MC
2	18,88	281966	8,072	MC
		3493011	100,000	

Supplementary figure 43. HPLC spectra for product **1k**

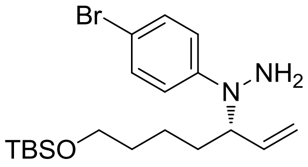

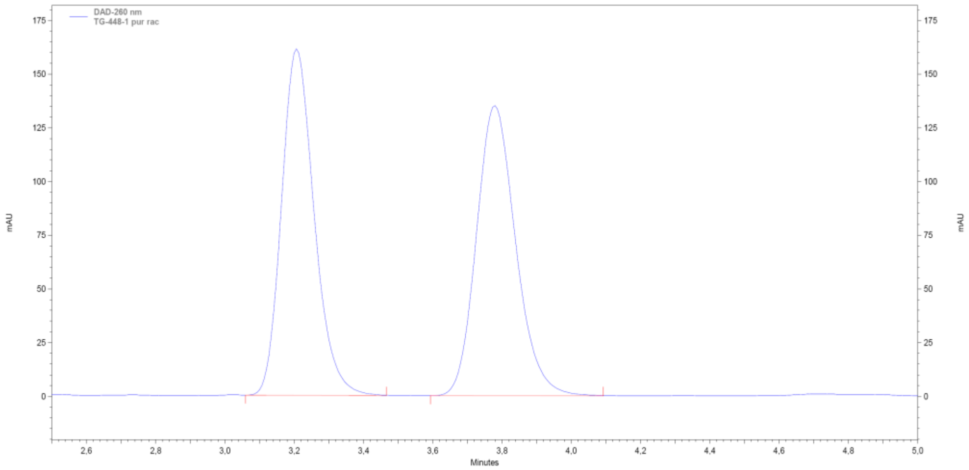

DAD-260 nm
Results

Pk #	Retention Time	Area Percent	Lambda Max
1	3,207	49,819	206
2	3,780	50,181	206

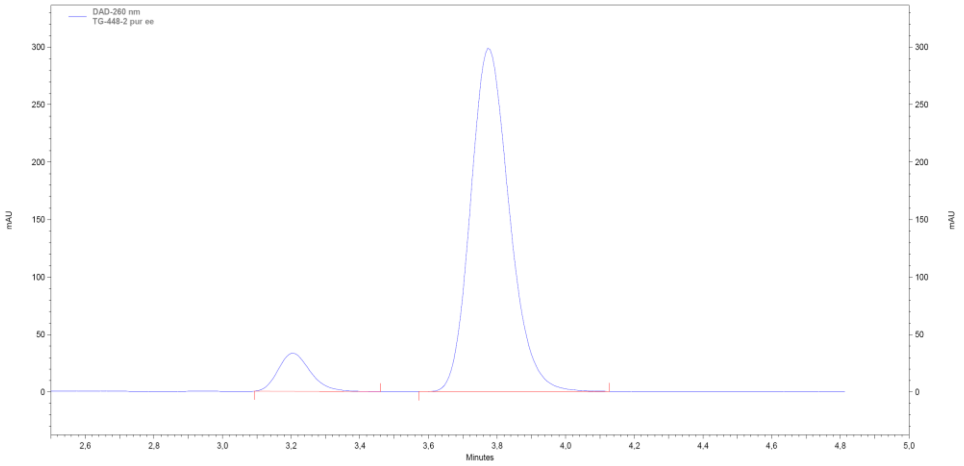

DAD-260 nm
Results

Pk #	Retention Time	Area Percent	Lambda Max
1	3,207	8,419	206
2	3,773	91,581	206

Supplementary figure 44. HPLC spectra for product **1l**

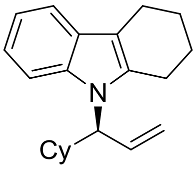

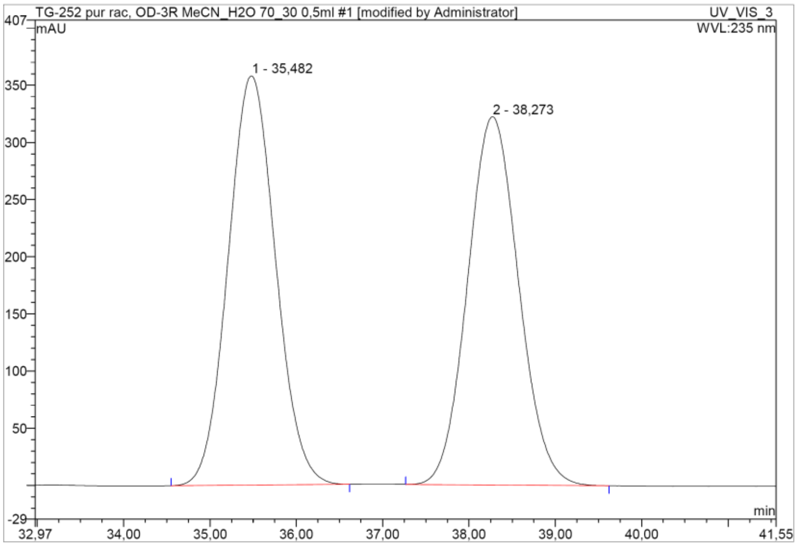

No.	Ret.Time min	Peak Name	Height mAU	Area mAU*min	Rel.Area %	Amount n.a.	Type
1	35,48	n.a.	358,066	228,086	50,81	n.a.	BMB*
2	38,27	n.a.	322,130	220,789	49,19	n.a.	BMB*
Total:			680,195	448,874	100,00	0,000	

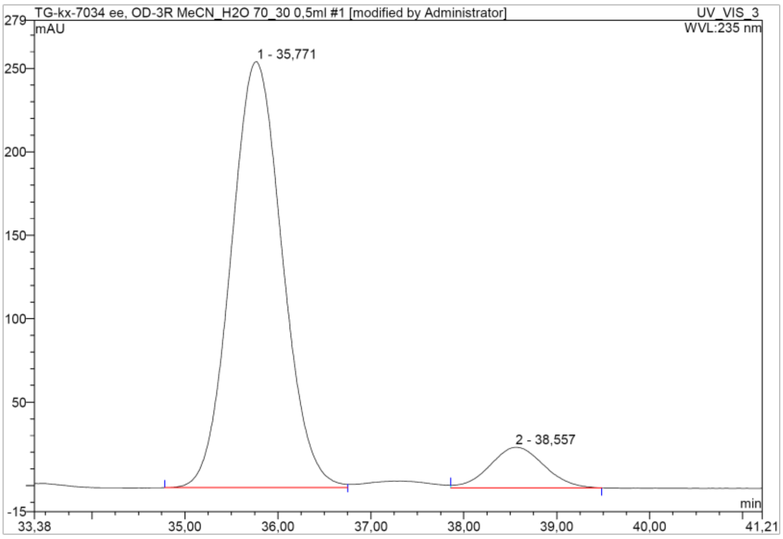

No.	Ret.Time min	Peak Name	Height mAU	Area mAU*min	Rel.Area %	Amount n.a.	Type
1	35,77	n.a.	255,377	163,246	90,80	n.a.	BM *
2	38,56	n.a.	24,444	16,550	9,20	n.a.	MB*
Total:			279,821	179,795	100,00	0,000	

Supplementary figure 45. HPLC spectra for product **2a**

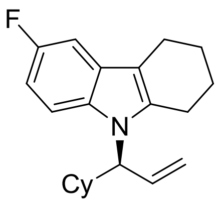

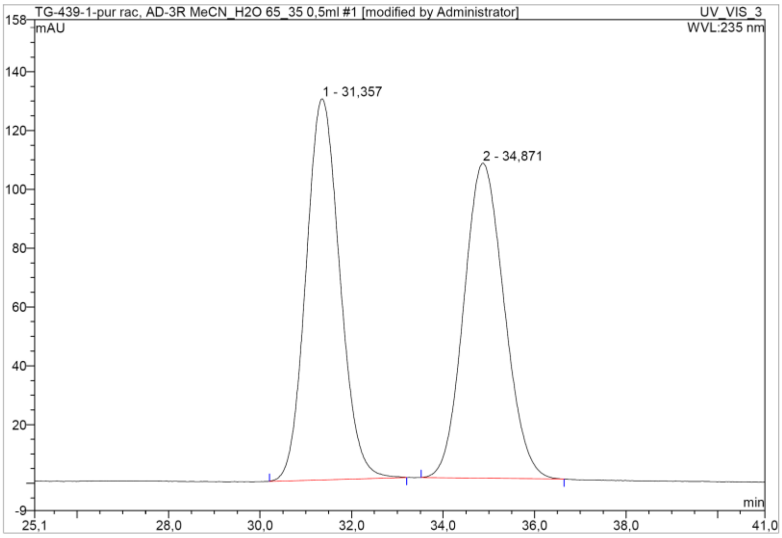

No.	Ret.Time min	Peak Name	Height mAU	Area mAU*min	Rel.Area %	Amount n.a.	Type
1	31,36	n.a.	129,617	111,023	50,32	n.a.	BMB*
2	34,87	n.a.	107,134	109,632	49,68	n.a.	BMB*
Total:			236,751	220,656	100,00	0,000	

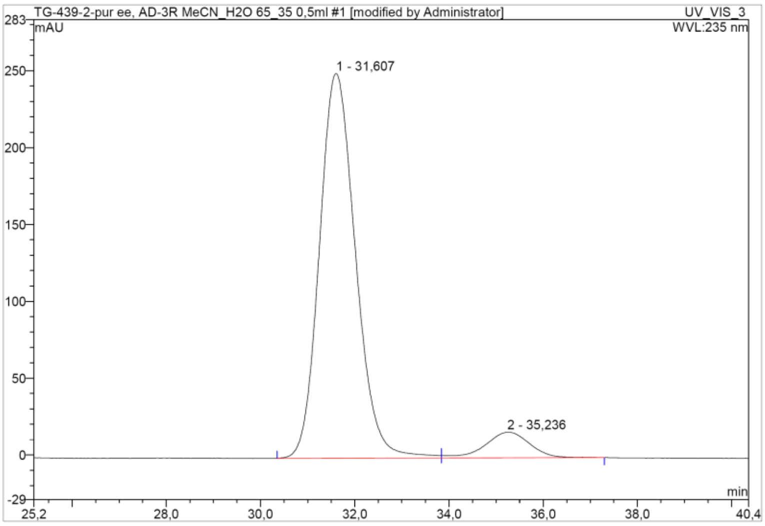

No.	Ret.Time min	Peak Name	Height mAU	Area mAU*min	Rel.Area %	Amount n.a.	Type
1	31,61	n.a.	250,382	222,091	92,33	n.a.	BM
2	35,24	n.a.	16,781	18,446	7,67	n.a.	MB
Total:			267,162	240,537	100,00	0,000	

Supplementary figure 46. HPLC spectra for product **2b**

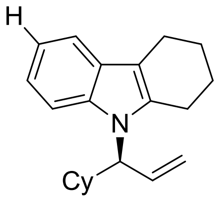

derivative of **2c**

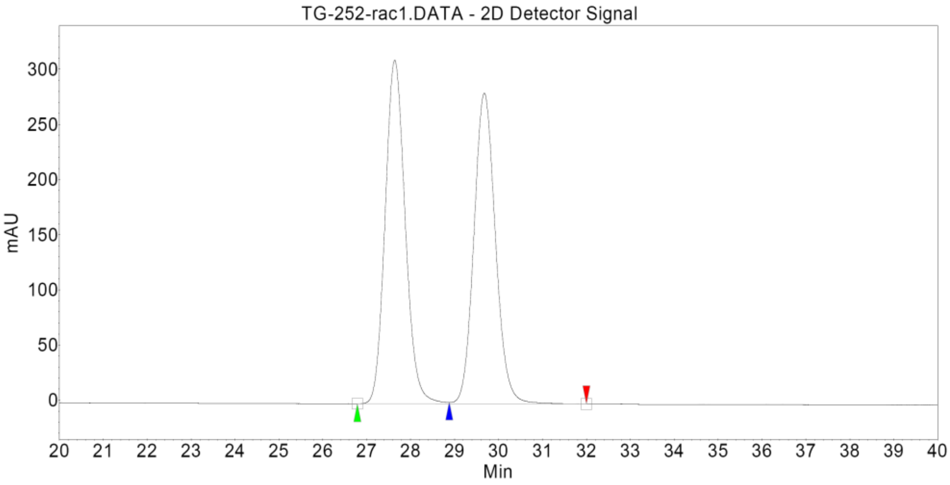

Index	Name	Time [Min]	Quantity [% Area]	Height [mAU]	Area [mAU.Min]	Area % [%]
1	UNKNOWN	27,64	49,62	311,7	165,4	49,622
2	UNKNOWN	29,68	48,03	281,8	160,1	48,032
3	UNKNOWN	54,86	2,35	7,5	7,8	2,346
Total			100,00	601,0	333,4	100,000

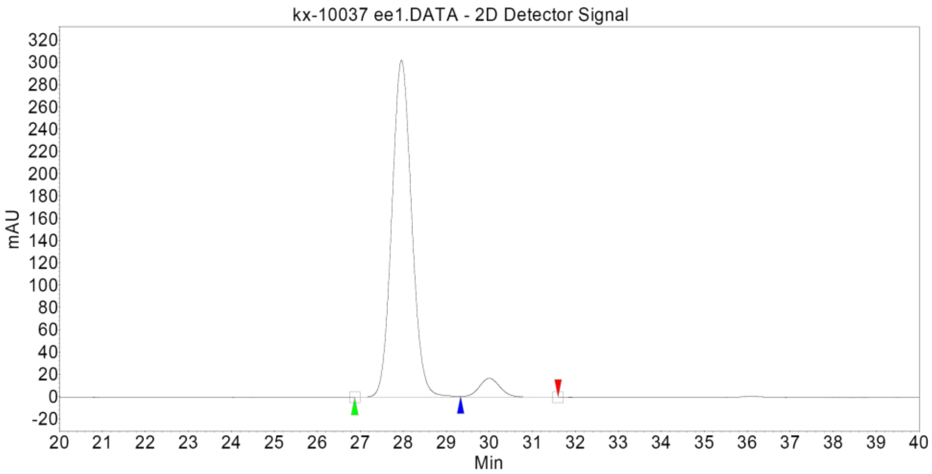

Index	Name	Time [Min]	Quantity [% Area]	Height [mAU]	Area [mAU.Min]	Area % [%]
1	UNKNOWN	27,96	94,32	302,4	161,9	94,320
2	UNKNOWN	30,00	5,68	17,0	9,7	5,680
Total			100,00	319,4	171,6	100,000

Supplementary figure 47. HPLC spectra for product **2cee**

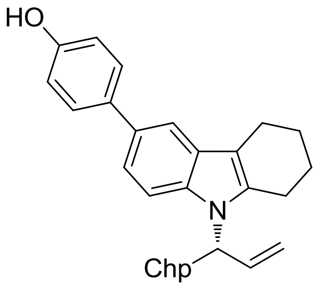

derivative of **2d**

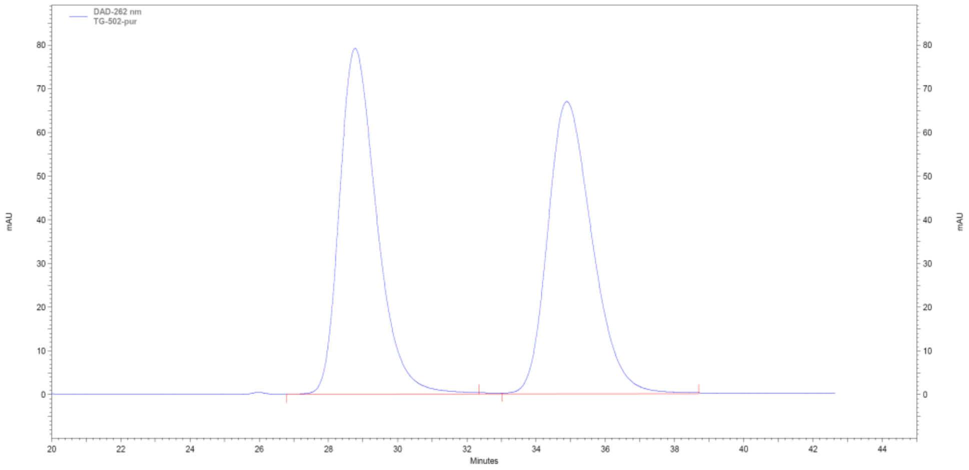

DAD-262 nm
Results

Pk #	Retention Time	Area Percent	Lambda Max
1	29,147	6,489	262
2	34,700	93,511	262

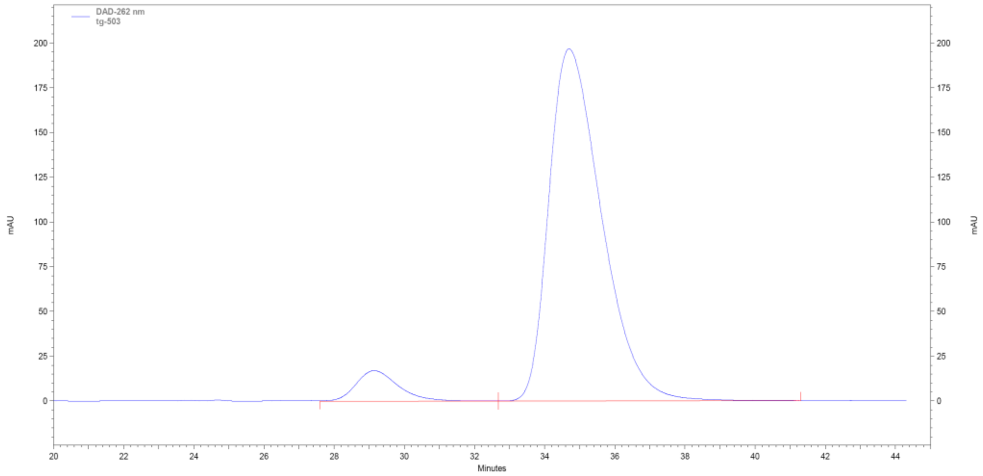

DAD-262 nm
Results

Pk #	Retention Time	Area Percent	Lambda Max
1	29,147	6,489	262
2	34,700	93,511	262

Supplementary figure 48. HPLC spectra for product **2dee**

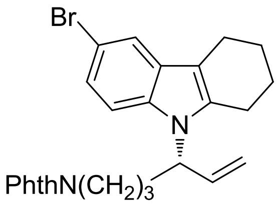

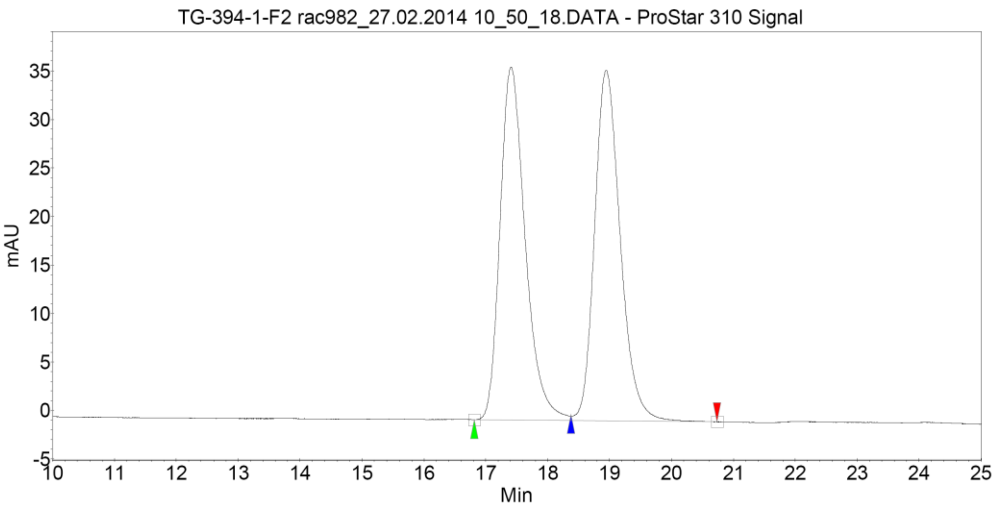

Index	Name	Time [Min]	Quantity [% Area]	Height [mAU]	Area [mAU.Min]	Area % [%]
1	UNKNOWN	17,41	49,67	36,4	17,1	49,674
2	UNKNOWN	18,94	50,33	36,1	17,4	50,326
Total			100,00	72,5	34,5	100,000

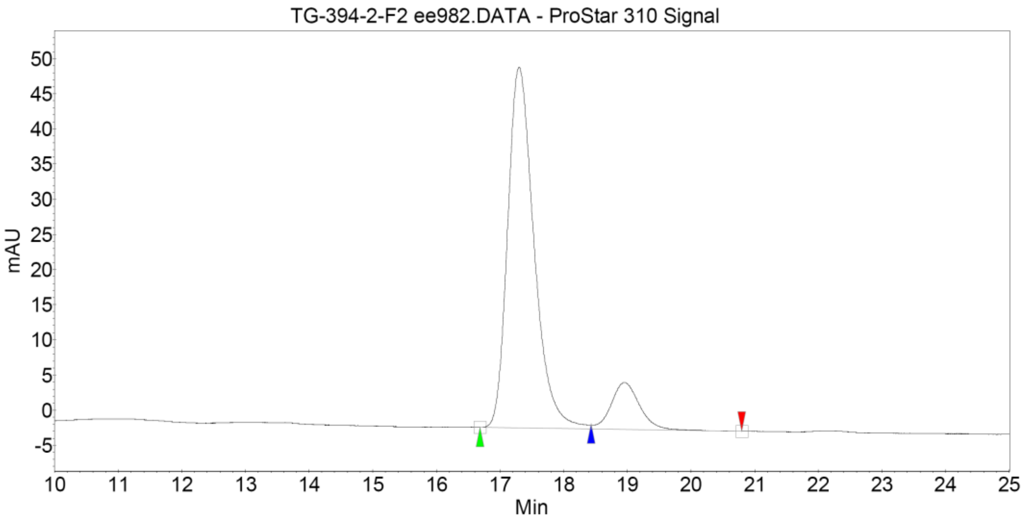

Index	Name	Time [Min]	Quantity [% Area]	Height [mAU]	Area [mAU.Min]	Area % [%]
1	UNKNOWN	17,30	87,66	51,3	24,6	87,655
2	UNKNOWN	18,95	12,34	6,7	3,5	12,345
Total			100,00	58,0	28,1	100,000

Supplementary figure 49. HPLC spectra for product **2e**

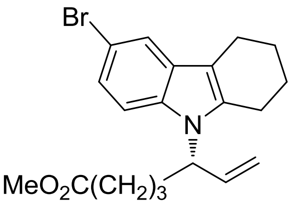

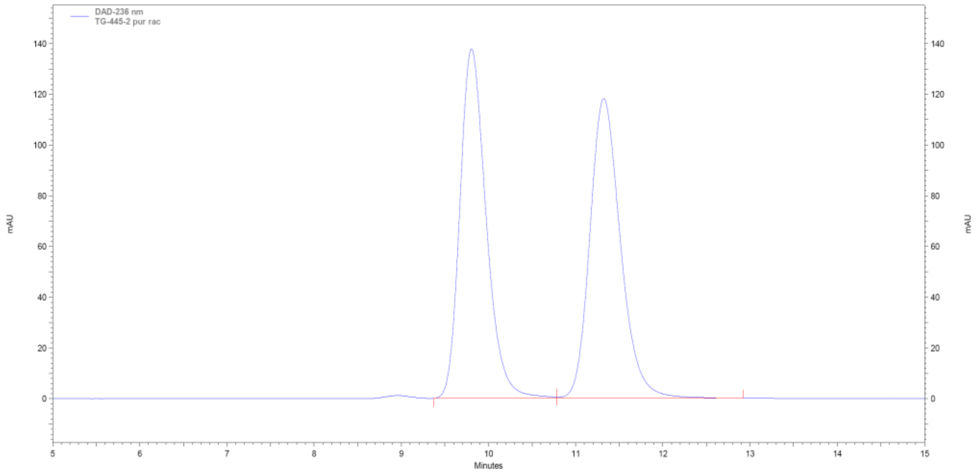

DAD-236 nm
Results

Pk #	Retention Time	Area Percent	Lambda Max
1	9,807	49,949	236
2	11,320	50,051	236

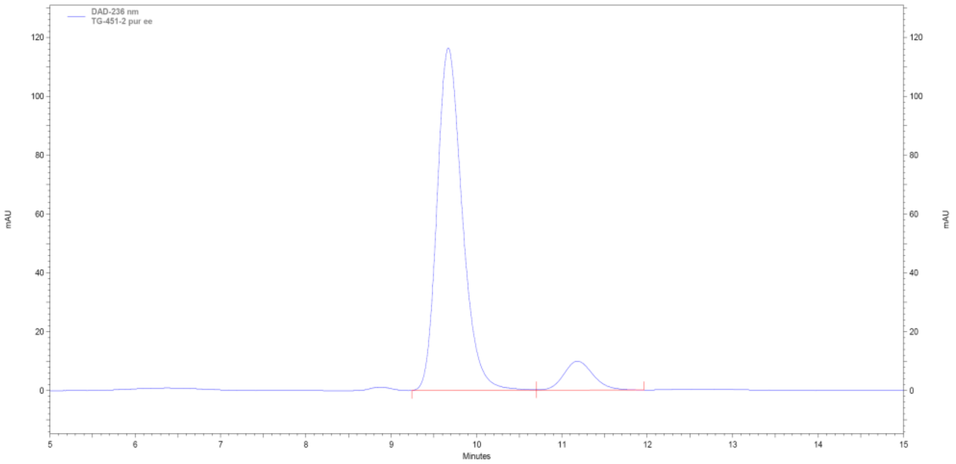

DAD-236 nm
Results

Pk #	Retention Time	Area Percent	Lambda Max
1	9,667	91,097	236
2	11,180	8,903	236

Supplementary figure 50. HPLC spectra for product **2f**

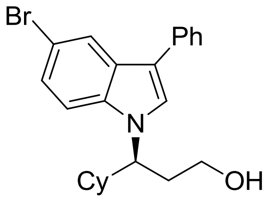

derivative of **2g**

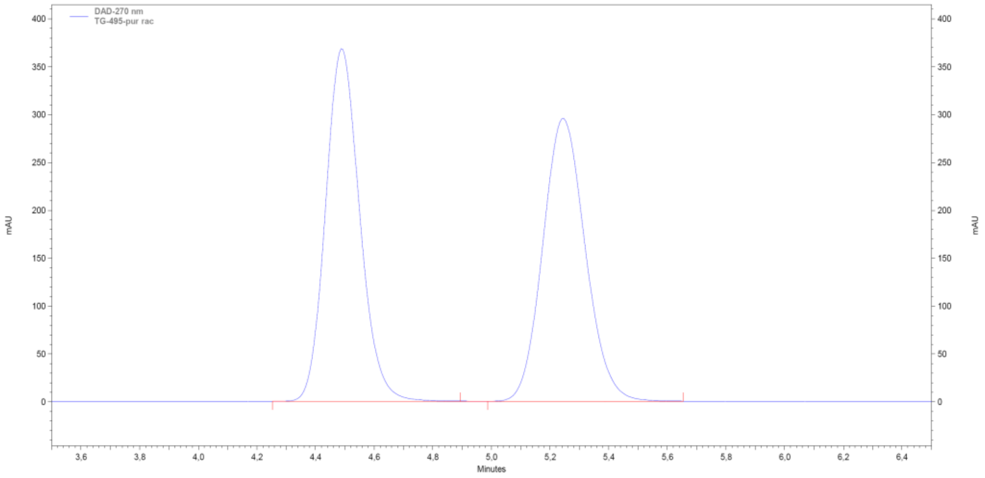

DAD-270 nm
Results

Pk #	Retention Time	Area Percent	Lambda Max
1	4,487	49,997	229
2	5,247	50,003	229

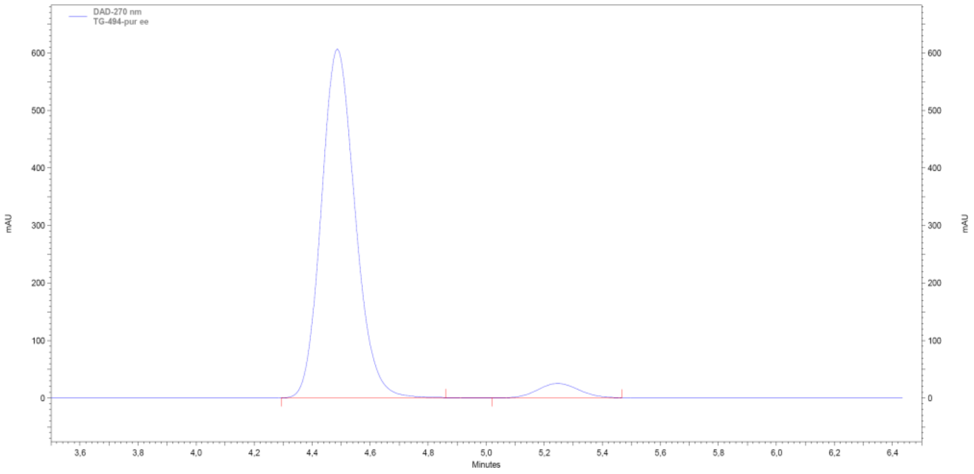

DAD-270 nm
Results

Pk #	Retention Time	Area Percent	Lambda Max
1	4,487	95,028	229
2	5,247	4,972	229

Supplementary figure 51. HPLC spectra for product **2gee**

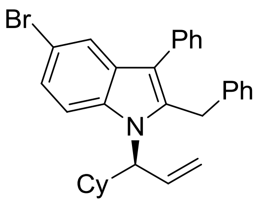

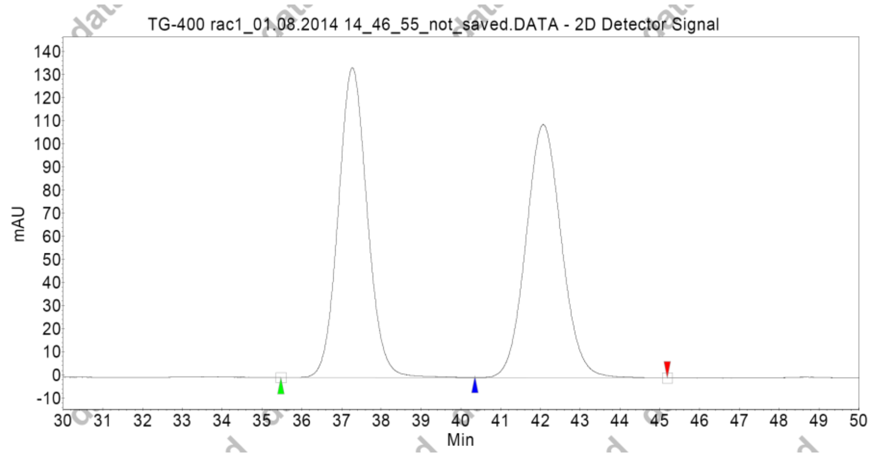

Index	Name	Time [Min]	Quantity [% Area]	Height [mAU]	Area [mAU.Min]	Area % [%]
1	UNKNOWN	37,27	49,96	134,1	113,5	49,957
2	UNKNOWN	42,07	50,04	109,7	113,7	50,043
Total			100,00	243,8	227,1	100,000

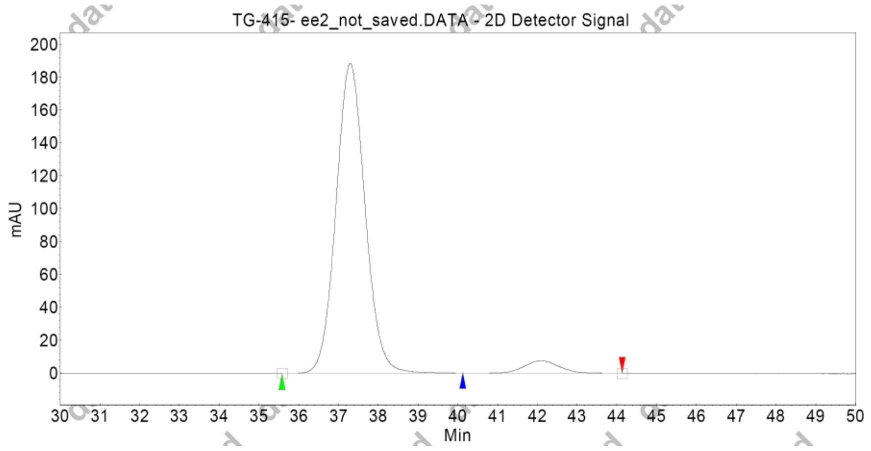

Index	Name	Time [Min]	Quantity [% Area]	Height [mAU]	Area [mAU.Min]	Area % [%]
1	UNKNOWN	37,29	95,26	188,6	160,1	95,262
2	UNKNOWN	42,09	4,74	7,7	8,0	4,738
Total			100,00	196,3	168,0	100,000

Supplementary figure 52. HPLC spectra for product **2h**

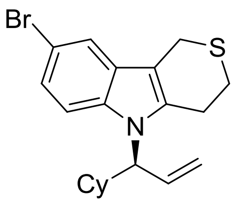

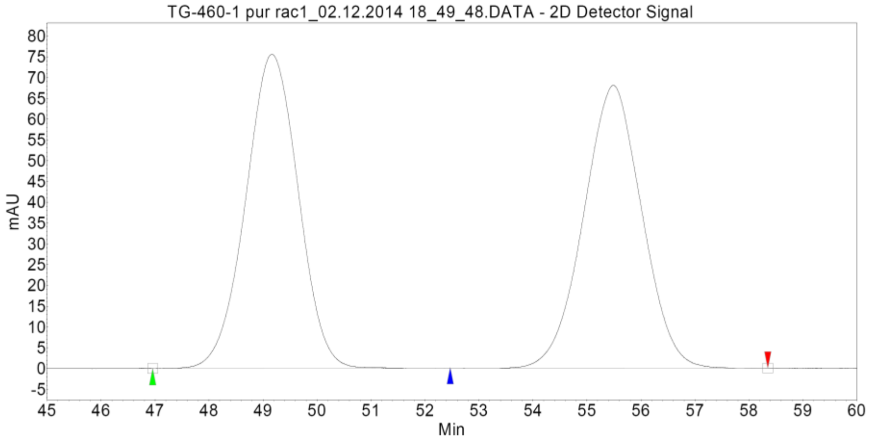

Index	Name	Time [Min]	Quantity [% Area]	Height [mAU]	Area [mAU.Min]	Area % [%]
1	UNKNOWN	49,16	50,05	75,5	85,5	50,052
2	UNKNOWN	55,49	49,95	68,1	85,3	49,948
Total			100,00	143,6	170,8	100,000

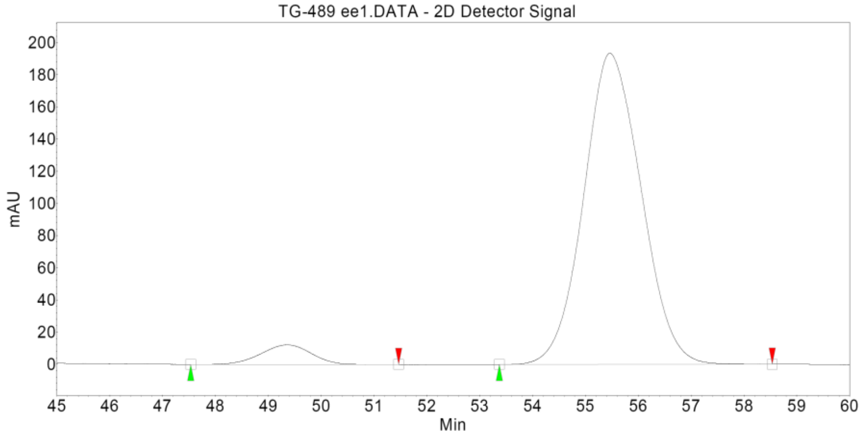

Index	Name	Time [Min]	Quantity [% Area]	Height [mAU]	Area [mAU.Min]	Area % [%]
1	UNKNOWN	49,36	5,21	12,2	13,4	5,214
2	UNKNOWN	55,45	94,79	193,4	243,9	94,786
Total			100,00	205,6	257,4	100,000

Supplementary figure 53. HPLC spectra for product **2i**

Supplementary Table 1. Optimization of Rhodium Catalyzed Coupling of phenylhydrazine with Cyclohexylallene

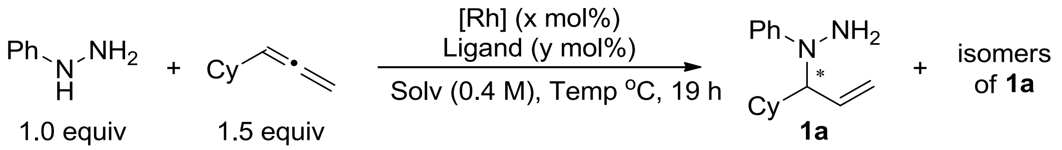

Entries	[Rh] (x)	Ligand (y)	Solv	Temp	Isomers of 1a	yield[a]	*ee*[b]
1	$[Rh(COD)Cl]_2$ (1.25)	(*S*,*S*)-Chiraphos (5.0)	DCE	80	n.d	28	21
2	$[Rh(COD)Cl]_2$ (1.25)	(*R*)-H8-Binap (5.0)	DCE	80	n.d	65	53
3	$[Rh(COD)Cl]_2$ (1.25)	**L1** (5.0)	DCE	50	n.d	9	-
4	$[Rh(COD)Cl]_2$ (1.25)	**L1** (5.0)	DCE	40	n.d	0	-
5	$[Rh(COD)_2]BF_4$ (2.5)	**L1** (5.0)	DCE	80	n.d	27	64
6	$[Rh(ndb)Cl]_2$ (1.25)	**L1** (5.0)	DCE	80	n.d	85	80
7	[Rh(ethylene)]acac (2.5)	**L1** (5.0)	DCE	80	n.d	50	81
8	$[Rh(COD)Cl]_2$ (1.25)	**L1** (5.0)	THF	80	n.d	0	-
9	$[Rh(COD)Cl]_2$ (1.25)	**L1** (5.0)	toluene	80	n.d	0	-
10	$[Rh(COD)Cl]_2$ (1.25)	**L1** (2.5)	DCE	80	n.d	96	79
11	$[Rh(COD)Cl]_2$ (1.25)	**L1** (7.5)	DCE	80	n.d	81	82

[a] Isolated yield; [b] Determined by chiral HPLC.

Supplementary Methods

General Information

FCC (Flash Column Chromatography) was accomplished using MACHEREY-NAGEL silica gel 60® (230-400 mesh). **TLC** (Thin Layer Chromatography) was performed on aluminum plates pre-coated with silica gel (MERCK, $60F_{254}$), which were visualized by UV fluorescence (λ_{max} = 254 nm) and/or by staining with 1% w/v $KMnO_4$ in 0.5 M aqueous K_2CO_3. **NMR** (Nuclear Magnetic Resonance) spectra were acquired on a BRUKER Avance spectrometer (300, 400, or 500 MHz and 100.6 MHz for ^{1}H and ^{13}C respectively). All ^{1}H NMR spectra are reported in parts per million (ppm) downfield of TMS and were measured relative to the signals at 7.26 ppm ($CHCl_3$) or 7.16 ppm (C_6D_6). All ^{13}C NMR spectra were reported in ppm relative to residual $CHCl_3$ (77.16 ppm) or C_6D_6 (128.06 ppm) and were obtained with ^{1}H-decoupling. Data for ^{1}H NMR are described as following: chemical shift (δ in ppm), multiplicity (s, singlet; d, doublet; t, triplet; q, quartet; quin, quintet; sx, sextet; m, multiplet; app, apparent; br, broad signal), coupling constant (Hz), integration. Data for ^{13}C NMR spectra are described in terms of chemical shift (δ in ppm). **HRMS** (High resolution mass spectra) were obtained on a FINNIGAN MAT 8200 instrument (CI/NH_3: 110 eV; EI: 70 eV). **Chiral HPLC** was performed on a MERCK HITACHI HPLC apparatus (pump: L-7100, UV detector: D-7400, oven: L-7360; columns: AD-H, AD-3, OD-3, OJ-H, L-C2, L-C3, AD-3R, OD-3R, OJ-R, and OJ-3R 15-25 cm 4.6 cm, DAICEL). The **Optical Rotation** of chiral compounds was determined on a PERKIN-ELMER PE 241 apparatus and transformed for a given temperature according to the following formula:

Supplementary Equation 1: $$[\alpha]_D^T = \frac{\alpha \cdot 100}{c \cdot d}$$

α: measured value for optical rotation; *c*: concentration in g/100 ml; *d*: length of the cuvette in dm; T: temperature in °C.

Materials

1,2-Dichloroethane (DCE) was freshly distilled over CaH_2 and degassed by three Freeze-Pump-Thaw cycles prior to use. Solvents employed for work-up and column chromatography were purchased in technical grade quality and distilled by rotary evaporator before use. Aryl hydrazines, allenes, ketones and aldehydes, if commercially available, were purchased from Sigma-Aldrich, ABCR, Alfa Aesar and used without further purification. If the aryl hydrazines were obtained as their HCl salt form, the corresponding

aryl hydrazines were liberated according to the literature procedure.[1] Propa-1,2-dien-1-ylcycloheptane,[2] hexa-4,5-dien-1-ylbenzene,[3] 2-(hexa-4,5-dien-1-yl)isoindoline-1,3-dione,[4] methyl hepta-5,6-dienoate[4] and *tert*-butyl(hepta-5,6-dien-1-yloxy)dimethylsilane[4] were synthesized according to literatures. The ligands and $[Rh(COD)Cl]_2$ were purchased from Sigma-Aldrich, ABCR, Alfa Aesar and used without further purification.

Synthesis and Characterization of *N*-Allylic Aryl Hydrazines (1a-l)

1 (*S*)-1-(1-cyclohexylallyl)-1-phenylhydrazine **(1a)**

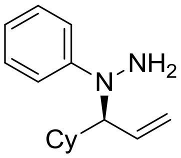

The reaction was performed with cyclohexylallene (109 μl, 91.6 mg, 0.75 mmol) and phenylhydrazine (49.3 μl, 54.1 mg, 0.5 mmol) using ligand **L1** at 80 °C. The crude product was purified by FCC on silica gel (EA/CH = 1/30, R_f = 0.29) to afford the product as a yellow oil (106.8 mg, 93 %).

^{1}H NMR (300MHz, $CDCl_3$) δ = 7.21 - 7.10 (m, 2 H), 6.88 (d, *J* = 8.1 Hz, 2 H), 6.65 (t, *J* = 7.3 Hz, 1 H), 5.73 (ddd, *J* = 17.6, 10.2, 7.8 Hz, 1 H), 4.14 - 5.92 (m, 2 H), 3.76 (m, 1 H), 1.87 - 1.52 (m, 6 H), 1.29 - 1.01 (m, 3 H), 0.98 - 0.79 (m, 2 H); **^{13}C NMR** (100.6MHz, $CDCl_3$) δ = 152.2, 134.1, 128.9, 117.8, 117.6, 113.0, 68.4, 38.2, 30.5, 30.5, 26.6, 26.1, 26.0; **HRMS-APCI** (MeOH, m/z): $[M+H]^+$ calcd for $C_{15}H_{23}N_2$, 231.1861; found, 231.1849; **HPLC** (CHIRALCEL® AD-H, *n*-heptane / ^{i}PrOH = 90:10, 1 mL/min) t_R = 5.45 min (minor), t_R = 9.18 min (major), 85% *ee* (***S***); $[\alpha]_D^{25}$ = + 79.00 (c = 1.360, $CDCl_3$).

Typical procedure for recrystallization (recrystallization of **1a** with tosylic acid): To **1a** was added $EtOH/Et_2O$ and toluene sulfonic acid (1.0 equiv) and the solution was recrystallized at -20 °C to obtain the enantiomerically enriched product as a tosylic salt form. The solid was filtered off and washed with cold Et_2O, then dissolved in dichloromethane before a solution of soldium hydroxide (25%) was added. The organic phase was separated after strong shaking, the water phase was extracted with dichloromethane (10 ml x 2). The combined organic phase was dried over magnesium sulfate, filtered and concentrated to give the enenatiomeric enrich product (66.2 mg, 62% yield; 95% *ee*).

2 (*S*)-1-(1-cyclohexylallyl)-1-(m-tolyl)hydrazine **(1b)**

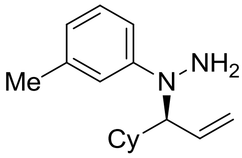

The reaction was performed with cyclohexylallene (218 μl, 183.2 mg, 1.50 mmol) and *m*-tolylhydrazine (122.2 mg, 1.0 mmol) using ligand **L1** at 80 °C, the catalyst/ligand and solvent loading were also doubled accordingly. The crude product was purified by FCC on silica gel (EA/CH = 1/30, R_f = 0.29) to afford the product as a yellow oil (213.6 mg, 93 %).

^{1}H NMR (400MHz, $CDCl_3$) δ = 7. 14 - 7.10 (m, 1 H), 6.80 - 6.75 (m, 2 H), 6.58 - 6.57 (m, 1 H), 5.81 (ddd, *J* = 17.5, 10.2, 7.7 Hz, 1 H), 5.20 - 5.16 (m, 1 H), 5.10 - 5.05 (m, 1 H), 3.85 - 3.80 (m, 1 H), 3.33 (br. s., 1 H), 2.32 (s, 3H), 1.88 - 1.66 (m, 6 H), 1.30 - 1.16 (m, 3 H), 1.02 - 0.94 (m, 2 H); **^{13}C NMR** (100.6MHz, $CDCl_3$) δ = 152.3, 138.7, 134.3, 128.8, 118.6, 117.8, 113.8, 110.3, 68.5, 38.3, 30.6, 30.6, 26.6, 26.2, 26.1, 21.9; **HRMS-APCI** (MeOH, m/z): $[M+H]^+$ calcd for $C_{16}H_{24}N_2$, 245.3831; found, 245.2016. **HPLC** (CHIRALCEL® OJ-3, *n*-heptane / ^{i}PrOH = 97:3, 1 mL/min) t_R = 7.41 min (minor), t_R = 8.70 min (major), 77% *ee* (***S***); $[\alpha]_D^{25}$ = + 79.60 (c = 1.350, $CDCl_3$).

Recrystallization of **1b** adapted the typical procedure using MeOH/H_2O/Et_2O to obtain the enenatiomeric enrich product(110.9 mg, 59% yield; 99% *ee*).

3 (*S*)-1-(1-cyclohexylallyl)-1-(4-fluorophenyl)hydrazine **(1c)**

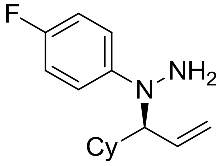

The reaction was performed with cyclohexylallene (218 μl, 183.2 mg, 1.50 mmol) and *p*-fluorophenylhydrazine (126.2 mg, 1.0 mmol) using ligand **L1** at 80 °C, the catalyst/ligand and solvent loading were also doubled accordingly. The crude product was purified by FCC on silica gel, (EA/CH = 1/14, R_f = 0.32) to afford the product as a yellow oil (194.2 mg, 78 %).

^{1}H NMR (400MHz, $CDCl_3$) δ = 6.93 (s, 2 H), 6.92 (s, 2 H), 5.75 (ddd, *J* = 14.0, 8.4, 6.3 Hz, 1 H), 5.21 - 5.18 (m, 1 H), 5.05 - 5.01 (m, 1 H), 3.71 - 3.68 (m, 1 H), 3.36 (br. s., 1 H), 1.95 - 1.92 (m, 1 H), 1.82 - 1.74 (m, 4 H), 1.71 - 1.67 (m, 1 H), 1.32 - 1.14 (m, 3 H), 1.03 - 0.90 (m, 2 H); **^{13}C NMR** (100.6MHz, $CDCl_3$) δ = 156.1 (d, $J_{C\text{-}F}$ = 189.1 Hz), 148.8, 133.4, 118.5, 115.3, 115.1, 70.1, 38.2, 30.6, 26.6, 26.1, 26.1; **HRMS-ESI** (MeOH, m/z): $[M+H]^+$ calcd for $C_{15}H_{22}N_2F$, 249.1767; found, 249.1768. **HPLC** (CHIRALCEL® OJ-H, *n*-heptane / ^{i}PrOH = 90:10, 1 mL/min) t_R = 7.02 min (minor), t_R = 7.98 min (major), 78% *ee* (***S***); $[\alpha]_D^{25}$ = + 62.60 (c = 0.800, $CDCl_3$).

Recrystallization of **1c** adapted the typical procedure using MeOH/Et_2O to obtain the enenatiomeric enrich product(153.3 mg, 69% yield; 95% *ee*).

4 (*S*)-1-(4-chlorophenyl)-1-(1-cyclohexylallyl)hydrazine **(1d)**

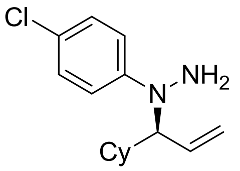

The reaction was performed with cyclohexylallene (109 μl, 91.6 mg, 0.75 mmol), *p*-chlorophenylhydrazine (71.3 mg, 0.5 mmol), $Rh(COD)Cl]_2$ (2 mol%) using ligand **L1** (8 mol%) at 80 °C. The crude product was purified by FCC on silica gel, (EA/CH = 1/14, R_f = 0.35) to afford the product as a yellow oil (89.6 mg, 87 %).

^{1}H NMR (400MHz, $CDCl_3$) δ = 7.17 - 7.13 (m, 2 H), 6.92 - 6.88 (m, 2 H), 5.78 (ddd, *J* = 17.3, 10.5, 7.6 Hz, 1 H), 5.20 (ddd, *J* = 10.5, 1.7, 1.0 Hz, 1 H), 5.06 (ddd, *J* = 17.3, 1.7, 1.0 Hz, 1 H), 3.78 (m, 1 H), 3.14 (br. s., 1 H), 1.87 - 1.66 (m, 6 H), 1.32 - 1.13 (m, 3 H), 1.01 - 0.91 (m, 2 H); **^{13}C NMR** (100.6MHz, $CDCl_3$) δ = 150.9, 133.7, 128.7, 122.4, 118.2, 114.5, 68.8, 38.3, 30.6, 30.6, 26.6, 26.1, 26.1; **HRMS-ESI** (MeOH, m/z): $[M+H]^+$ calcd for $C_{15}H_{22}N_2{}^{35}Cl$, 265.1471; found, 265.1474. **HPLC** (CHIRALCEL® AD-H, *n*-heptane / ^{i}PrOH = 80:20, 1 mL/min) t_R = 5.51 min (minor), t_R = 14.29 min (major), 91% *ee* (***S***); $[\boldsymbol{\alpha}]_{\boldsymbol{D}}^{\mathbf{25}}$ = + 69.90 (c = 1.030, $CDCl_3$).

5 (*S*)-1-(4-bromophenyl)-1-(1-cyclohexylallyl)hydrazine **(1e)**

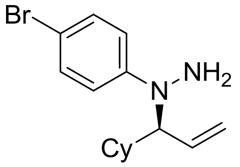

The reaction was performed with cyclohexylallene (109 μl, 91.6 mg, 0.75 mmol) and *p*-bromophenylhydrazine (93.5 mg, 0.5 mmol) using ligand **L1** at 80 °C. The crude product was purified by FCC on silica gel, (EA/CH = 1/14, R_f = 0.37) to afford the product as a yellow oil (124.1 mg, 80 %).

^{1}H NMR (400MHz, $CDCl_3$) δ = 7.30 - 7.26 (m, 2 H), 6.88 - 6.84 (m, 2 H), 5.78 (ddd, *J* = 17.4, 10.6, 7.6 Hz, 1 H), 5.20 (ddd, *J* = 10.6, 1.6, 0.9 Hz, 1 H), 5.06 (ddd, *J* = 17.4, 1.6, 0.9 Hz, 1 H), 3.81 - 3.77 (m, 1 H), 3.32 (br. s., 2 H), 1.86 - 1.66 (m, 6 H), 1.32 - 1.15 (m, 3 H), 1.01 - 0.90 (m, 2 H); **^{13}C NMR** (100.6MHz, $CDCl_3$) δ = 151.3, 133.6, 131.6, 118.2, 114.9, 109.5, 68.6, 38.3, 30.6, 30.5, 26.6, 26.1, 26.0; **HRMS-APCI** (MeOH, m/z): $[M+H]^+$ calcd for $C_{15}H_{22}{}^{79}BrN_2$, 309.09663; found, 309.09670. **HPLC** (CHIRALCEL® AD-H, *n*-heptane / ^{i}PrOH = 80:20, 1 mL/min) t_R = 5.56 min (minor), t_R = 13.94 min (major), 91% *ee* (***S***); $[\boldsymbol{\alpha}]_{\boldsymbol{D}}^{\mathbf{25}}$ = +23.67 (c = 0.490, $CHCl_3$).

The reaction using ligand **L2** under the same condition gave (*R*)-1-(4-bromophenyl)-1-(1-cyclohexylallyl)hydrazine (124.1 mg, 80 %). 91% *ee* (***R***); $[\alpha]_D^{25}$ = -34.35 (c = 0.46, $CHCl_3$).

6 (*R*)-1-(1-cyclohexylallyl)-1-(4-(trifluoromethyl)phenyl)hydrazine **(1f)**

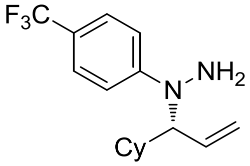

The reaction was performed with cyclohexylallene (109 µl, 91.6 mg, 0.75 mmol) and *p*-trifluoromethylphenylhydrazine (88.1 mg, 0.5 mmol), Rh(COD)Cl]$_2$ (2 mol%) using ligand **L2** (8 mol%) at 80 °C. The crude product was purified by FCC on silica gel (EA/CH = 1/29, R_f = 0.23) to afford the product as a yellow oil (101.4 mg, 68 %).

^{1}H NMR (400MHz, $CDCl_3$) δ = 7.46 - 7.42 (m, 2 H), 7.04 - 7.00 (m, 2 H), 5.84 (ddd, *J* = 17.5, 10.5, 7.4 Hz, 1 H), 5.23 (ddd, *J* = 10.5, 1.5, 0.9 Hz, 1 H), 5.12 (ddd, *J* = 17.5, 1.5, 0.9 Hz, 1 H), 3.95 (ddt, *J* = 9.6, 7.4, 0.9 Hz, 1 H), 3.45 (br. s., 2 H), 1.87 - 1.68 (m, 6 H), 1.31 - 1.16 (m, 3 H), 1.05 - 0.93 (m, 2 H); **^{13}C NMR** (100.6MHz, $CDCl_3$) δ = 154.3, 133.7, 126.3 (q, $J_{C\text{-}F}$= 3.8 Hz), 125.0 (q, $J_{C\text{-}F}$= 270.6 Hz), 118.7 (d, $J_{C\text{-}F}$= 32.2 Hz), 118.2, 111.7, 67.6, 38.3, 30.5, 26.5, 26.1, 26.0; **HRMS-APCI** (MeOH, m/z): $[M+H]^+$ calcd for $C_{16}H_{22}N_2F_3$, 299.1735; found, 299.1732. **HPLC** (CHIRALCEL® AD-H, *n*-heptane / ^{i}PrOH = 85:15, 1 mL/min) t_R = 5.46 min (major), t_R = 9.96 min (minor), 85% *ee* (***R***); $[\alpha]_D^{25}$ = - 64.40 (c = 1.000, $CHCl_3$).

7 (*S*)-1-(1-cyclohexylallyl)-1-(2,5-difluorophenyl)hydrazine **(1g)**

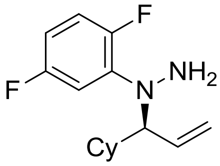

The reaction was performed with cyclohexylallene (109 µl, 91.6 mg, 0.75 mmol) and 2,5-difluorophenylhydrazine (72.1 mg, 0.5 mmol) using ligand **L1** at 100 °C. The crude product was purified by FCC on silica gel (EA/CH = 1/14, R_f = 0.29) to afford the product as a yellow oil (92.8 mg, 70 %).

^{1}H NMR (400MHz, $CDCl_3$) δ = 6.95 - 6.86 (m, 2 H), 6.53 - 6.47 (m, 1 H), 5.76 (dddd, *J* = 17.4, 10.4, 8.1 Hz, $J_{F\text{-}H}$ = 1.6 Hz, 1 H), 5.22 (ddd, *J* = 10.4, 1.7, 0.6 Hz, 1 H), 4.98 (ddd, *J* = 17.4, 1.7, 0.9 Hz, 1 H), 3.70 - 3.65 (m, 1 H), 3.33 (br. s., 1 H), 2.07 - 2.00 (m, 1 H), 1.84 - 1.65 (m, 5 H), 1.33 - 1.07 (m, 4 H), 0.98 -

0.87 (m, 1 H); **^{13}C NMR** (100.6MHz, $CDCl_3$) δ = 158.9 (dd, $J_{C\text{-}F}$ = 240.4, 1.9 Hz), 149.3 (dd, $J_{C\text{-}F}$ = 239.0, 2.7 Hz), 142.4 (t, $J_{C\text{-}F}$ = 9.4 Hz), 133.4, 119.4, 116.4 (dd, $J_{C\text{-}F}$ = 24.4, 10.0 Hz), 107.1 (dd, $J_{C\text{-}F}$ = 27.3, 3.3 Hz), 106.9 (dd, $J_{C\text{-}F}$ = 24.4, 8.2 Hz), 71.2 (d, $J_{C\text{-}F}$ = 7.2 Hz), 38.1, 30.5, 30.1, 26.6, 26.2, 26.1; **^{19}F NMR** (235.3MHz, $CDCl_3$) δ = -118.18 (dddd, *J* = 16.1, 10.9, 7.3, 5.4 Hz, 1 F), -127.16 (m, 1 F); **HRMS-APCI** (MeOH, m/z): $[M+H]^+$ calcd for $C_{15}H_{21}N_2F_2$, 267.1673; found, 267.1671. **HPLC** (CHIRALCEL® OJ-H, *n*-heptane / ^{i}PrOH = 97:3, 1 mL/min) t_R = 6.48 min (major), t_R = 7.88 min (minor), 85% *ee* (***S***); $[\alpha]_D^{25}$ = - 6.20 (c = 0.970, $CHCl_3$).

8 (*R*)-1-(4-bromophenyl)-1-(1-cycloheptylallyl)hydrazine **(1h)**

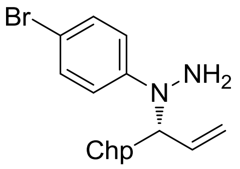

The reaction was performed with cycloheptylallene (117 μl, 102.2 mg, 0.75 mmol) and 4-bromophenylhydrazine (94 mg, 0.5 mmol) using ligand **L2** at 80 °C. The crude product was purified by FCC on silica gel (EA/CH = 1/30, R_f = 0.31) to afford the product as a yellow oil (133.8 mg, 83 %).

^{1}H NMR (400MHz, $CDCl_3$) δ = 7.31 - 7.27 (m, 2 H), 6.89 - 6.85 (m, 2 H), 5.77 (ddd, *J* = 17.4, 10.5, 7.6 Hz, 1 H), 5.20 (ddd, *J* = 10.5, 1.6, 0.9 Hz, 1 H), 5.06 (ddd, *J* = 17.4, 1.6, 0.9 Hz, 1 H), 3.82 (dd, *J* = 10.5, 7.6 Hz, 1 H), 3.30 (br. s., 2 H), 2.06 - 1.97 (m, 1 H), 1.87 - 1.76 (m, 2 H), 1.73 - 1.41 (m, 8 H), 1.33 - 1.18 (m, 2 H); **^{13}C NMR** (100.6MHz, $CDCl_3$) δ = 151.2, 134.1, 131.6, 118.2, 114.9, 109.5, 68.1, 39.5, 31.4, 30.6, 28.7, 28.5, 26.6, 26.6; **HRMS-APCI** (MeOH, m/z): $[M+H]^+$ calcd for $C_{16}H_{24}{}^{79}BrN_2$, 323.1117; found, 323.1118. **HPLC** (CHIRALCEL® AD-3, *n*-heptane / ^{i}PrOH = 90:10, 1 mL/min) t_R = 4.10 min (major), t_R = 10.71 min (minor), 88% *ee* (***R***); $[\alpha]_D^{25}$ = - 44.60 (c = 1.000, $CHCl_3$).

9 (*S*)-1-(4-bromophenyl)-1-(6-phenylhex-1-en-3-yl)hydrazine **(1i)**

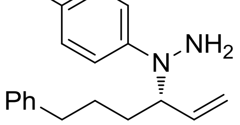

The reaction was performed with penta-3,4-dien-1-ylbenzene (128 μl, 118.7 mg, 0.75 mmol) and 4-bromophenylhydrazine (94 mg, 0.5 mmol) using ligand **L2** at 80 °C. The crude product was purified by FCC on silica gel (EA/CH = 1/14, R_f = 0.21) to afford the product as a yellow oil (159 mg, 92 %).

^{1}H NMR (400MHz, $CDCl_3$) δ = 7.32 - 7.26 (m, 4 H), 7.21 - 7.15 (m, 3 H), 6.94 - 6.90 (m, 2 H), 5.74 (ddd, *J* = 17.4, 10.6, 5.8 Hz, 1 H), 5.21 (ddd, *J* = 10.6, 1.4, 1.4 Hz, 1 H), 5.10 (ddd, *J* = 17.4, 1.4, 1.4 Hz, 1 H), 4.23 - 4.19 (m, 1 H), 3.46 (br. s., 2 H), 2.72 - 2.60 (m, 2 H), 1.87 - 1.65 (m, 4 H); **^{13}C NMR** (100.6MHz, $CDCl_3$) δ = 142.1, 134.9, 131.7, 128.4, 125.9, 117.7, 115.8, 62.3, 35.7, 29.9, 28.1; **HRMS-APCI** (MeOH, m/z): $[M+H]^+$ calcd for $C_{18}H_{21}{}^{79}BrN_2$, 345.0966; found, 345.0968. **HPLC** (CHIRALCEL® OJ-H, *n*-heptane / ^{i}PrOH = 80:20, 1 mL/min) t_R = 19.73 min (minor), t_R = 36.72 min (major), 85% *ee* (***S***); $[\alpha]_D^{25}$ = - 41.70 (c = 1.240, $CDCl_3$).

10 (*S*)-2-(4-(1-(4-bromophenyl)hydrazinyl)hex-5-en-1-yl)isoindoline-1,3-dione **(1j)**

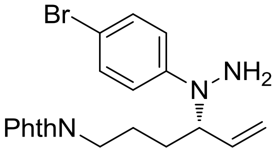

The reaction was performed with 2-(hexa-4,5-dien-1-yl)isoindoline-1,3-dione (170.0 mg, 0.75 mmol) and 4-bromophenylhydrazine (94 mg, 0.5 mmol) using ligand **L2** at 80 °C. The crude product was purified by FCC on silica gel (EA/CH = 2/8, R_f = 0.14) to afford the product as a yellow oil (192.6 mg, 93 %).

^{1}H NMR (500MHz, $CDCl_3$) δ = 7.83 (dd, *J* = 5.4, 3.0 Hz, 2 H), 7.71 (dd, *J* = 5.4, 3.0 Hz, 2 H), 7.29 - 7.26 (m, 2 H), 6.92 - 6.89 (m, 2 H), 5.73 (ddd, *J* = 17.3, 10.6, 5.9 Hz, 1 H), 5.20 (ddd, *J* = 10.6, 1.2, 1.2 Hz, 1 H), 5.11 (ddd, *J* = 17.3, 1.2, 1.2 Hz, 1 H), 4.29 - 4.25 (m, 1 H), 3.74 (t, *J* = 7.0 Hz, 2 H), 3.30 (br. s., 2 H), 1.93 - 1.64 (m, 4 H); **^{13}C NMR** (100.6MHz, $CDCl_3$) δ = 168.4, 150.9, 134.7, 133.9, 132.0, 131.7, 123.2, 117.7, 115.4, 110.2, 61.7, 37.7, 27.8, 25.5; **HRMS-APCI** (MeOH, m/z): $[M+H]^+$ calcd for $C_{20}H_{21}{}^{79}BrN_3O_2$, 414.0817; found, 414.0815. **HPLC** (CHIRALCEL® AD-H, *n*-heptane / ^{i}PrOH = 80:20, 1 mL/min) t_R = 16.65 min (major), t_R = 26.20 min (minor), 79% *ee* (***S***); $[\alpha]_D^{25}$ = - 29.40 (c = 1.010, $CHCl_3$).

11 (*S*)-methyl 5-(1-(4-bromophenyl)hydrazinyl)hept-6-enoate **(1k)**

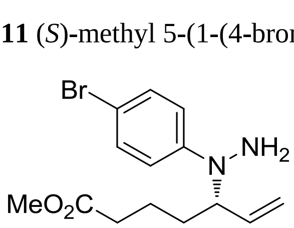

The reaction was performed with hepta-5,6-dienoic acid methyl ester (105.1 mg, 0.75 mmol) and 4-bromophenylhydrazine (94 mg, 0.5 mmol) using ligand **L2** at 80 °C. The crude product was purified by FCC on silica gel (EA/CH = 1/30, R_f = 0.31) to afford the product as a yellow oil (140.6 mg, 86 %).

^{1}H NMR (400MHz, $CDCl_3$) δ = 7.32 - 7.28 (m, 2 H), 6.94 - 6.90 (m, 2 H), 5.74 (ddd, *J* = 17.5, 10.6, 5.6 Hz, 1 H), 5.22 (dt, *J* = 10.6, 1.1 Hz, 1 H), 5.08 (dt, *J* = 17.5, 1.1 Hz, 1 H), 4.25 - 4.19 (m, 1 H), 3.67 (s, 3 H), 3.34 (br. s., 2 H), 2.43 - 2.31 (m, 2 H), 1.91 - 1.60 (m, 4 H); **^{13}C NMR** (100.6MHz, $CDCl_3$) δ = 173.9, 151.0, 134.7, 131.7, 117.5, 115.4, 110.0, 61.7, 51.5, 33.5, 29.9, 21.7; **HRMS-APCI** (MeOH, m/z): $[M+H]^+$ calcd for $C_{14}H_{20}{}^{79}BrN_2O_2$, 327.0708; found, 327.0710. **HPLC** (CHIRALCEL® L-C2, *n*-heptane / ^{i}PrOH = 95:5, 1 mL/min) t_R = 15.31 min (major), t_R = 18.88 min (minor), 84% *ee* (***S***); $[\alpha]_D^{25}$ = - 47.50 (c = 1.060, $CHCl_3$).

12 (*S*)-1-(4-bromophenyl)-1-(7-((tert-butyldimethylsilyl)oxy)hept-1-en-3-yl)hydrazine **(1l)**

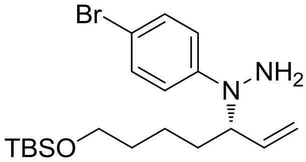

The reaction was performed with *tert*-butyldimethyl(hepta-5,6-dien-1-yloxy)silane (169.8 mg, 0.75 mmol) and 4-bromophenylhydrazine (94 mg, 0.5 mmol) using ligand **L2** at 80 °C. The crude product was purified by FCC on silica gel (EA/CH = 1/15, R_f = 0.22) to afford the product as a yellow oil (154.8 mg, 75 %).

^{1}H NMR (500MHz, $CDCl_3$) δ = 7.31 - 7.28 (m, 2 H), 6.94 - 6.91 (m, 2 H), 5.76 (ddd, *J* = 17.4, 10.6, 5.6 Hz, 1 H), 5.22 (dt, *J* = 10.6, 1.3 Hz, 1 H), 5.12 (dt, *J* = 17.4, 1.3 Hz, 1 H), 4.25 - 4.20 (m, 1 H), 3.62 (t, *J* = 6.4 Hz, 2 H), 3.29 (br. s., 2 H), 1.86 - 1.79 (m, 1 H), 1.71 - 1.63 (m, 1 H), 1.60 - 1.52 (m, 2 H), 1.47 - 1.34 (m, 2 H), 0.89 (s, 9 H), 0.04 (s, 6 H); **^{13}C NMR** (125.8MHz, $CDCl_3$) δ = 151.0, 135.1, 131.6, 117.3, 115.3, 109.8, 62.9, 61.9, 32.6, 30.1, 25.9, 22.7, 18.3, -5.3; **HRMS-APCI** (MeOH, m/z): $[M+H]^+$ calcd for $C_{19}H_{34}{}^{79}BrN_2OSi$, 413.1618; found, 413.1619. **HPLC** (CHIRALCEL® OD-3, *n*-heptane / ^{i}PrOH = 95:5, 1 mL/min) t_R = 3.20 min (minor), t_R = 3.77 min (major), 83% *ee* (***S***); $[\alpha]_D^{25}$ = - 33.30 (c = 1.040, $CHCl_3$).

Determination of absolute configuration

Absolute configuration was determined by comparing the specific rotation the NH_2-cleaved[5] products with literature.[6] Cleavage of the N-N bond of both **1e** and (*R*)-1-(4-bromophenyl)-1-(1-cyclohexylallyl)hydrazine gave the corresponding anline derivatives **1ea**. (*S*)-4-bromo-*N*-(1-cyclohexylallyl)aniline (**1ea**): $[\alpha]_D^{25}$ = -3.08 (c = 0.455, $CHCl_3$). Literature value of (*S*)-4-bromo-*N*-(1-cyclohexylallyl)aniline: 90% *ee*, $[\alpha]_D^{25}$ = - 4.2 (c = 0.356, $CHCl_3$). The absolute

configurations for other allylation products (allylic arylhydrazines and allylic indoles) were assignied by analogy.

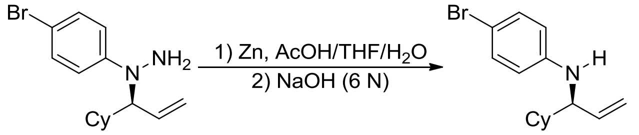

(*S*)-1-(4-bromophenyl)-1-(1-cyclohexylallyl)hydrazine

(*S*)-4-bromo-*N*-(1-cyclohexylallyl)aniline

Observed Specific Rotation:
$[\alpha]_D^{25}$ = - 3.08 (c = 0.455, $CHCl_3$)

Literature value [(*S*)-enantiomer]:
$[\alpha]_D^{25}$ = - 4.2 (c = 0.356, $CHCl_3$)

Synthesis and Characterization of *N*-Allylic Indoles (2a-i)

1 (*S*)-9-(1-cyclohexylallyl)-2,3,4,9-tetrahydro-1*H*-carbazole **(2a)**

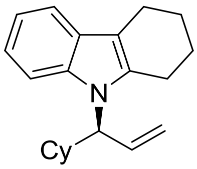

To the crude reaction mixture of **1a** was added cyclohexanone (57 µL, 54 mg, 0.55 mmol). The indolization was proceeded for 3 h at 70 °C. The crude product was purified by FCC on silica gel (CH, R_f = 0.36) to afford the product as a white solid (122.2 mg, 87 %).

^{1}H NMR (300MHz, $CDCl_3$) δ = 7.55 - 7.52 (m, 1 H), 7.43 - 7.40 (m, 1 H), 7.19 - 7.09 (m, 2 H), 6.37 (ddd, *J* = 17.1, 10.3, 7.1 Hz, 1 H), 5.22 (dt, *J* = 10.3, 1.3 Hz, 1 H), 5.10 (dt, *J* = 17.1, 1.3 Hz, 1 H), 4.41 (dd, *J* = 10.3, 7.1 Hz, 1 H), 2.87 - 2.75 (m, 4 H), 2.33 - 2.20 (m, 1 H), 2.13 - 2.09 (m, 1 H), 2.05 - 1.86 (m, 5 H), 1.75 - 1.61 (m, 2 H), 1.45 - 1.31 (m, 1 H), 1.28 - 1.02 (m, 4 H), 0.89 - 0.76 (m, 1 H); **^{13}C NMR** (100.6MHz, $CDCl_3$) δ = 135.5, 135.5, 135.2, 127.9, 120.1, 118.3, 117.7, 117.6, 110.9, 109.4, 63.7, 40.0, 31.5, 29.9, 26.0, 25.9, 23.6, 23.6, 23.1, 21.1; **HRMS-APCI** (MeOH, m/z): $[M+H]^+$ calcd for $C_{21}H_{28}N$, 294.2222; found, 294.2222. **m.p.**: 67 - 68 °C; **HPLC** (CHIRALCEL® OD-3R, MeCN / H_2O = 70:30, 1 mL/min) t_R = 36.03 min (major), t_R = 38.86 min (minor), 82% *ee* (***S***); $[\boldsymbol{\alpha}]_{\boldsymbol{D}}^{\mathbf{25}}$ = - 55.30 (c = 0.975, $CHCl_3$).

2 (*S*)-9-(1-cyclohexylallyl)-6-fluoro-2,3,4,9-tetrahydro-1*H*-carbazole **(2b)**

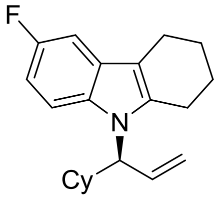

To the crude reaction mixture of **1c** was added cyclohexanone (57 µL, 54 mg, 0.55 mmol). The indolization was proceeded for 18 h at 70 °C. The crude product was purified by FCC on silica gel (CH, R_f = 0.45) to afford the product as a yellowish oil (141.0 mg, 90 %).

^{1}H NMR (400MHz, $CDCl_3$) δ = 7.28 (ddd, *J* = 9.1, 0.3 Hz, J_{H-F} = 4.3 Hz, 1 H), 7.15 (ddd, *J* = 2.6, 0.3 Hz, J_{H-F} = 9.5 Hz, 1 H), 6.87 (ddd, *J* = 9.1, 2.6 Hz, J_{H-F} = 9.1 Hz, 1 H), 6.31 (ddd, *J* = 17.1, 10.3, 7.0 Hz, 1 H), 5.22 (dt, *J* = 10.3, 1.4 Hz, 1 H), 5.07 (dt, *J* = 17.1, 1.4 Hz, 1 H), 4.38 - 4.34 (m, 1 H), 2.80 - 2.68 (m, 4 H), 2.25 - 2.15 (m, 1 H), 2.12 - 2.04 (m, 1 H), 2.01 - 1.95 (m, 2 H), 1.92 - 1.83 (m, 3 H), 1.74 - 1.67 (m, 1 H), 1.66 - 1.59 (m, 1 H), 1.41 - 1.29 (m, 1 H), 1.25 - 1.01 (m, 4 H), 0.84 - 0.74 (m, 1 H); **^{13}C NMR** (100.6MHz,

$CDCl_3$) δ = 157.4 (d, $J_{C\text{-}F}$ = 233.5 Hz), 137.4, 135.3, 131.7, 128.3 (d, $J_{C\text{-}F}$ = 9.1 Hz), 117.7, 111.3 (d, $J_{C\text{-}F}$ = 9.1 Hz), 109.5 (d, $J_{C\text{-}F}$ = 4.0 Hz), 108.0 (d, $J_{C\text{-}F}$ = 25.2 Hz), 102.7 (d, $J_{C\text{-}F}$ = 23.1 Hz), 63.7, 40.1, 31.5, 29.9, 26.3, 26.0, 25.8, 23.6, 23.5, 23.0, 21.1; **HRMS-APCI** (MeOH, m/z): $[M+H]^+$ calcd for $C_{21}H_{27}FN$, 312.2127; found, 312.2129. **HPLC** (CHIRALCEL® AD-3R, MeCN / H_2O = 65:35, 0.5 mL/min) t_R = 31.61 min (major), t_R = 35.24 min (minor), 85% *ee* (***S***); $[\alpha]_D^{25}$ = - 61.55 (c = 1.035, $CHCl_3$).

3 (*S*)-6-bromo-9-(1-cyclohexylallyl)-2,3,4,9-tetrahydro-1*H*-carbazole **(2c)**

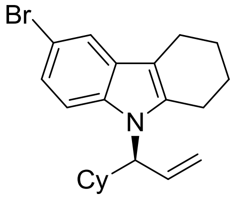

To the crude reaction mixture of **1e** was added cyclohexanone (57 μL, 54 mg, 0.55 mmol). The indolization was proceeded for 18 h at 70 °C. The crude product was purified by FCC on silica gel (CH, R_f = 0.38) to afford the product as a yellowish oil (123.0 mg, 72 %).

^{1}H NMR (400MHz, $CDCl_3$) δ = 7.57 (s, 1 H), 7.20 - 7.13 (m, 2 H), 6.25 (ddd, *J* = 17.1, 10.4, 6.9 Hz, 1 H), 5.18 (dd, *J* = 10.4, 1.0 Hz, 1 H), 5.00 (dd, *J* = 17.1, 1.0 Hz, 1 H), 4.31 (dd, *J* = 10.4, 6.9 Hz, 1 H), 2.76 - 2.63 (m, 4 H), 2.19 - 2.09 (m, 1 H), 2.05 - 2.02 (m, 1 H), 1.96 - 1.90 (m, 2 H), 1.87 - 1.80 (m, 3 H), 1.68 - 1.65 (m, 1 H), 1.59 - 1.55 (m, 1 H), 1.35 - 1.24 (m, 1 H), 1.19 - 0.96 (m, 4 H), 0.78 - 0.68 (m, 1 H); **^{13}C NMR** (100.6MHz, $CDCl_3$) δ = 137.0, 135.1, 133.8, 129.7, 122.7, 120.4, 117.8, 112.3, 111.7, 109.2, 63.6, 40.1, 31.5, 29.9, 26.3, 26.0, 25.8, 23.5, 23.4, 23.0, 21.0; **HRMS-APCI** (MeOH, m/z): $[M+H]^+$ calcd for $C_{21}H_{27}BrN$, 372.1327; found, 372.1321. $[\alpha]_D^{25}$ = - 53.06 (c = 0.965, $CHCl_3$).

The *ee* value of **2c** was measured via its derivative **2cee**, which was derived from **2c** according to the literature procedure via debromination with ^{t}BuLi and MeOH.[7] The NMR data of **2cee** is consistent with **2a**. **HPLC** (CHIRALCEL® OD-3R, MeCN / H_2O = 70:30, 0.5 mL/min) t_R = 27.96 min (major), t_R = 30.00 min (minor), 89% *ee* (***S***); $[\alpha]_D^{25}$ = - 59.85 (c = 0.660, $CHCl_3$).

4 (*R*)-6-bromo-9-(1-cycloheptylallyl)-2,3,4,9-tetrahydro-1*H*-carbazole **(2d)**

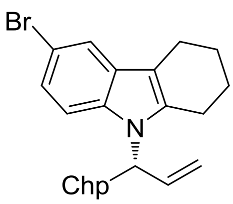

To the crude reaction mixture of **1h** was added cyclohexanone (57 μL, 54 mg, 0.55 mmol). The indolization was proceeded for 18 h at 70 °C. The crude product was purified by FCC on silica gel (CH, R_f = 0.42) to afford the product as a yellowish oil (146.7 mg, 76 %).

^{1}H NMR (400MHz, $CDCl_3$) δ = 7.59 (dd, *J* = 1.9, 0.5 Hz, 1 H), 7.23 (dd, *J* = 8.7, 0.5 Hz, 1 H), 7.17 (dd, *J* = 8.7, 1.9 Hz, 1 H), 6.26 (ddd, *J* = 17.1, 10.2, 7.1 Hz, 1 H), 5.19 (dt, *J* = 10.2, 1.3 Hz, 1 H), 5.04 (dt, *J* = 17.1, 1.3 Hz, 1 H), 4.37 (ddt, *J* = 11.0, 7.1, 1.3, Hz, 1 H), 2.77 - 2.66 (m, 4 H), 2.46 - 2.36 (m, 1 H), 2.04 - 1.92 (m, 3 H), 1.89 - 1.83 (m, 2 H), 1.80 - 1.72 (m, 1 H), 1.66 - 1.46 (m, 6 H), 1.38 - 1.15 (m, 3 H), 1.01 - 0.92 (m, 1 H); **^{13}C NMR** (100.6MHz, $CDCl_3$) δ = 137.0, 135.8, 133.9, 129.7, 122.7, 120.4, 117.9, 112.2, 111.7, 109.3, 63.1, 41.3, 32.1, 30.2, 28.7, 28.5, 26.0, 26.0, 23.5, 23.4, 22.9, 21.0; **HRMS-APCI** (MeOH, m/z): $[M+H]^+$ calcd for $C_{22}H_{29}{}^{79}BrN$, 386.1478; found, 386.1478. $[\alpha]_D^{25}$ = + 44.04 (c = 0.990, $CHCl_3$).

The *ee* value of **2d** was measured via its derivative **2dee**, which was derived from **2d** according to the modified literature procedure.[8] In a one neck flask was added **2d** (47.8 mg, 0.1237 mmol), 4-hydroxyphenylboronic acid pinacol ester (27.5 mg, 0.1237 mmol, 1.0 equiv), $Pd(PPh_3)_2Cl_2$ (4.34 mg, 0.006185 mmol, 5 mol%), XPhos (5.90 mg, 0.01237 mmol, 10 mol%), K_3PO_4 (78.77 mg, 0.3711 mmol, 3.0 equiv) and a 2/1 mixture of dioxane/H_2O (3.2 ml) under argon. After stirred the reaction mixture at 90 °C overnight, water and saturated solution of NH_4Cl were added. Layers were separated and the aqueous were extracted with ethyl acetate. Combined organic extracts were dried over $MgSO_4$, filtered and concentrated by rotary evaporation. The crude product was purified by FCC on silica gel (EA/PE = 1/9, R_f = 0.24) to afford the desired product **2dee** as a white solid (40.6 mg, 82 %).

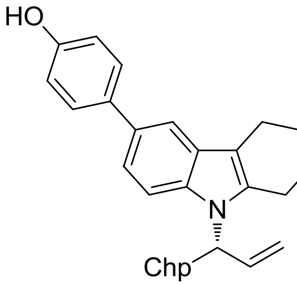

2dee: ^{1}H NMR (400MHz, $CDCl_3$) δ = 7.60 - 7.59 (m, 1 H), 7.55 - 7.50 (m, 2 H), 7.38 - 7.35 (m, 1 H), 7.29 - 7.26 (m, 1 H), 6.91 - 6.87 (m, 2 H), 6.29 (ddd, *J* = 17.1, 10.1, 7.3 Hz , 1 H), 5.16 (dt, *J* = 17.1, 1.2 Hz, 1 H), 5.06 (dt, *J* = 17.1, 1.2 Hz, 1 H), 4.66 (s, 1 H), 4.38 (m, 1 H), 2.78 - 2.70 (m, 4 H), 2.50 - 2.41 (m, 1 H), 2.03 - 1.92 (m, 3 H), 1.89 - 1.83 (m, 2 H), 1.79 - 1.72 (m, 1 H), 1.65 - 1.45 (m, 5 H), 1.38 - 1.24 (m, 4 H), 1.05 - 0.96 (m, 1 H); **^{13}C NMR** (100.6MHz, $CDCl_3$) δ = 154.1, 136.2, 135.9, 134.5, 131.5, 128.4, 128.4,

119.7, 117.7, 115.8, 115.6, 115.4, 110.9, 109.9, 63.2, 41.4, 32.2, 30.3, 28.8, 28.5, 26.1, 26.0, 23.7, 23.6, 23.1, 21.2; **HRMS-APCI** (MeOH, m/z): $[M+H]^+$ calcd for calcd for $C_{28}H_{34}NO$, 400.2635; found, 400.2636. **m.p.**: 70 - 72 °C; **HPLC** (CHIRALCEL® AD-3, *n*-heptane / EtOH = 99:1, 1 mL/min) t_R = 29.15 min (minor), t_R = 34.70 min (major), 87% *ee* (***R***) $[\alpha]_D^{25}$ = + 52.96 (c = 0.995, $CHCl_3$).

5 (*S*)-2-(4-(6-bromo-3,4-dihydro-1*H*-carbazol-9(2*H*)-yl)hex-5-en-1-yl)isoindoline-1,3-dione **(2e)**

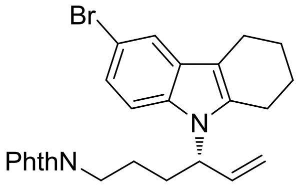

To the crude reaction mixture of **1g** was added cyclohexanone (57 µL, 54 mg, 0.55 mmol). The indolization was proceeded for 18 h at 70 °C. The crude product was purified by FCC on silica gel (EA/CH = 1/15, R_f = 0.36) to afford the product as a white solid (88.5 mg, 51 %).

^{1}H NMR (400MHz, C_6D_6) δ = 7.70 (dd, *J* = 2.0, 0.4 Hz, 1 H), 7.43 - 7.39 (m, 2 H), 7.19 (dd, *J* = 8.7, 2.0 Hz, 1 H), 6.96 (dd, *J* = 8.7, 0.4 Hz, 1 H), 6.93 - 6.88 (m, 2 H), 5.69 (ddd, *J* = 17.2, 10.6, 4.6 Hz, 1 H), 4.86 (ddd, *J* = 10.6, 2.0, 1.1 Hz, 1 H), 4.75 (ddd, *J* = 17.2, 2.0, 1.1 Hz, 1 H), 4.48 - 4.42 (m, 1 H), 3.36 - 3.25 (m, 2 H), 2.50 - 2.42 (m, 3 H), 2.34 - 2.27 (m, 1 H), 2.01 - 1.91 (m, 1 H), 1.74 - 1.53 (m, 5 H), 1.40 - 1.20 (m, 2 H); **^{13}C NMR** (100.6MHz, C_6D_6) δ = 168.3, 137.7, 137.2, 134.7, 133.9, 132.8, 130.9, 123.9, 123.3, 121.5, 116.2, 113.0, 113.0, 110.4, 56.9, 37.6, 30.4, 25.9, 24.0, 23.8, 23.6, 21.5; **HRMS-APCI** (MeOH, m/z): $[M+H]^+$ calcd for $C_{26}H_{26}{}^{79}BrN_2O_2$, 477.1178; found, 477.1176. **m.p.**: 50 -51 °C; **HPLC** (CHIRALCEL® AD-3, *n*-heptane / EtOH = 98:2, 0.5 mL/min) t_R = 17.38 min (major), t_R = 18.93 min (minor), 76% *ee* (***S***); $[\alpha]_D^{25}$ = - 1.59 (c = 1.005, $CHCl_3$).

6 (*S*)-methyl 5-(6-bromo-3,4-dihydro-1*H*-carbazol-9(2*H*)-yl)hept-6-enoate **(2f)**

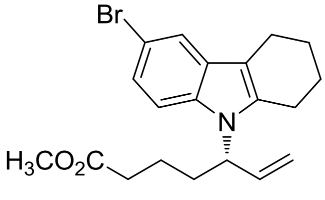

To the crude reaction mixture of **1k** was added cyclohexanone (57 µL, 54 mg, 0.55 mmol). The indolization was proceeded for 18 h at 70 °C. The crude product was purified by FCC on silica gel (EA/CH = 1/30, R_f = 0.14) to afford the product as a yellowish oil (143.9 mg, 74 %).

^{1}H NMR (400MHz, $CDCl_3$) δ = 7.57 (dd, *J* = 1.7, 0.8 Hz, 1 H), 7.16 (dd, *J* = 8.7, 0.8 Hz, 1 H), 7.13 (dd, *J* = 8.7, 1.7 Hz, 1 H), 6.09 (ddd, *J* = 17.2, 10.5, 4.7 Hz, 1 H), 5.19 (ddd, *J* = 10.5, 2.0, 0.9 Hz, 1 H), 5.02 (ddd, *J* = 17.2, 2.0, 0.9 Hz, 1 H), 4.82 - 4.76 (m, 1 H), 3.62 (s, 3 H), 2.73 - 2.64 (m, 4 H), 2.24 (dt, *J* = 7.3, 0.9 Hz, 2 H), 2.20 - 2.11 (m, 1 H), 2.08 - 1.99 (m, 1 H), 1.96 - 1.89 (m, 2 H), 1.88 - 1.81 (m, 2 H), 1.65 - 1.53 (m, 1 H), 1.38 - 1.27 (m, 1 H); **^{13}C NMR** (100.6MHz, $CDCl_3$) δ = 173.5, 136.9, 136.9, 133.8, 129.9, 122.9, 120.5, 116.2, 112.2, 111.9, 109.6, 56.7, 51.5, 33.4, 32.2, 23.3, 23.3, 22.9, 21.9, 20.9; **HRMS-APCI** (MeOH, m/z): $[M+H]^+$ calcd for $C_{20}H_{25}{}^{79}BrNO_2$, 390.1063; found, 390.1063. **HPLC** (CHIRALCEL® OD-3, *n*-heptane / ^{i}PrOH = 200:1, 1 mL/min) t_R = 9.66 min (major), t_R = 11.18 min (minor), 81% *ee* (***S***); $[\alpha]_D^{25}$ = + 15.51 (c = 0.980, $CHCl_3$).

7 (*S*)-5-bromo-1-(1-cyclohexylallyl)-3-phenyl-1*H*-indole **(2g)**

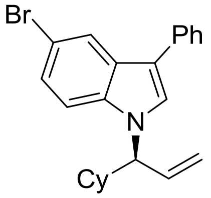

To the crude reaction mixture of **1a** was added phenylacetaldehyde (64 µl, 62 mg, 0.55 mmol). The indolization was proceeded for 18 h at 70 °C. The crude product was purified by FCC on silica gel (CH = 1/30, R_f = 0.29) to afford the product as a yellowish oil (160.0 mg, 81 %).

^{1}H NMR (400MHz, $CDCl_3$) δ = 8.05 - 8.05 (m, 1 H), 7.64 - 7.61 (m, 2 H), 7.47 - 7.43 (m, 2 H), 7.34 - 7.27 (m, 4 H), 6.12 (ddd, *J* = 17.1, 10.3, 7.4 Hz, 1 H), 5.24 (ddd, *J* = 10.3, 1.1 Hz, 1 H), 5.16 (ddd, *J* = 17.1, 1.1 Hz, 1 H), 4.52 - 4.48 (m, 1 H), 2.00 - 1.90 (m, 2 H), 1.84 - 1.77 (m, 1 H), 1.70 - 1.61 (m, 2 H), 1.41 - 1.35 (m, 1 H), 1.34 - 1.20 (m, 1 H), 1.19 - 1.02 (m, 3 H), 0.95 - 0.85 (m, 1 H); **^{13}C NMR** (100.6MHz, $CDCl_3$) δ = 135.8, 135.5, 135.0, 128.8, 127.7, 127.4, 126.1, 124.6, 124.2, 122.5, 118.1, 117.0, 113.2, 111.5, 64.7, 41.9, 30.6, 29.8, 26.2, 25.9, 25.8; **HRMS-APCI** (MeOH, m/z): $[M+H]^+$ calcd for $C_{23}H_{25}{}^{79}BrN$, 394.1170; found, 394.1174. $[\alpha]_D^{25}$ = - 34.83 (c = 1.025, $CHCl_3$).

The *ee* value of **2g** was measured via its derivative **2gee**, which was derived from **2g** according to the literature procedure (white solid, 39.4 mg, 76 %).[9]

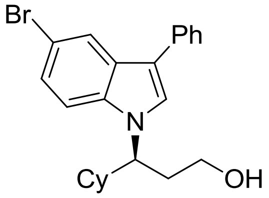

(*S*)-3-(5-bromo-3-phenyl-1*H*-indol-1-yl)-3-cyclohexylpropan-1-ol (**2gee**): **^{1}H NMR** (400MHz, $CDCl_3$) δ = 8.06 - 8.05 (m, 1 H), 7.64 - 7.61 (m, 2 H), 7.47 - 7.43 (m, 2 H), 7.32 - 7.28 (m, 4 H), 4.30 (m, 1 H), 3.52 (ddd, *J* = 10.0, 6.1, 3.8 Hz, 1 H), 3.19 (ddd, *J* = 10.0, 10.0, 4.7 Hz, 1 H), 2.34 - 2.27 (dddd, *J* = 14.4, 10.0, 6.1, 3.5 Hz, 1 H), 2.05 - 1.95 (m, 2 H), 1.85 - 1.78 (m, 2 H), 1.67 - 1.60 (m, 2 H), 1.33 - 1.23 (m, 3 H), 1.17 - 1.04 (m, 3 H), 0.93 - 0.85 (m, 1 H); **^{13}C NMR** (100.6MHz, $CDCl_3$) δ = 134.9, 128.8, 127.3, 127.2, 126.1, 124.7, 122.4, 117.1, 113.2, 111.5, 59.2, 58.1, 43.3, 34.6, 30.7, 29.7, 26.2, 26.0, 25.9; **HRMS-APCI** (MeOH, m/z): $[M+H]^+$ calcd for $C_{23}H_{27}BrNO$, 412.1271; found, 412.1271. **m.p.**: 86 - 87 ^oC; **HPLC** (CHIRALCEL® AD-3, *n*-heptane / EtOH = 95:5, 1 mL/min) t_R = 4.48 min (major), t_R = 5.24 min (minor), 90% *ee* (***S***); $[\alpha]_D^{25}$ = 14.80 (c = 1.00, $CHCl_3$).

8 (*S*)-2-benzyl-5-bromo-1-(1-cyclohexylallyl)-3-phenyl-1*H*-indole **(2h)**

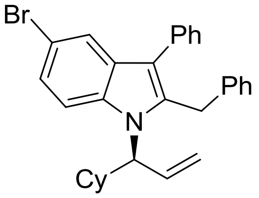

To the crude reaction mixture of **1a** was added 1,3-diphenyl-2-propanone (116 mg, 0.55 mmol). The indolization was proceeded for 18 h at 70 °C. The crude product was purified by FCC on silica gel (CH = 1/30, R_f = 0.34) to afford the product as a white solid (172.0 mg, 71 %).

^{1}H NMR (400MHz, $CDCl_3$) δ = 7.80 (m, 1H), 7.52 - 7.50 (m, 2 H), 7.46 - 7.42 (m, 2 H), 7.35 - 7.21 (m, 6 H), 7.15 - 7.13 (m, 2 H), 6.13 (m, 1 H), 4.96 (m, 1 H), 4.38 (m, 2 H), 4.31 - 4.27 (m, 1 H), 4.14 (d, *J* = 16.8 Hz, 1 H), 2.19 - 2.09 (m, 1 H), 1.94 - 1.91 (m, 1 H), 1.78 - 1.75 (m, 1 H), 1.65 - 1.63 (m, 1 H), 1.55 - 1.52 (m, 1 H), 1.29 - 1.19 (m, 1 H), 1.12 - 0.96 (m, 3 H), 0.88 - 0.78 (m, 1 H), 0.60 - 0.50 (m, 1 H); **^{13}C NMR** (100.6MHz, $CDCl_3$) δ = 138.9, 136.5, 134.8, 133.9, 133.6, 129.8, 129.7, 128.6, 128.6, 128.6, 126.5, 126.5, 123.9, 121.9, 118.4, 116.0, 113.5, 112.9, 64.5, 40.0, 31.6, 31.4, 29.7, 26.2, 26.0, 25.9; **HRMS-APCI** (MeOH, m/z): $[M+H]^+$ calcd for $C_{30}H_{31}{}^{79}BrN$, 484.1634; found, 484.1638. **m.p.**: 83 - 84 ^oC; **HPLC** (CHIRALCEL® L-C3 + OJ-R, MeCN / H_2O = 75:25, 0.5 mL/min) t_R = 37.29 min (major), t_R = 42.09 min (minor), 91% *ee* (***S***); $[\alpha]_D^{25}$ = + 12.54 (c = 1.005, $CHCl_3$).

9 (*S*)-8-bromo-5-(1-cyclohexylallyl)-1,3,4,5-tetrahydrothiopyrano[4,3-*b*]indole **(2i)**

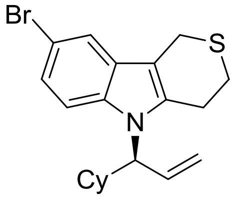

To the crude reaction mixture of **1a** was added tetrahydro-4*H*-thiopyran-4-one (63.9 mg, 0.55 mmol). The indolization was proceeded for 18 h at 100 °C. The crude product was purified by FCC on silica gel (Tol/CH = 1/9, R_f = 0.29) to afford the product as a yellowish oil (85.1 mg, 44 %).

^{1}H NMR (400MHz, $CDCl_3$) δ = 7.57 (dd, *J* = 1.9, 0.6 Hz, 1 H), 7.22 (dd, *J* = 8.7, 0.6 Hz, 1 H), 7.18 (dd, *J* = 8.7, 1.9 Hz, 1 H), 6.25 (ddd, *J* = 17.0, 10.4, 6.6 Hz, 1 H), 5.19 (dt, *J* = 10.4, 1.3 Hz, 1 H), 4.98 (dt, *J* = 17.0, 1.3 Hz, 1 H), 4.32 (dd, *J* = 10.4, 6.6 Hz, 1 H), 3.82 (m, 2 H), 3.04 - 2.90 (m, 4 H), 2.19 - 2.09 (m, 1 H), 2.08 - 2.01 (m, 1 H), 1.85 - 1.78 (m, 1 H), 1.70 - 1.63 (m, 1 H), 1.60 - 1.54 (m, 1 H), 1.35 - 1.24 (m, 1 H), 1.18 - 0.96 (m, 4 H), 0.77 - 0.66 (m, 1 H),; **^{13}C NMR** (100.6MHz, $CDCl_3$) δ = 136.1, 134.7, 133.0, 129.0, 123.5, 120.3, 118.1, 112.7, 112.2, 106.2, 63.5, 40.1, 31.5, 29.9, 26.2, 26.0, 25.9, 25.8, 25.3, 23.0; **HRMS-APCI** (MeOH, m/z): $[M+H]^+$ calcd for $C_{20}H_{25}{}^{79}BrNS$, 390.0886; found, 390.0888. **HPLC** (CHIRALCEL® OJ-3R, MeCN / H_2O = 60:40, 0.5 mL/min) t_R = 49.36 min (minor), t_R = 55.45 min (major), 90% *ee* (***S***); $[\alpha]_D^{25}$ = + 63.90 (c = 0.975, $CHCl_3$).

One-pot Late-stage Indolization of (+)-Testosterone acetate

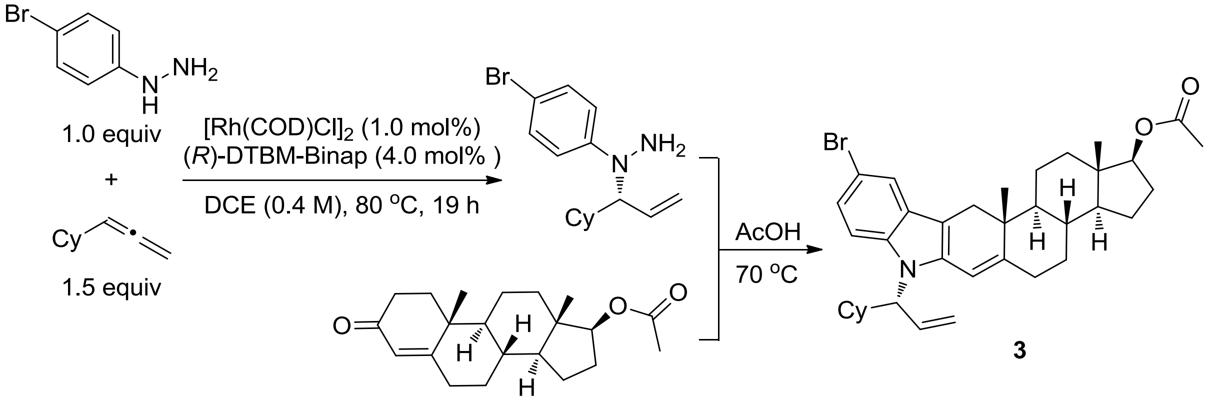

3

A 25 ml screw-cap Schlenk rond bottom flask was flame-dried under vacuum, backfilled with argon and cooled to room temperature using a standard S4 Schlenk line apparatus. To the screw-cap Schlenk flask was charged with $[Rh(COD)Cl]_2$ (14.8 mg, 0.03 mmol, 1 mol%), (*R*)-DTBM-Binap (143 mg, 0.12 mmol, 4 mol%), *p*-bromophenylhydrazine (561.1 mg, 3.0 mmol, 1.0 equiv), 1,2-dichloroethane (7.5 ml, 0.4 M) and cyclohexylallene (655.5 µl, 4.5 mmol, 1.5 equiv). The flask was then sealed and the mixture was stirred for 19 h at 80 °C. After cooling to room temperature, acetyl testosterone (1.1 g, 3.3 mmol, 1.1 equiv) was added to the reaction mixture and stirred for 30 min to form the corresponding hydrazone. The solvent was removed by rotary evaporation, and then acetic acid (12 ml, 0.25 M) was added. After stirring for 18 h at 70 °C, the volatiles were removed under vacuum, and the crude product was purified by FCC on silica gel (EA/CH = 1/30, R_f = 0.24) to afford the product as a yellow solid (1.06 g, 59 %, *d.r.* = 1/17, determined by ^{1}H NMR).

^{1}H NMR (400MHz, $CDCl_3$, mixture of two diastereomers) δ = 7.96 (d, *J* = 1.8 Hz, 1 H), 7.22 (d, *J* = 8.7 Hz, 1 H), 7.18 (dd, *J* = 8.7, 1.8 Hz, 1 H), 6.28 (ddd, *J* = 17.0, 10.4, 6.6 Hz, 1 H, minor), 6.24 (ddd, *J* = 17.0, 10.3, 6.5 Hz, 1 H, major), 6.09 - 6.08 (m, 1 H), 5.21 (dt, *J* = 10.4, 1.3 Hz, 1 H, minor), 5.17 (dt, *J* = 10.3, 1.3 Hz, 1 H, major), 5.04 (dt, *J* = 17.0, 1.3 Hz, 1 H, minor), 4.96 (dt, *J* = 17.0, 1.3 Hz, 1 H, major), 4.66 (dd, *J* = 9.1, 7.7 Hz, 1 H), 4.39 - 4.34 (m, 1 H), 2.86 - 2.81 (m, 1 H), 2.73 - 2.64 (m, 1 H), 2.41 - 2.35 (m, 1 H), 2.26 - 2.02 (m, 5 H), 2.06 (s, 3H), 1.85 - 1.62 (m, 8 H), 1.61 - 1.52 (m, 4 H), 1.50 - 1.35 (m, 2 H), 1.34 - 1.22 (m, 2 H), 1.20 - 1.00 (m, 7 H), 0.98 (s, 3H), 0.87 (s, 3H), 0.79 - 0.68 (m, 1 H); **^{13}C NMR** (100.6MHz, $CDCl_3$, 323 K) (major) δ = 171.1, 137.7, 136.5, 134.8, 127.6, 123.1, 122.5, 117.9, 116.3, 112.7, 109.8, 82.7, 63.5, 51.2, 48.4, 42.4, 39.9, 36.9, 36.4, 34.6, 31.7, 31.4, 31.4, 29.8, 27.6, 26.2, 25.9, 25.7, 23.6, 21.1, 21.0, 20.8, 18.4, 12.0; (minor) δ = 171.1, 137.7, 136.7, 134.5, 127.7, 123.0, 122.5, 118.2, 116.4, 112.7,

109.6, 82.7, 63.5, 51.2, 48.3, 42.4, 39.9, 36.9, 36.3, 34.7, 31.7, 31.4, 31.4, 29.7, 27.6, 26.2, 25.9, 25.8, 23.6, 21.1, 21.0, 20.8, 18.4, 12.0; **HRMS-APCI** (m/z): $[M+H]^+$ calcd for $C_{36}H_{47}{}^{79}BrNO_2$, 604.2785; found, 604.2786. **m.p.**: 190 - 191 °C; $[\alpha]_D^{25}$ = + 9.95 (c = 0.995, $CHCl_3$).

Mechanistic Investigations

Reaction of 1-methyl-1-phenylhydrazine with cyclohexylallene

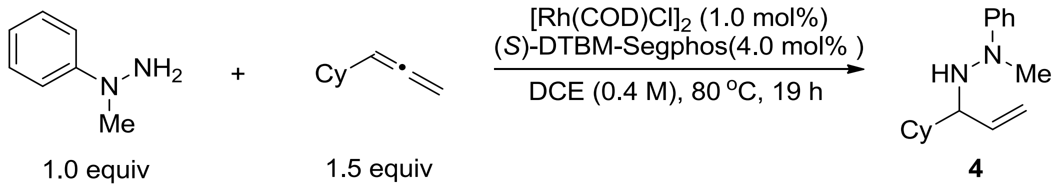

The reaction was performed in a 5.0 ml Schlenk tube under argon. $[Rh(COD)Cl]_2$ (2.5 mg, 0.005 mmol, 1.0 mol%), (*S*)-DTBM-Segphos (23.6 mg, 0.02 mmol, 4.0 mol%), and 1-methyl-1-phenylhydrazine (61.1 mg, 0.5 mmol, 1.0 equiv) were dissolved in DCE (1.25 mL), then cyclohexylallene (91.6 mg, 0.75 mmol, 1.5 equiv.) was added and the tube was sealed. The reaction mixture was stirred at 80 °C for 19 hours. After cooling to room temperature, the solvent was removed by rotary evaporation. The crude reaction mixture was analyzed by ^{1}H NMR, which indicated only trace of product formation.

Isotopic-labeling experiment with [D_3]phenyl hydrazine

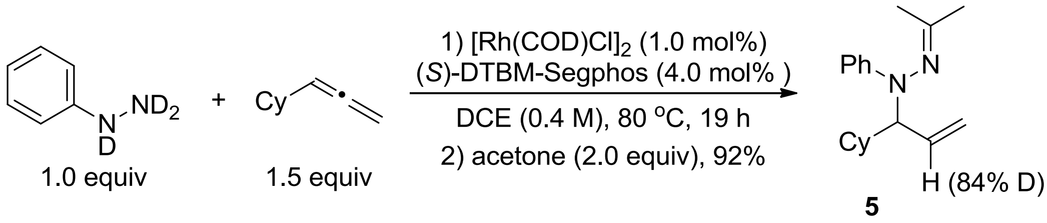

The reaction was performed in a 5.0 ml Schlenk tube under argon. $[Rh(COD)Cl]_2$ (2.5 mg, 0.005 mmol, 1.0 mol%), (*S*)-DTBM-Segphos (23.6 mg, 0.02 mmol, 4.0 mol%), and [D_3]Phenylhydrazine (55.6 mg, 0.5 mmol, 1.0 equiv) were dissolved in DCE (1.25 mL), then cyclohexylallene (91.6 mg, 0.75 mmol, 1.5 equiv.) was added and the tube was sealed. The reaction mixture was stirred at 80 °C for 19 hours. After cooling to room temperature, the solvent was removed by rotary evaporation. To the reaction residue was added acetone (74 µL, 58.1 mg, 1.0 mmol, 2.0 equiv) to mask the -ND_2 group via hydrazone formation. The crude product was purified by flash column chromatography on silica gel (EA / CH = 1:30, R_f = 0.35) to afford the product as a yellowish oil (125.0 mg, 92%).

The corresponding non-deuterated hydrazone of **5** was prepared by reaction of **1a** with acetone. Analytical data of the non-deuterated hydrazone of **5**. **^{1}H NMR** (400 MHz, $CDCl_3$) δ = 7.21 - 7.15 (m, 2 H), 6.82 (tt, *J* = 1.2, 7.3 Hz, 1 H), 6.79 - 6.74 (m, 2 H), 6.11 (ddd, *J* = 9.1, 10.3, 17.4 Hz, 1 H), 5.04 - 4.93 (m, 1 H), 4.74 (ddd, *J* = 0.7, 2.2, 17.3 Hz, 1 H), 3.54 (t, *J* = 9.4 Hz, 1 H), 2.08 (s, 3 H), 2.01 - 1.93 (m, 1 H), 1.80 - 1.55 (m, 8 H), 1.31 - 1.16 (m, 3 H), 1.09 - 0.97 (m, 1 H), 0.94 - 0.81 (m, 1 H); **^{13}C NMR** (101 MHz, $CDCl_3$) δ = 167.0, 151.3, 137.0, 128.7, 120.6, 119.6, 117.0, 39.6, 30.9, 30.8, 26.9, 26.3, 26.2, 25.1, 20.0; **HRMS-APCI** (MeOH, m/z): $[M+H]^+$ $C_{16}H_{27}N_2$, 271.21688; found, 272.21698.

Mass analysis of **5** gave the following deuterium distribution

**d_n** (Number of deuterium incorporated)	**Relative Distribution** (%)
**d_0**	18
**d_1**	82

Stoichiometric Reaction of phenylhydrazine with catalysts

To a dry J. Young NMR tube was added $[Rh(COD)Cl]_2$ (24.6 mg, 0.05 mmol), DPEphos (53.9 mg, 0.1 mmol) and phenylhydrazine (10.8 mg, 0.1 mmol), then $CDCl_3$ (1.0 ml) was added. The tube was sealed under argon and shaked for 5 min. The sample was analysed by NMR at 263K.

Supplementary References

1. Coleman, G. H. Phenylhydrazine. *Org. Synth*. **2**, 71, (1941).
2. Taherirastgar, F., Brandsma, L. Conversion of Alkyl- and Arylallenes into 1-Alkynes through Metallated Intermediates. *Synth. Commun.* **27**, 4035, (1997).
3. Xu, K., Thieme, N., Breit, B. Atom-Economic, Regiodivergent, and Stereoselective Coupling of Imidazole Derivatives with Terminal Allenes. *Angew. Chem. Int. Ed*. **53**, 2162, (2014).
4. Kuang, J., Ma, S. An Efficient Synthesis of Terminal Allenes from Terminal 1-Alkynes. *J. Org. Chem.* **74**, 1763, (1999).
5. Johns, A. M., Liu, Z., Hartwig, J. F. Primary *tert*- and *sec*-Allylamines via Palladium-Catalyzed Hydroamination and Allylic Substitution with Hydrazine and Hydroxylamine Derivatives. *Angew. Chem. Int. Ed*. **46**, 7259, (2007).
6. Cooke, M. L., Xu, K., Breit, B. Enantioselective Rhodium-Catalyzed Synthesis of Branched Allylic Amines by Intermolecular Hydroamination of Terminal Allenes. *Angew. Chem. Int. Ed*. **51**, 10876, (2012).
7. Bailey, W. F., Luderer, M. R., Jordan, K. P. Effect of Solvent on the Lithium-Bromine Exchange of Aryl Bromides: Reactions of *n*-Butyllithium and *tert*-Butyllithium with 1-Bromo-4-*tert*-butylbenzene at 0 ^{o}C. *J. Org. Chem*. **71**, 2825, (2006).
8. Akwabi-Ameyaw, A., Caravella, J. A., Chen, L., Creech, K. L., Deaton, D. N., Madauss, K. P., Marr, H. B., Miller, A. B., Navas III, F., Parks, D. J., Spearing, P. K., Todd, D., Williams, S. P., Wisely, G. B. Conformationally Constrained Farnesolid X Receptor (FXR) Agonists: Alternative Replacements of the Stilbene. *Bioorg. Med. Chem. Lett*. **21**, 6154, (2011).
9. Stanley, L. M., Hartwig, J. F. Iridium-Catalyzed Regio- and Enantioselective N-Allylation of Indoles. *Angew. Chem. Int. Ed.* **48**, 7841, (2009).
